# Supplementary material for: The Construction of Polycyclic Pyridones via Ring-Opening Transformations of 3-hydroxy-3,4-dihydropyrido[2,1-c][1,4]oxazine-1,8-diones
Source: Molecules. 2023 Jan 28;28(3):1285. doi: 10.3390/molecules28031285 (PMC9921744; doi:10.3390/molecules28031285)
Supplement: Supplementary file 1 [file molecules-28-01285-s001.zip › molecules-2168638-supplementary.pdf]

# Supplementary materials

## The construction of polycyclic pyridones *via* ring-opening transformations of 3-hydroxy-3,4-dihydropyrido[2,1-*c*][1,4]oxazine-1,8-diones

Viktoria V. Viktorova, Elena V. Steparuk, Dmitrii L. Obydennov,* and Vyacheslav Y. Sosnovskikh*

Institute of Natural Sciences and Mathematics, Ural Federal University, 51 Lenina Ave., 620000 Ekaterinburg, Russia
* Correspondence: vy.sosnovskikh@urfu.ru; dobydennov@mail.ru; Tel.: +7-343-3899597

## Table of Contents

$^{1}H$ and $^{13}C$ NMR spectra of compounds **2** ........ S2
$^{1}H$ and $^{13}C$ NMR spectra of compounds **3** ........ S13
$^{1}H$ and $^{13}C$ NMR spectra of compounds **4** ........ S25
$^{1}H$ and $^{13}C$ NMR spectra of compounds **5** ........ S33
$^{1}H$ and $^{13}C$ NMR spectra of compounds **6** ........ S39
$^{1}H$ NMR spectrum of the reaction mixture of **3b** with $NH_4OAc$ ........ S47
HRMS spectra of compounds **2a,e**, **3a,c,e,f**, **4a–d**, **5a–c**, **6a,b** ........ S48

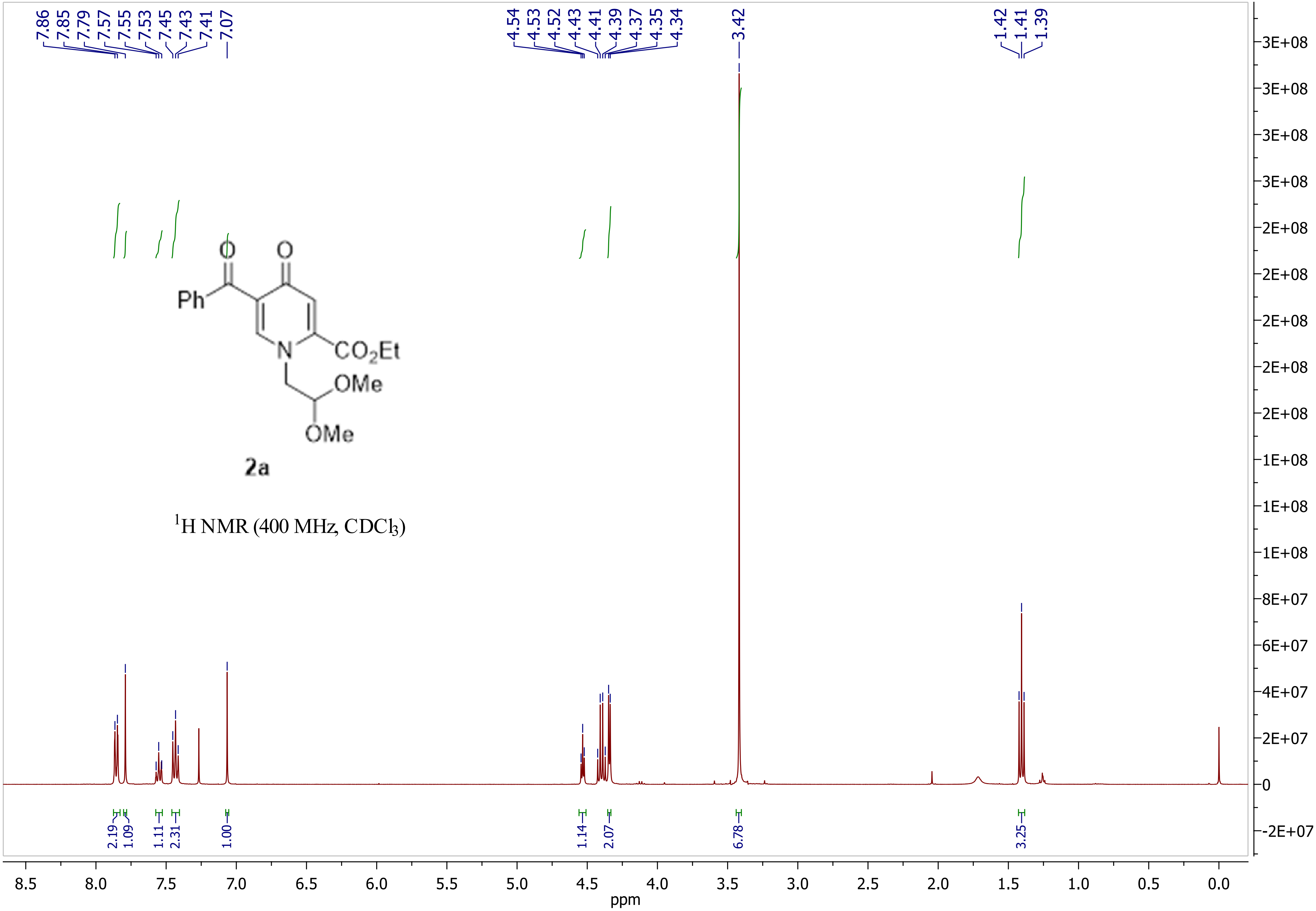
7.86
7.85
7.79
7.57
7.55
7.53
7.45
7.43
7.41
7.07
4.54
4.53
4.52
4.43
4.41
4.39
4.37
4.35
4.34
3.42
1.42
1.41
1.39
Ph
CO2Et
OMe
OMe
2a
1H NMR (400 MHz, CDCl3)
2.19
1.09
1.11
2.31
1.00
1.14
2.07
6.78
3.25
ppm

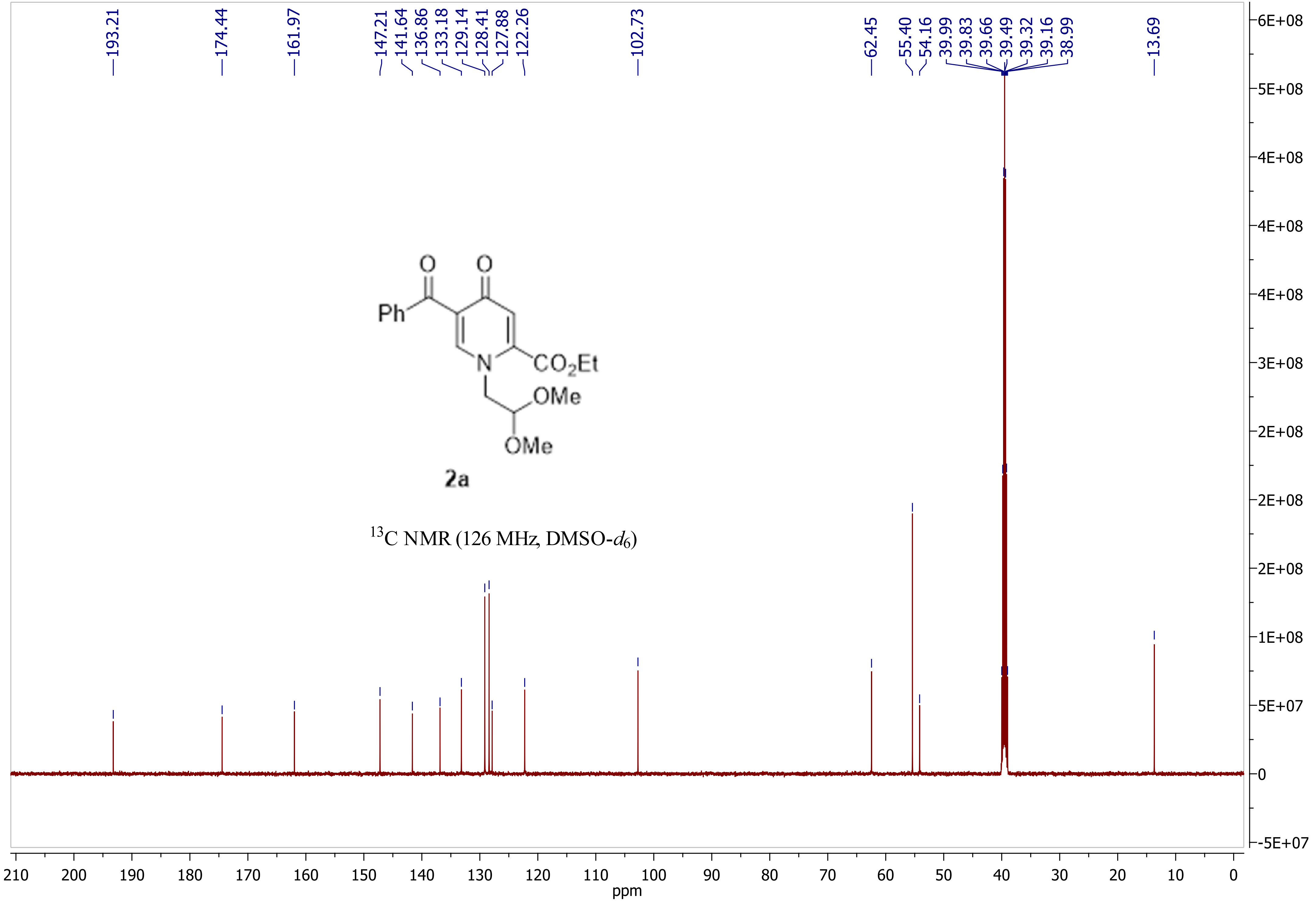


$^{13}$C NMR (126 MHz, DMSO-$d_6$)

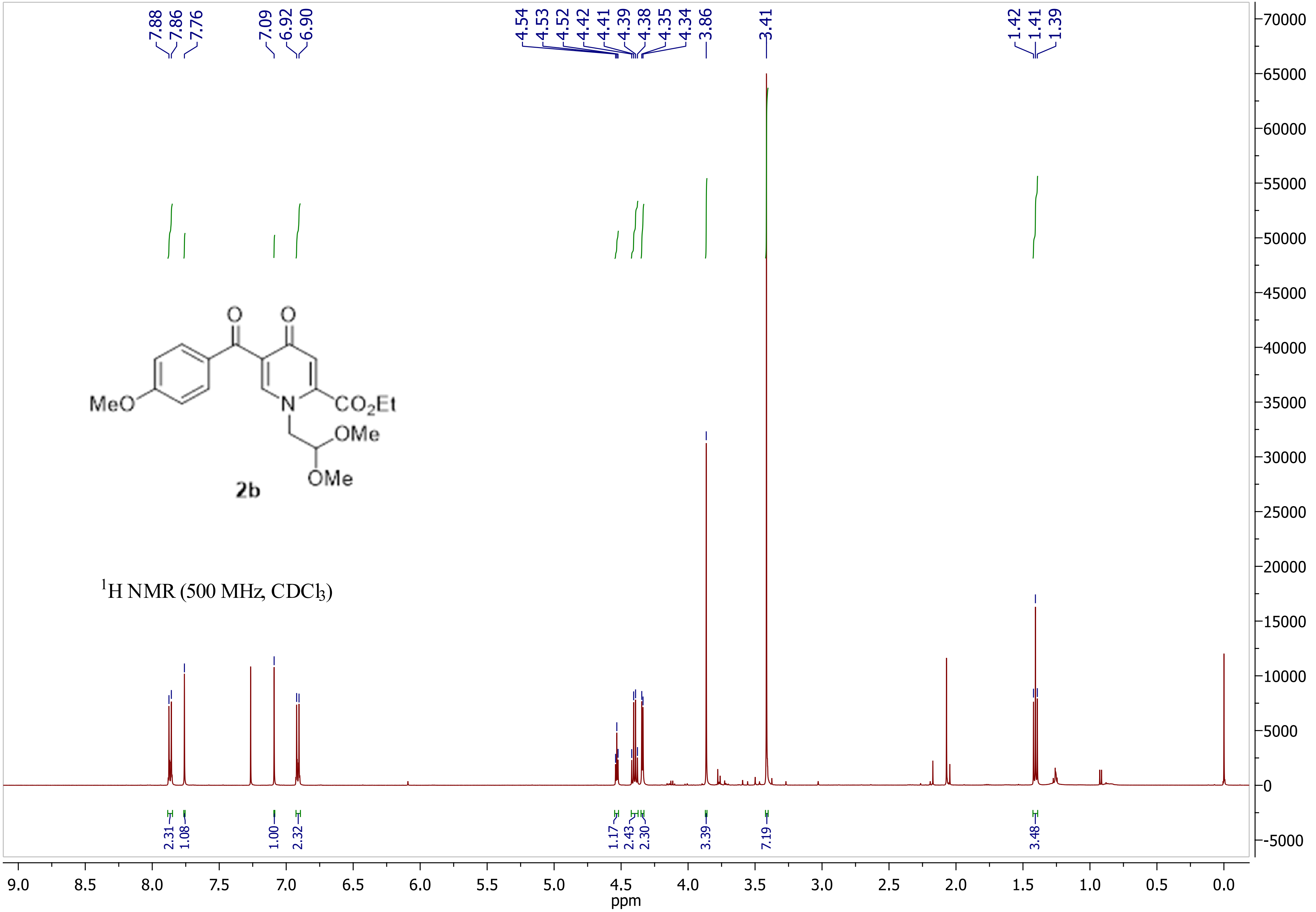

7.88
7.86
7.76
7.09
6.92
6.90
4.54
4.53
4.52
4.42
4.41
4.39
4.38
4.35
4.34
3.86
3.41
1.42
1.41
1.39
MeO
CO2Et
N
OMe
OMe
2b
1H NMR (500 MHz, CDCl3)
2.31
1.08
1.00
2.32
1.17
2.43
2.30
3.39
7.19
3.48
ppm

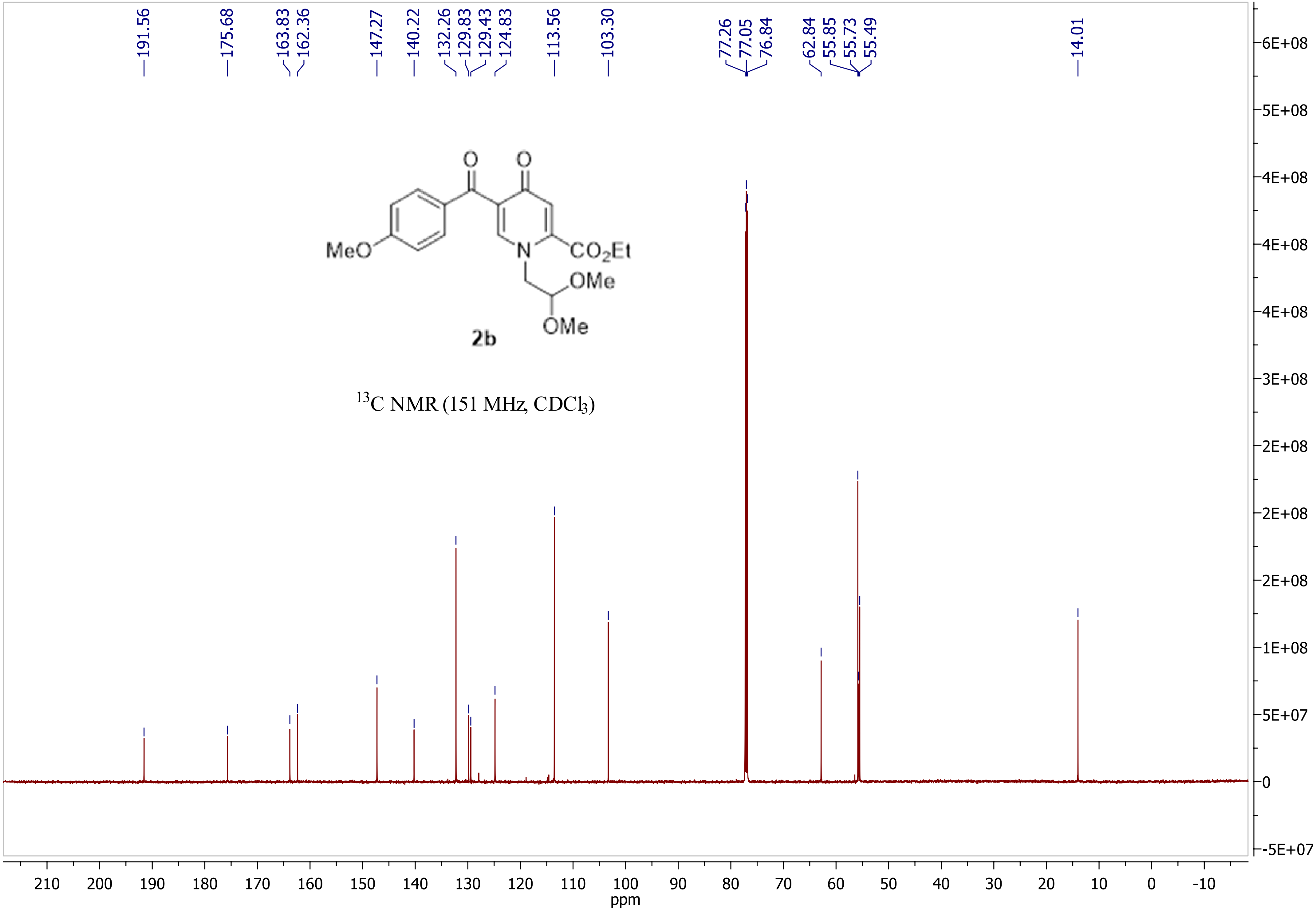
191.56
175.68
163.83
162.36
147.27
140.22
132.26
129.83
129.43
124.83
113.56
103.30
77.26
77.05
76.84
62.84
55.85
55.73
55.49
14.01
MeO
CO2Et
OMe
OMe
N
O
O
2b
13C NMR (151 MHz, CDCl3)
ppm
6E+08
5E+08
4E+08
3E+08
2E+08
1E+08
5E+07
0
-5E+07

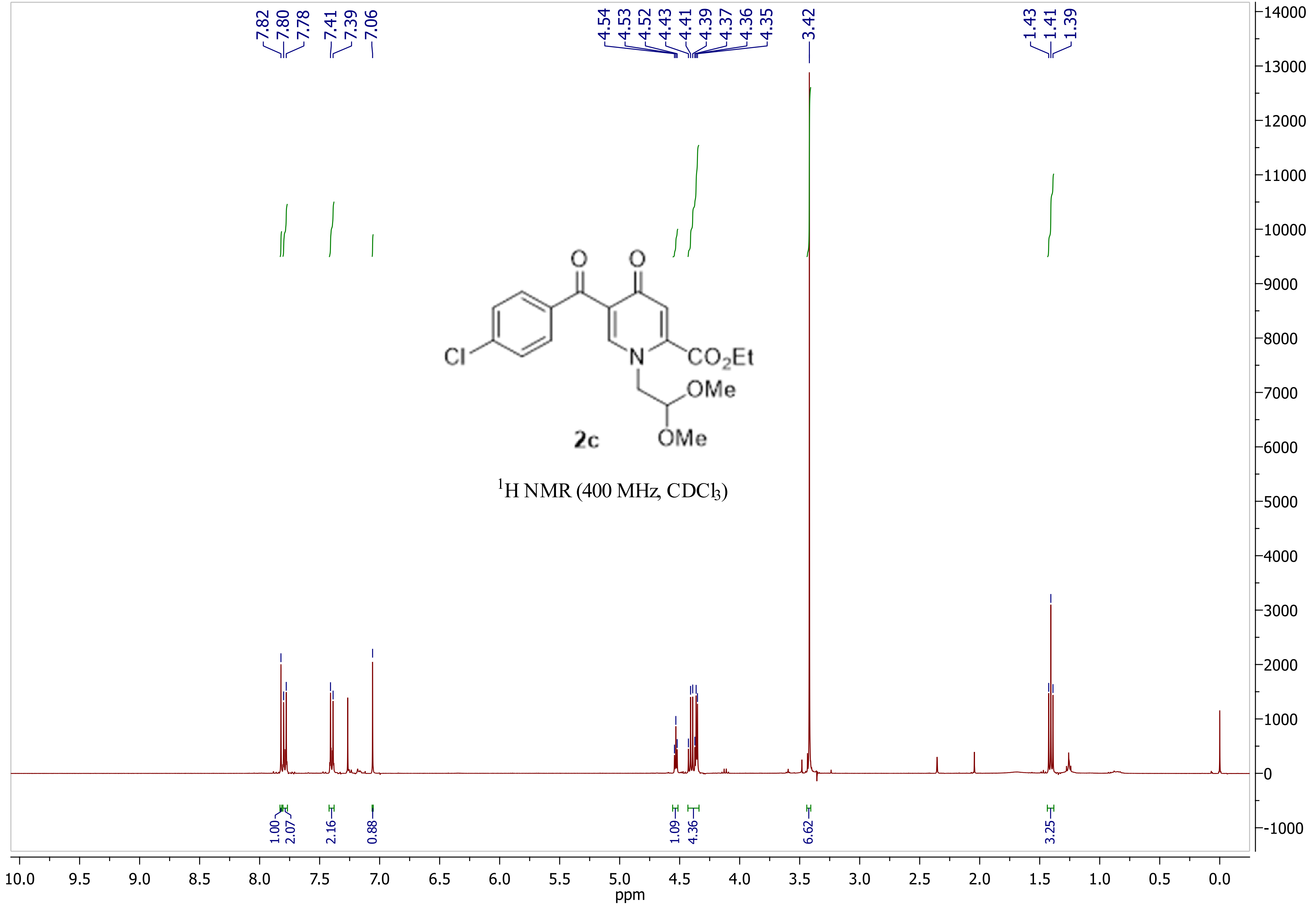
7.82
7.80
7.78
7.41
7.39
7.06
4.54
4.53
4.52
4.43
4.41
4.39
4.37
4.36
4.35
3.42
1.43
1.41
1.39
Cl
O
O
N
CO2Et
OMe
OMe
2c
1H NMR (400 MHz, CDCl3)
1.00
2.07
2.16
0.88
1.09
4.36
6.62
3.25
ppm
10.0 9.5 9.0 8.5 8.0 7.5 7.0 6.5 6.0 5.5 5.0 4.5 4.0 3.5 3.0 2.5 2.0 1.5 1.0 0.5 0.0
14000 13000 12000 11000 10000 9000 8000 7000 6000 5000 4000 3000 2000 1000 0 -1000

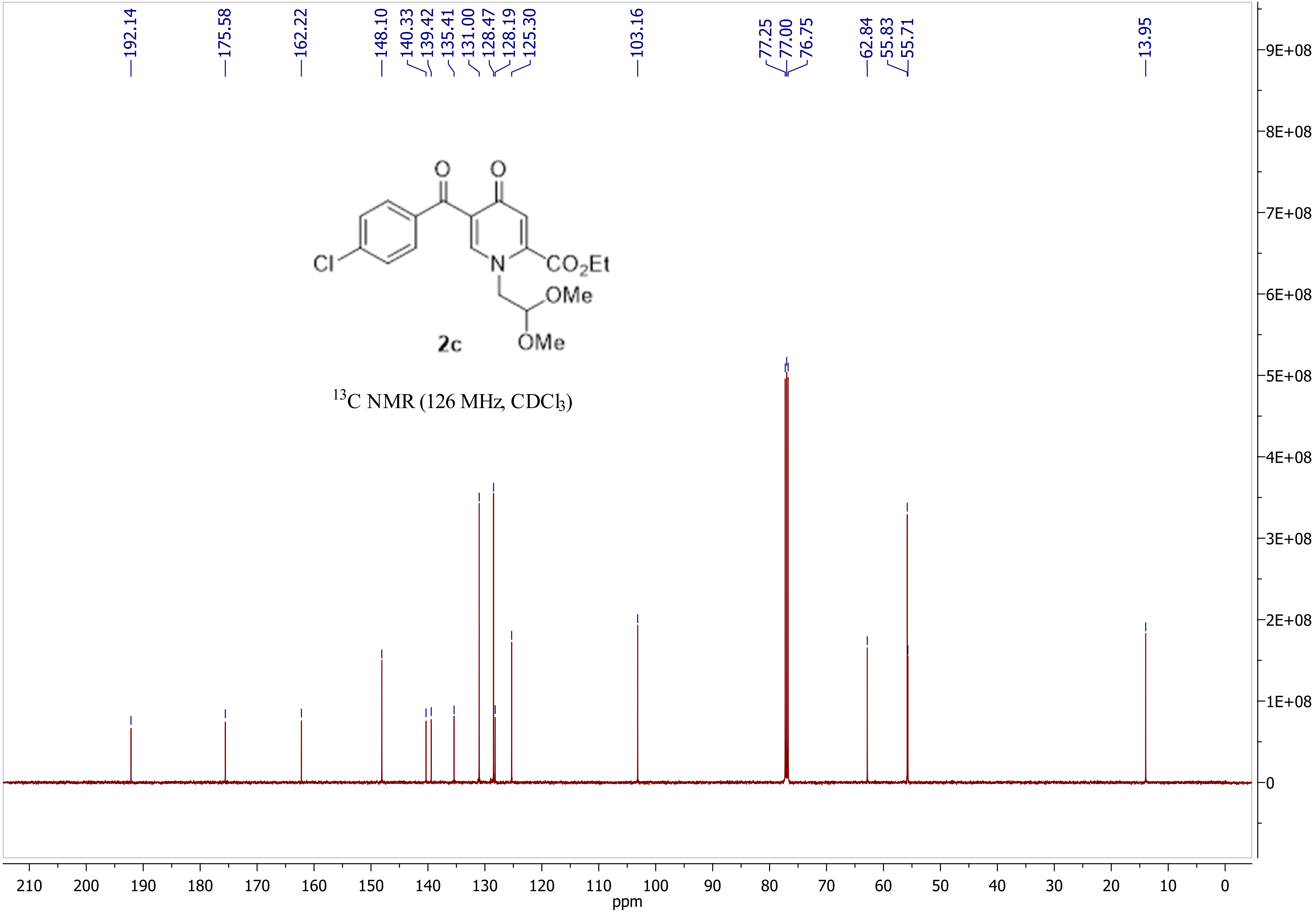


$^{13}C$ NMR (126 MHz, $CDCl_3$)

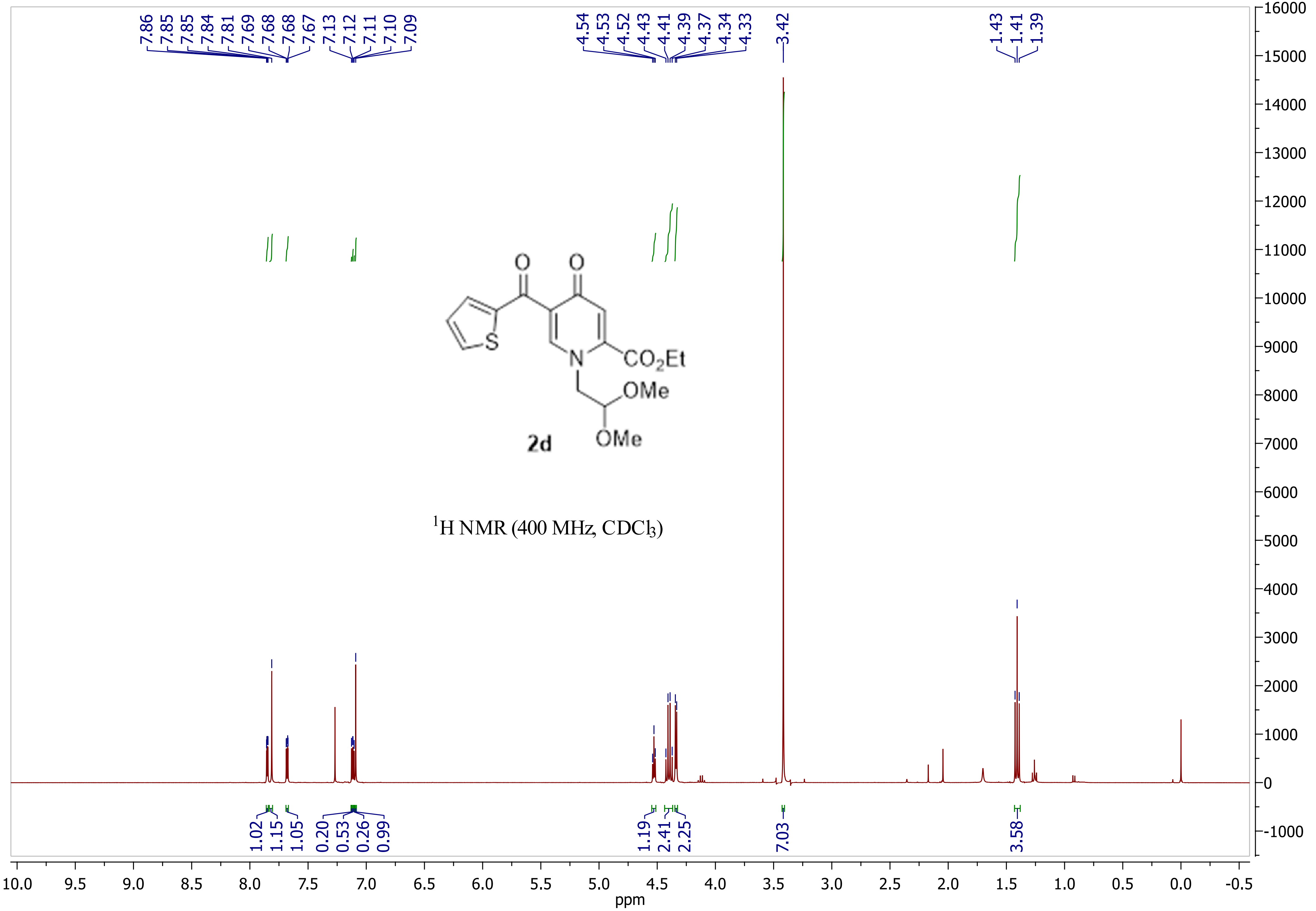
7.86 7.85 7.85 7.84 7.81 7.69 7.68 7.68 7.67 7.13 7.12 7.11 7.10 7.09
4.54 4.53 4.52 4.43 4.41 4.39 4.37 4.34 4.33
3.42
1.43 1.41 1.39
O
O
S
N
CO2Et
OMe
OMe
2d
1H NMR (400 MHz, CDCl3)
1.02 1.15 1.05 0.20 0.53 0.26 0.99
1.19 2.41 2.25
7.03
3.58
ppm

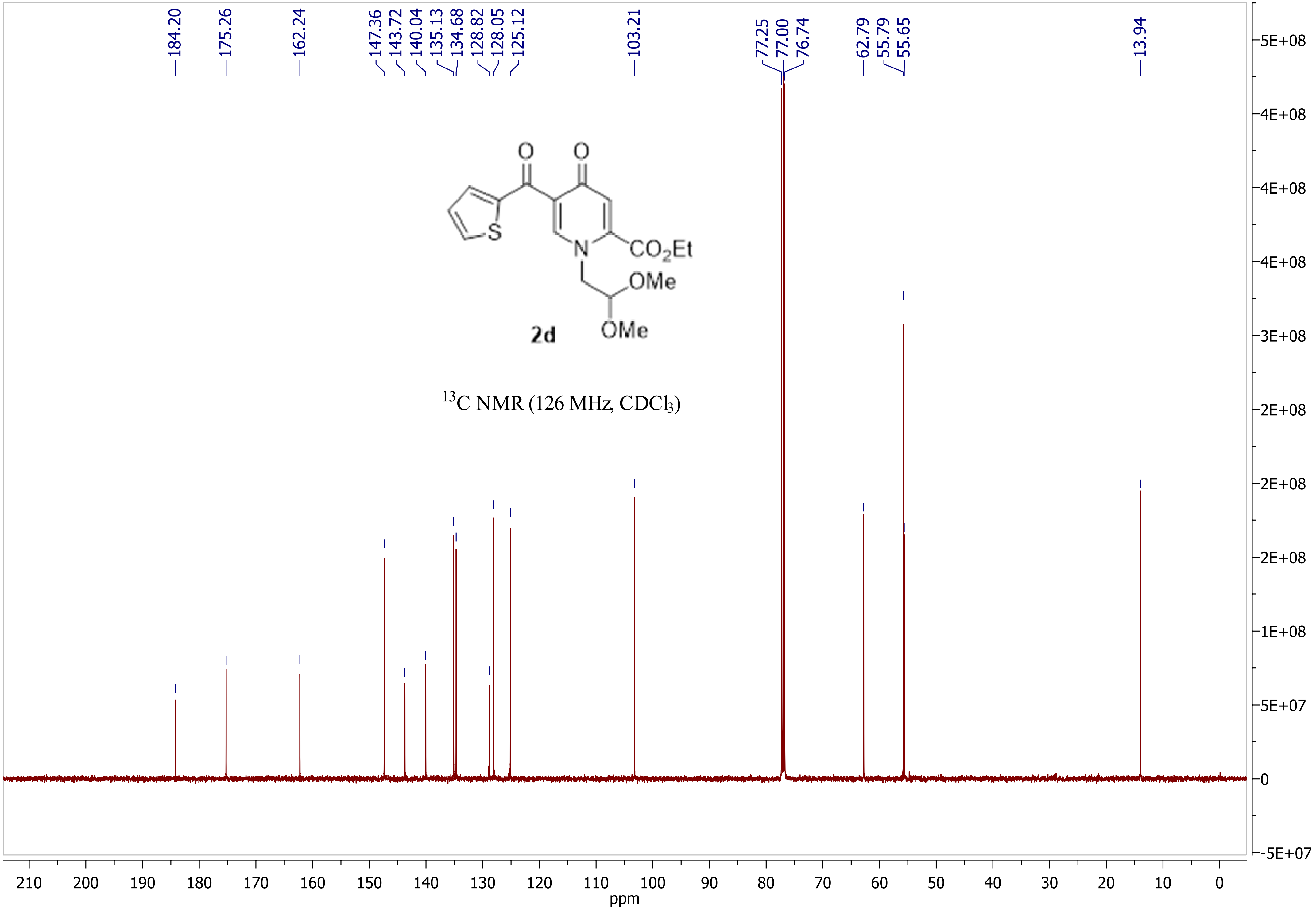
184.20
175.26
162.24
147.36
143.72
140.04
135.13
134.68
128.82
128.05
125.12
103.21
77.25
77.00
76.74
62.79
55.79
55.65
13.94
CO2Et
OMe
OMe
2d
13C NMR (126 MHz, CDCl3)
ppm

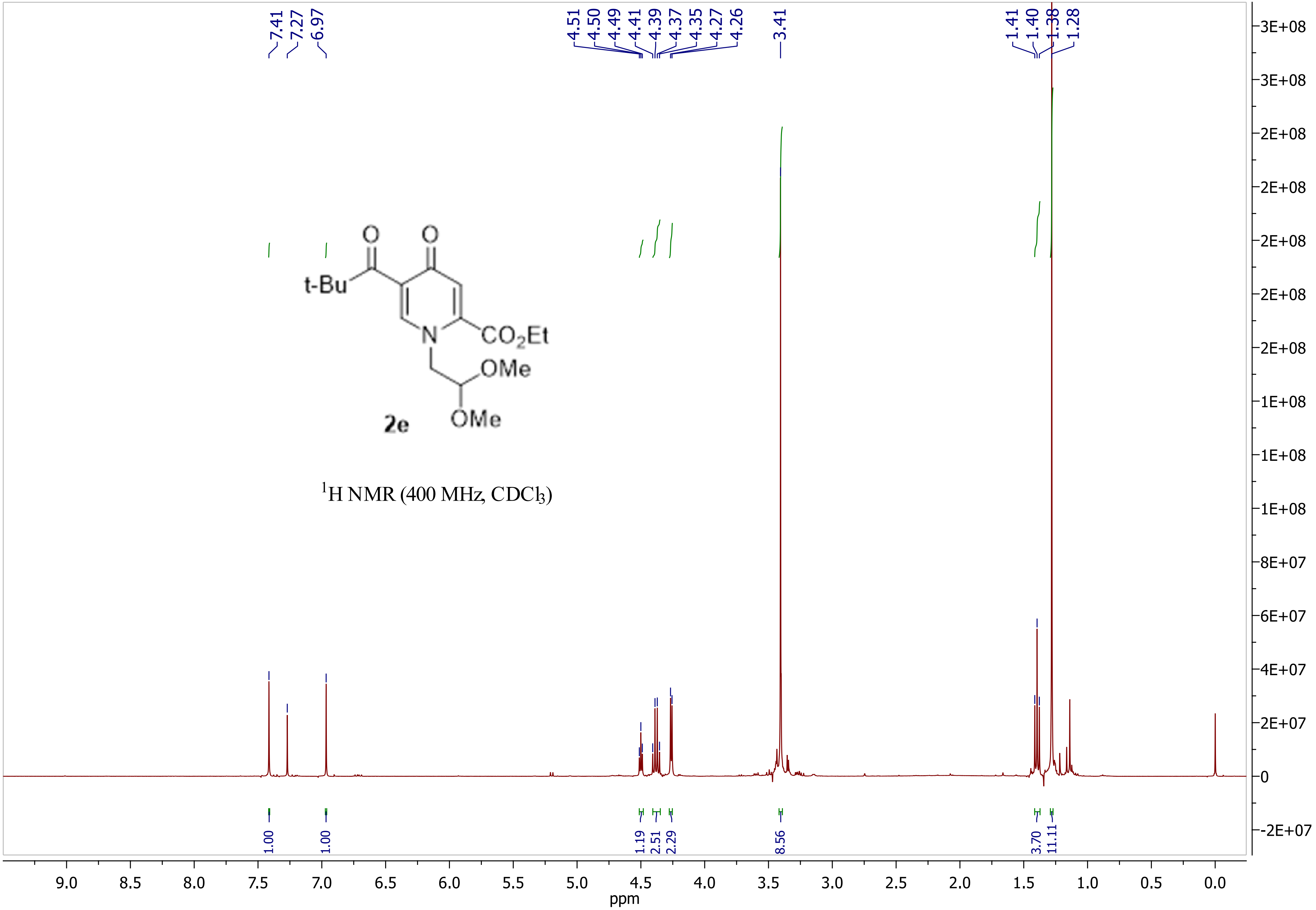


$^1$H NMR (400 MHz, $CDCl_3$)

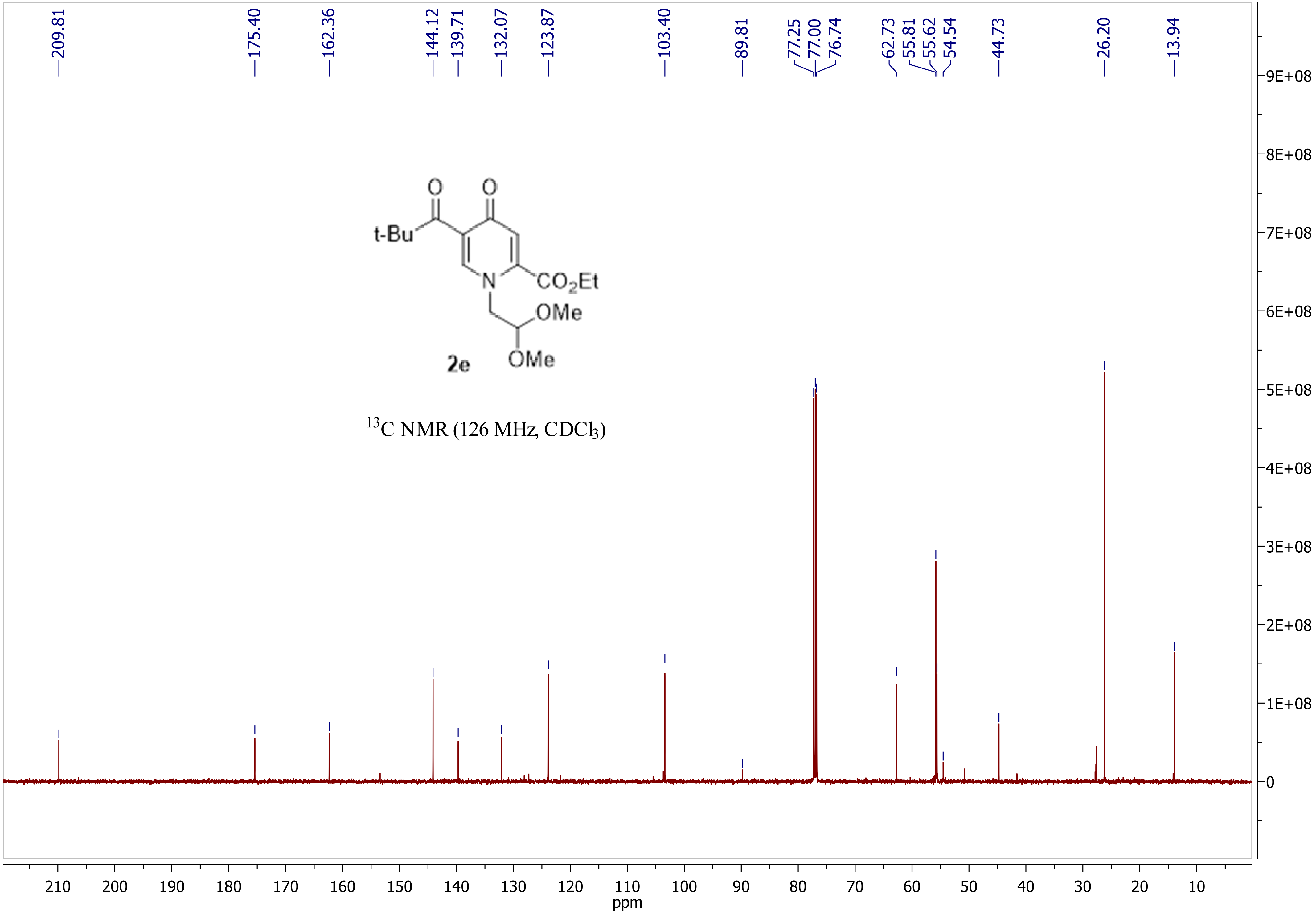
209.81
175.40
162.36
144.12
139.71
132.07
123.87
103.40
89.81
77.25
77.00
76.74
62.73
55.81
55.62
54.54
44.73
26.20
13.94
t-Bu
CO2Et
OMe
OMe
2e
13C NMR (126 MHz, CDCl3)
9E+08
8E+08
7E+08
6E+08
5E+08
4E+08
3E+08
2E+08
1E+08
0
210 200 190 180 170 160 150 140 130 120 110 100 90 80 70 60 50 40 30 20 10
ppm

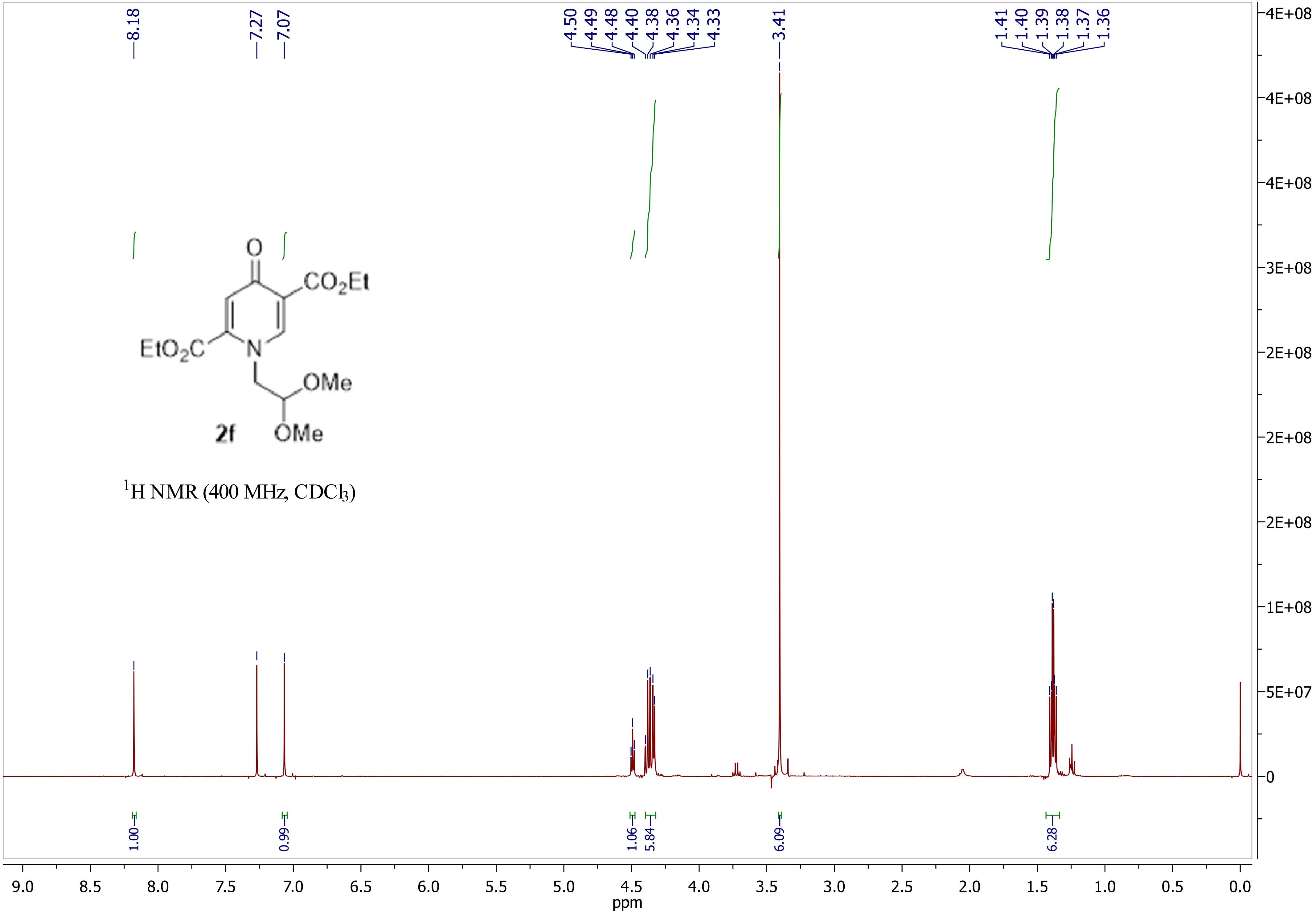
8.18
7.27
7.07
4.50 4.49 4.48 4.40 4.38 4.36 4.34 4.33
3.41
1.41 1.40 1.39 1.38 1.37 1.36
O
CO2Et
EtO2C
N
OMe
OMe
2f
1H NMR (400 MHz, CDCl3)
1.00
0.99
1.06
5.84
6.09
6.28
ppm
9.0 8.5 8.0 7.5 7.0 6.5 6.0 5.5 5.0 4.5 4.0 3.5 3.0 2.5 2.0 1.5 1.0 0.5 0.0
4E+08
4E+08
4E+08
3E+08
2E+08
2E+08
2E+08
1E+08
5E+07
0

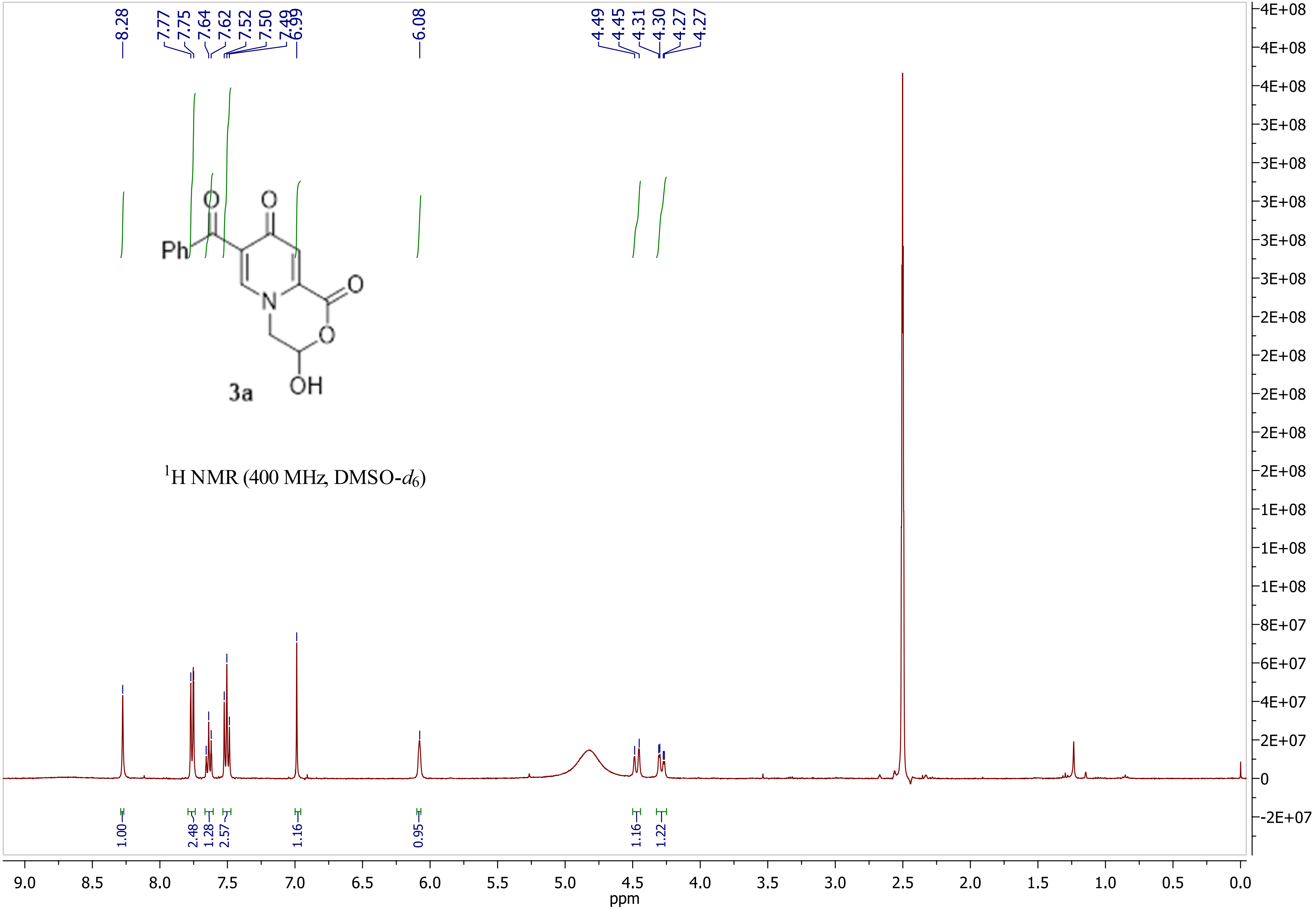


$^1$H NMR (400 MHz, DMSO-$d_6$)

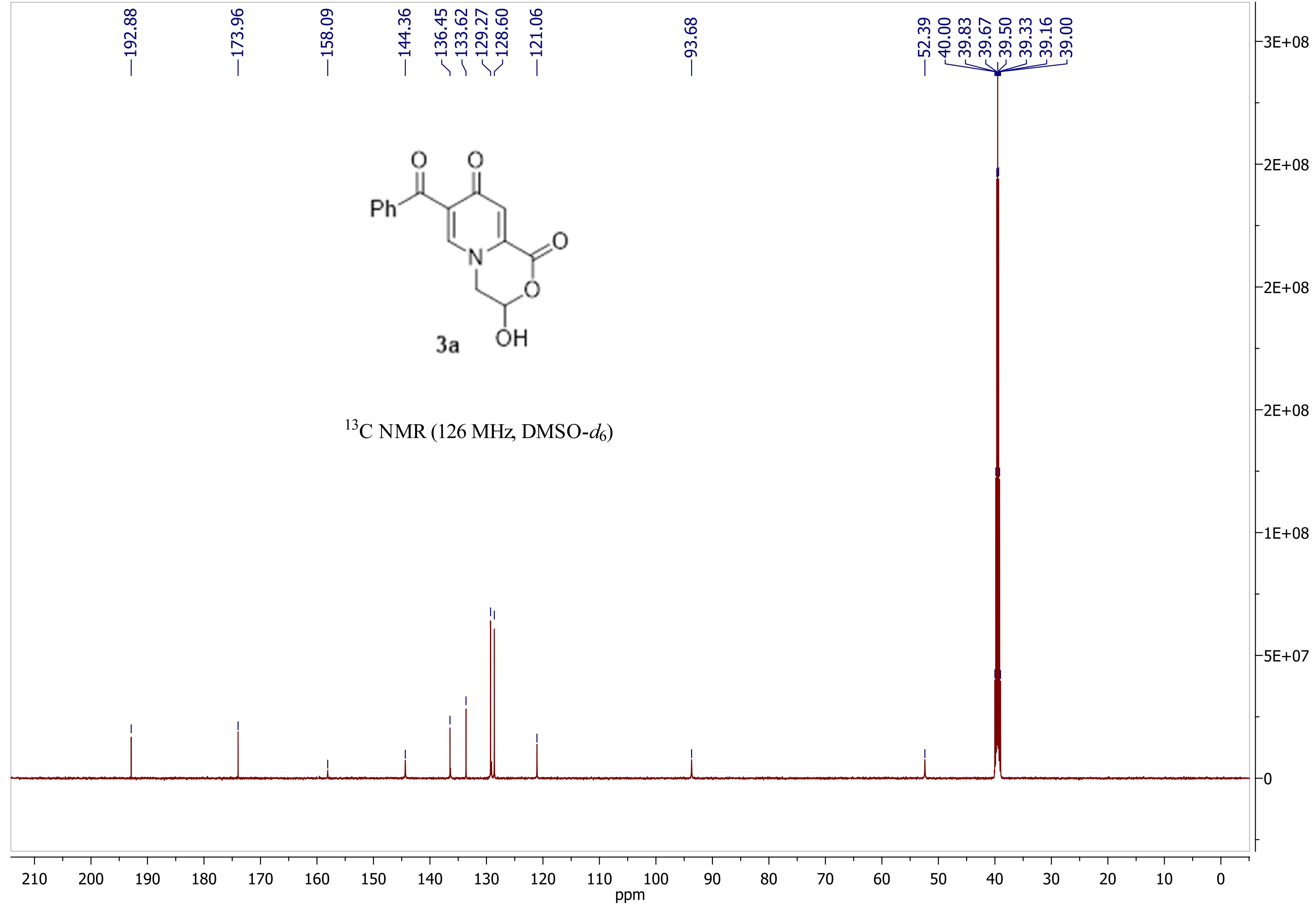
192.88
173.96
158.09
144.36
136.45
133.62
129.27
128.60
121.06
93.68
52.39
40.00
39.83
39.67
39.50
39.33
39.16
39.00
Ph
O
N
OH
3a
$^{13}$C NMR (126 MHz, DMSO-$d_6$)
3E+08
2E+08
2E+08
2E+08
1E+08
1E+08
5E+07
0
210
200
190
180
170
160
150
140
130
120
110
100
90
80
70
60
50
40
30
20
10
0
ppm

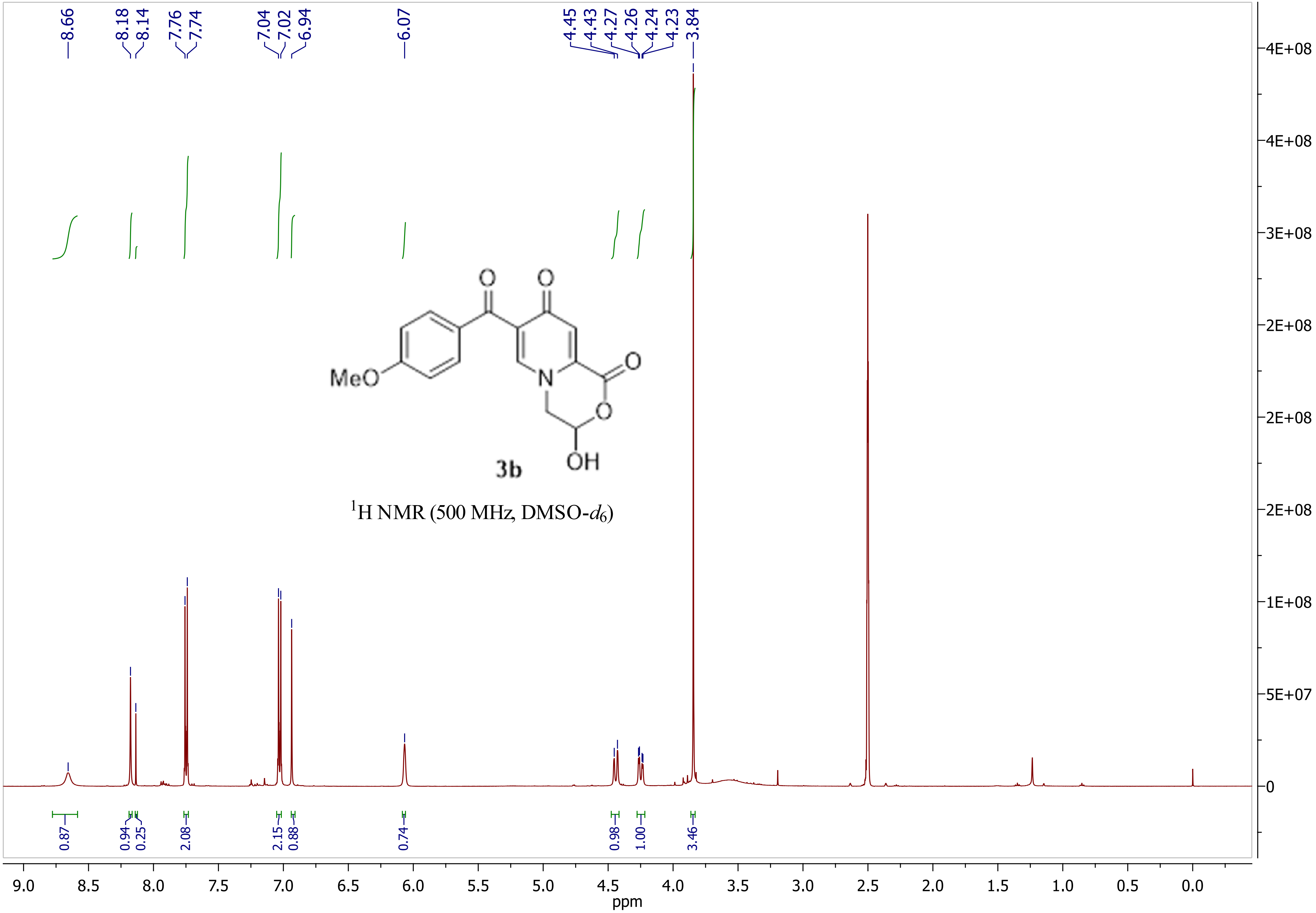


$^{1}$H NMR (500 MHz, DMSO-$d_6$)

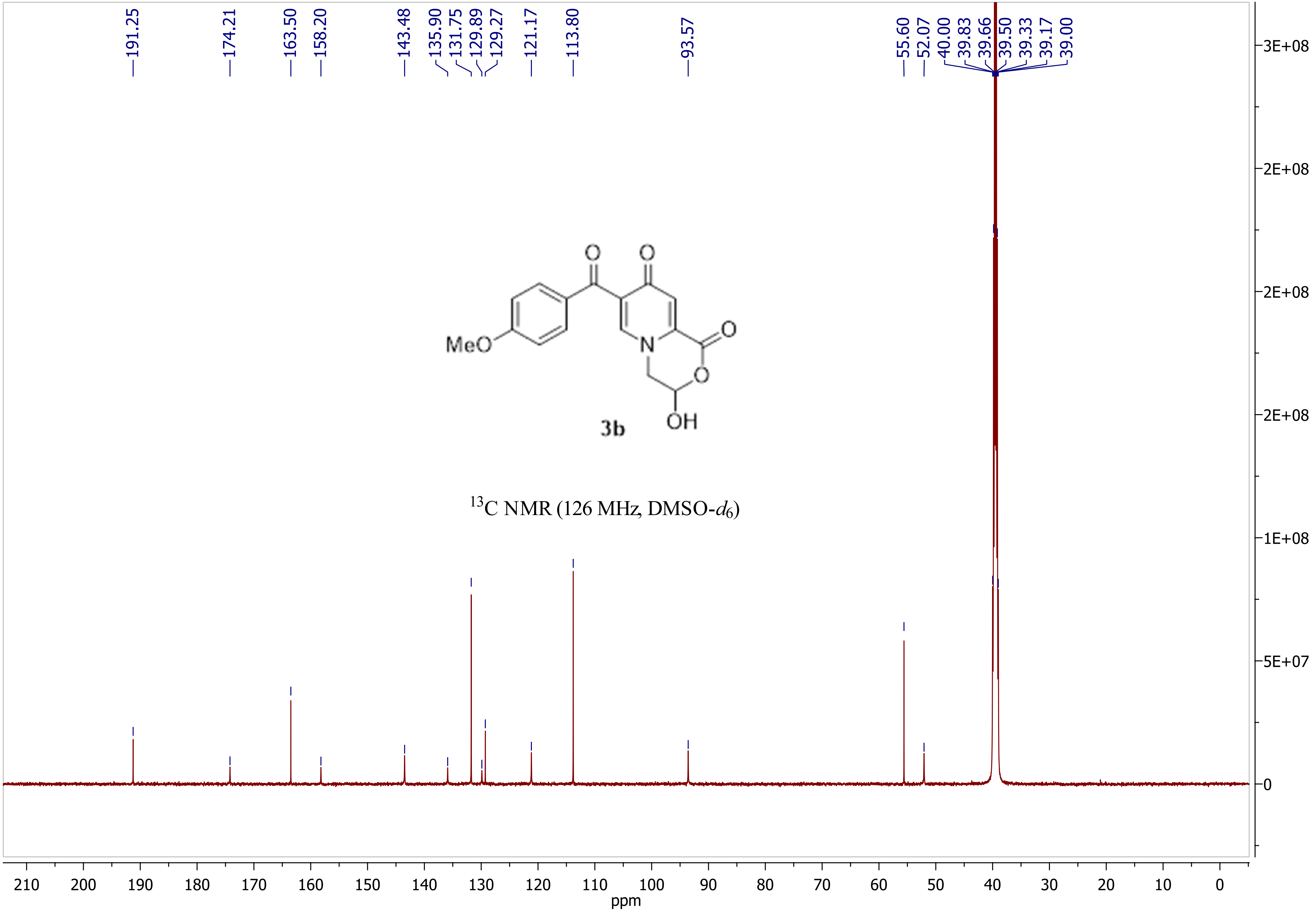


$^{13}$C NMR (126 MHz, DMSO-$d_6$)

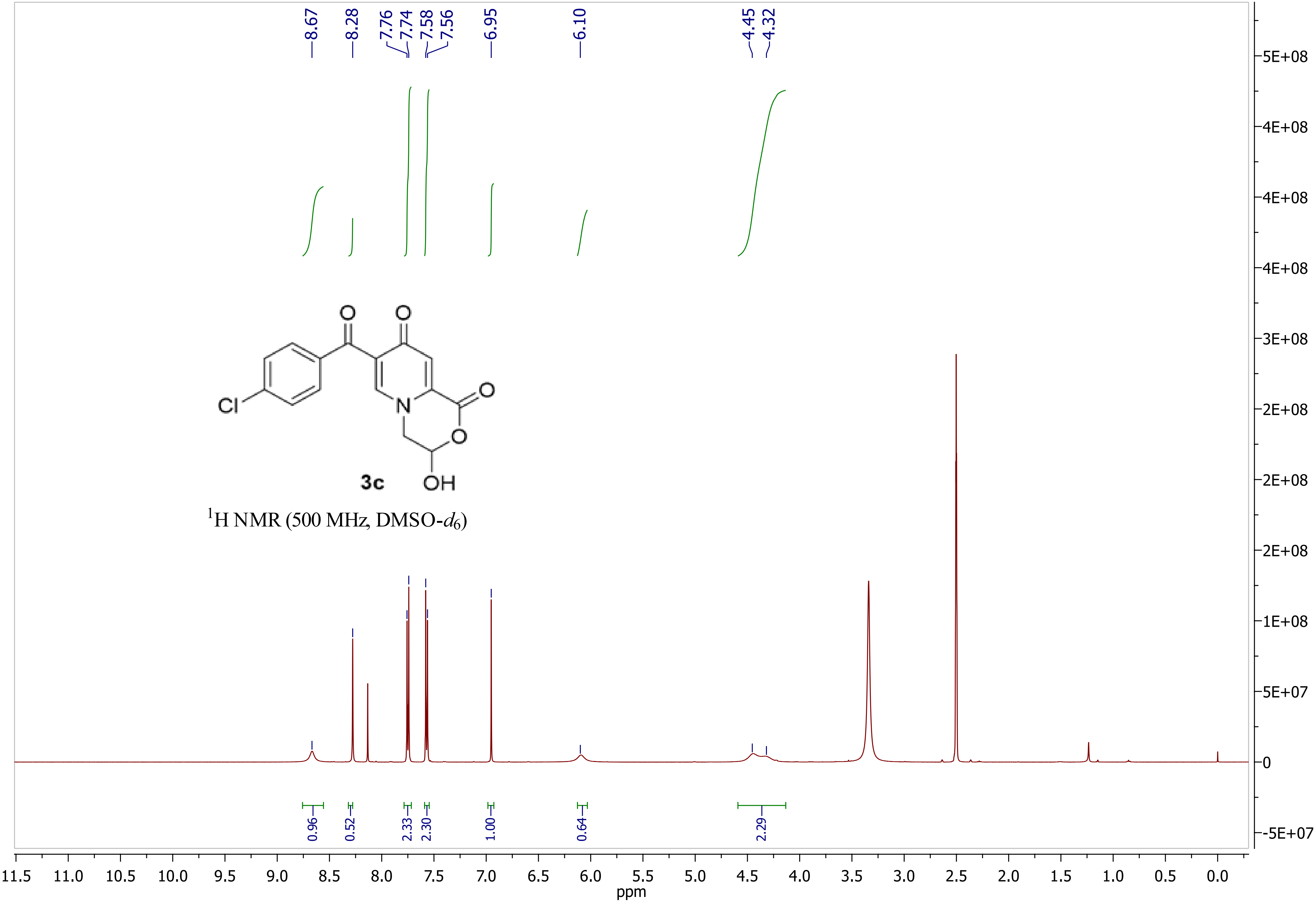
8.67
8.28
7.76
7.74
7.58
7.56
6.95
6.10
4.45
4.32
Cl
O
O
O
N
O
OH
3c
1H NMR (500 MHz, DMSO-d6)
0.96
0.52
2.33
2.30
1.00
0.64
2.29
5E+08
4E+08
3E+08
2E+08
1E+08
5E+07
0
-5E+07
11.5
11.0
10.5
10.0
9.5
9.0
8.5
8.0
7.5
7.0
6.5
6.0
5.5
5.0
4.5
4.0
3.5
3.0
2.5
2.0
1.5
1.0
0.5
0.0
ppm

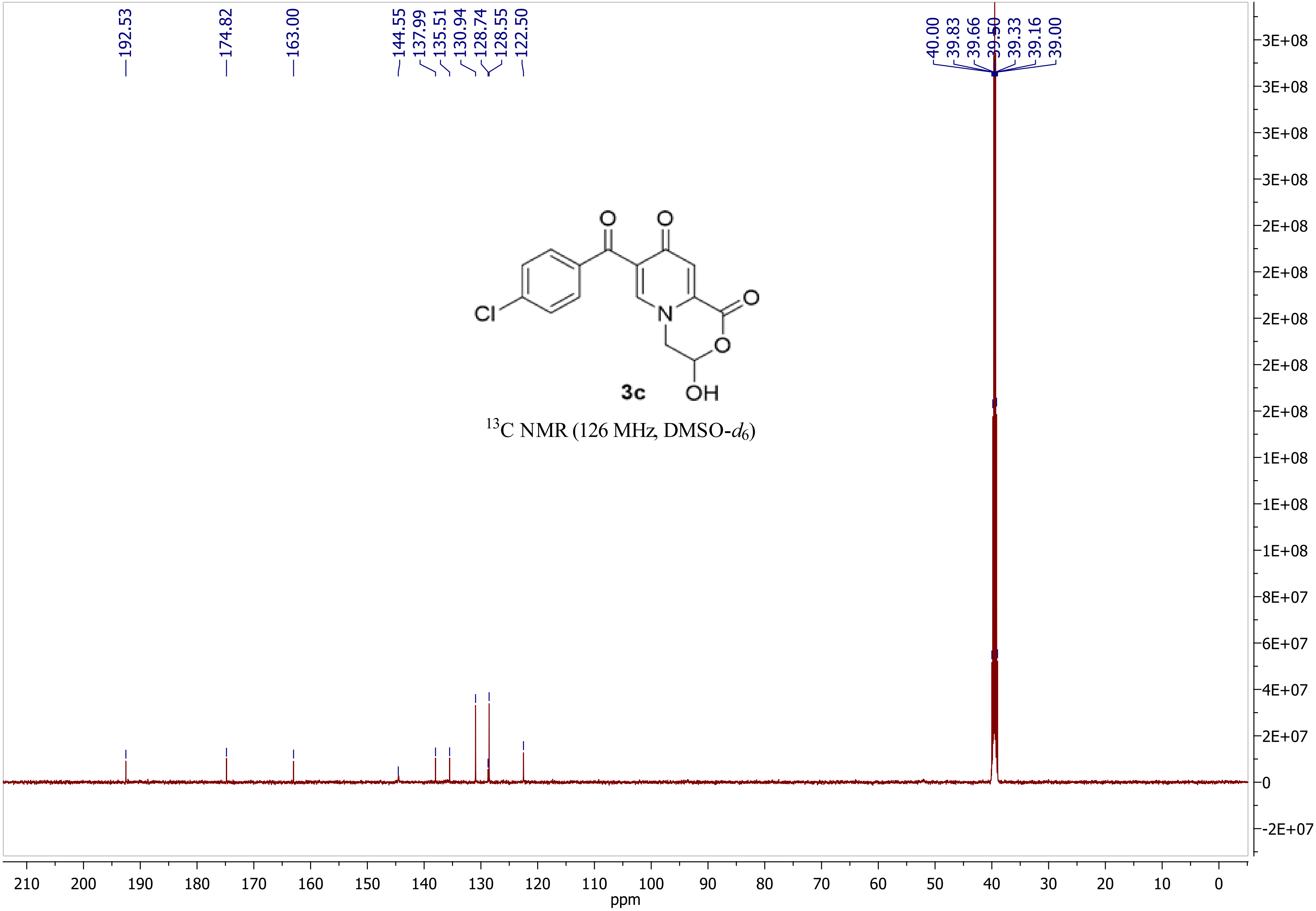
192.53
174.82
163.00
144.55
137.99
135.51
130.94
128.74
128.55
122.50
40.00
39.83
39.66
39.50
39.33
39.16
39.00
3c
13C NMR (126 MHz, DMSO-d6)
ppm

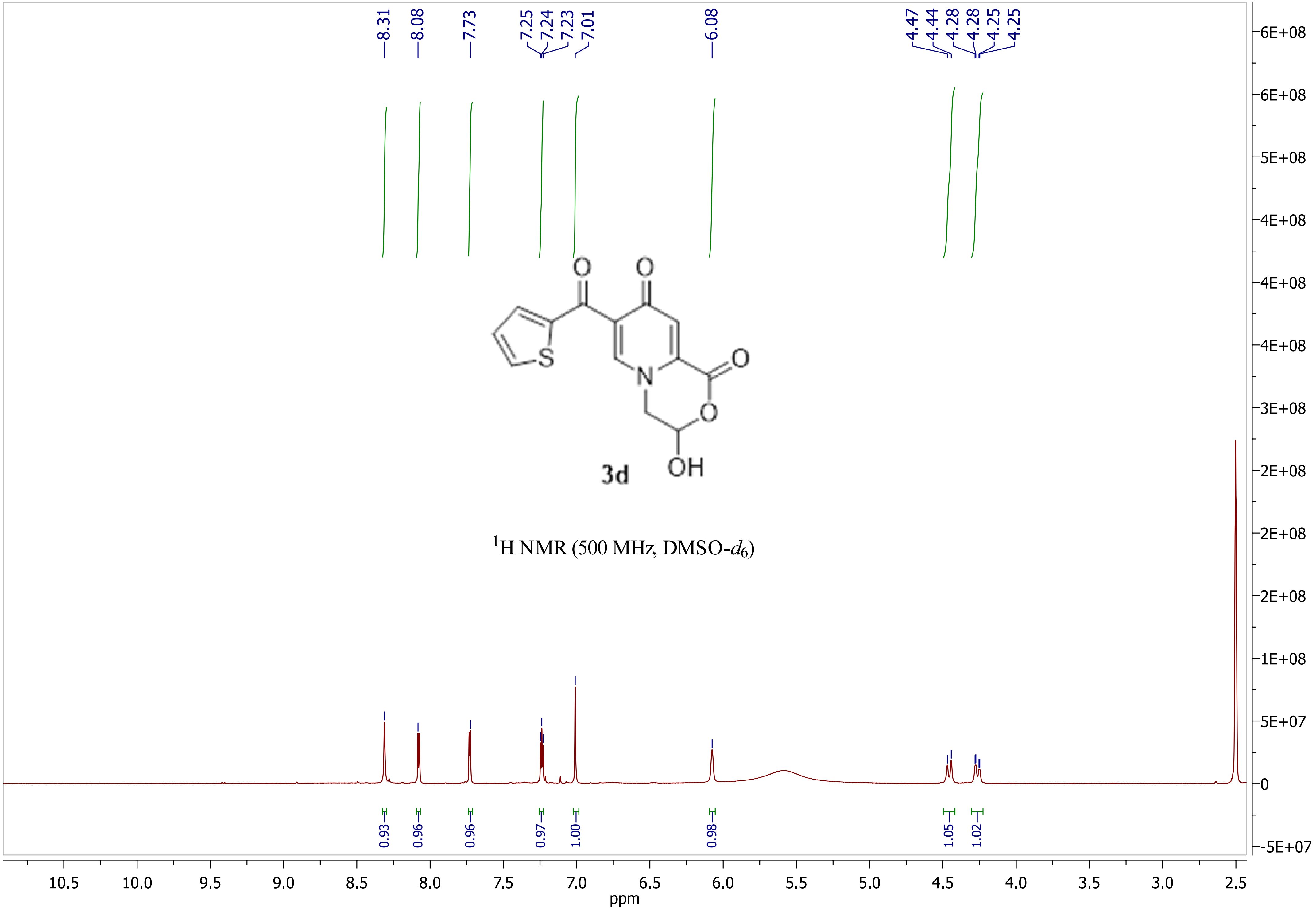
8.31
8.08
7.73
7.25
7.24
7.23
7.01
6.08
4.47
4.44
4.28
4.28
4.25
4.25
3d
OH
$^{1}$H NMR (500 MHz, DMSO-$d_6$)
0.93
0.96
0.96
0.97
1.00
0.98
1.05
1.02
10.5
10.0
9.5
9.0
8.5
8.0
7.5
7.0
6.5
6.0
5.5
5.0
4.5
4.0
3.5
3.0
2.5
ppm
6E+08
6E+08
5E+08
4E+08
4E+08
4E+08
3E+08
2E+08
2E+08
2E+08
1E+08
5E+07
0
-5E+07

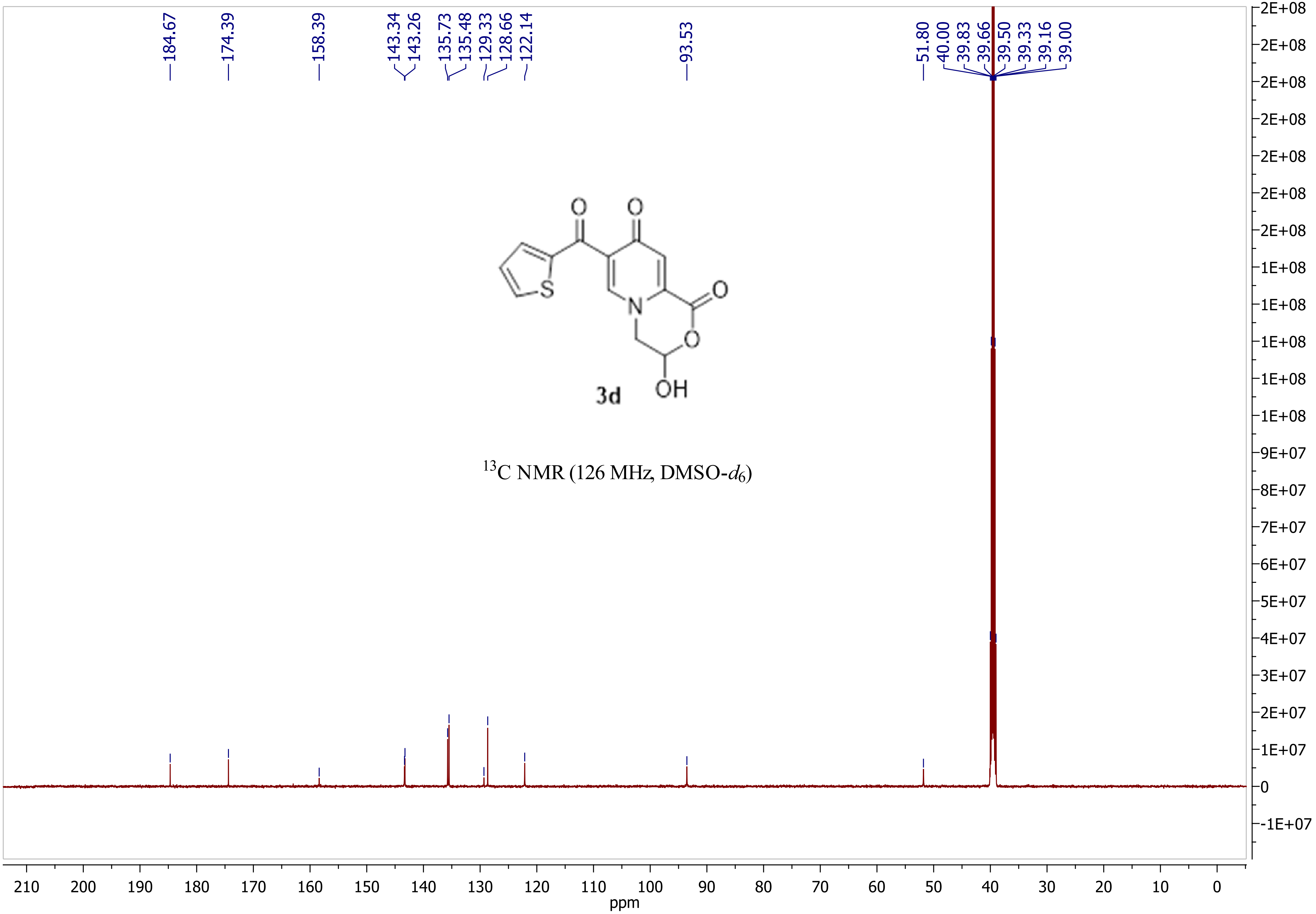
184.67
174.39
158.39
143.34
143.26
135.73
135.48
129.33
128.66
122.14
93.53
51.80
40.00
39.83
39.66
39.50
39.33
39.16
39.00
O
O
O
S
N
O
OH
3d
13C NMR (126 MHz, DMSO-d6)
ppm
210 200 190 180 170 160 150 140 130 120 110 100 90 80 70 60 50 40 30 20 10 0
2E+08
1E+08
9E+07
8E+07
7E+07
6E+07
5E+07
4E+07
3E+07
2E+07
1E+07
0
-1E+07

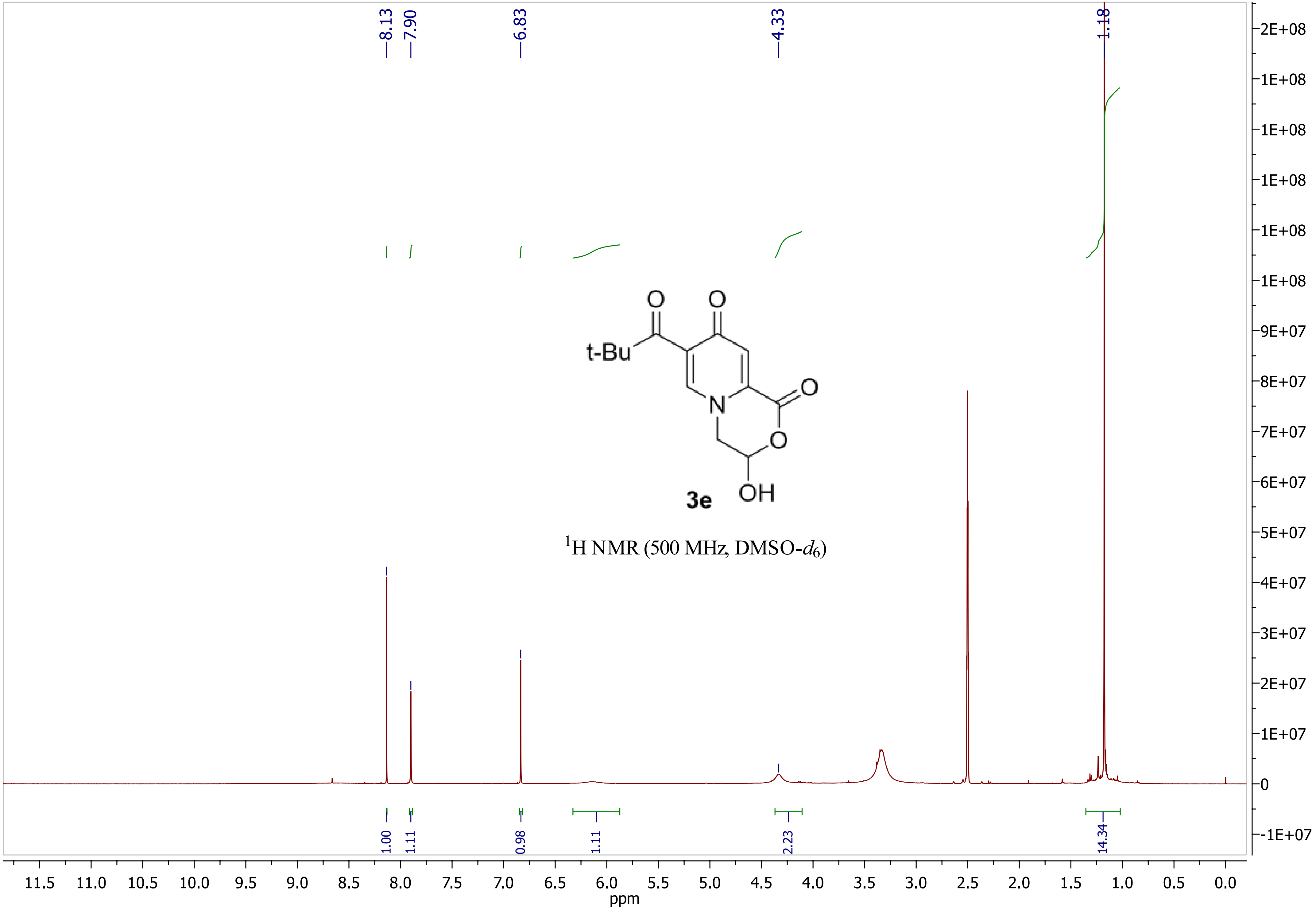

8.13
7.90
6.83
4.33
1.18
O
O
t-Bu
O
N
O
OH
3e
$^{1}$H NMR (500 MHz, DMSO-$d_6$)
1.00
1.11
0.98
1.11
2.23
14.34
11.5 11.0 10.5 10.0 9.5 9.0 8.5 8.0 7.5 7.0 6.5 6.0 5.5 5.0 4.5 4.0 3.5 3.0 2.5 2.0 1.5 1.0 0.5 0.0
ppm
2E+08
1E+08
1E+08
1E+08
1E+08
1E+08
9E+07
8E+07
7E+07
6E+07
5E+07
4E+07
3E+07
2E+07
1E+07
0
-1E+07

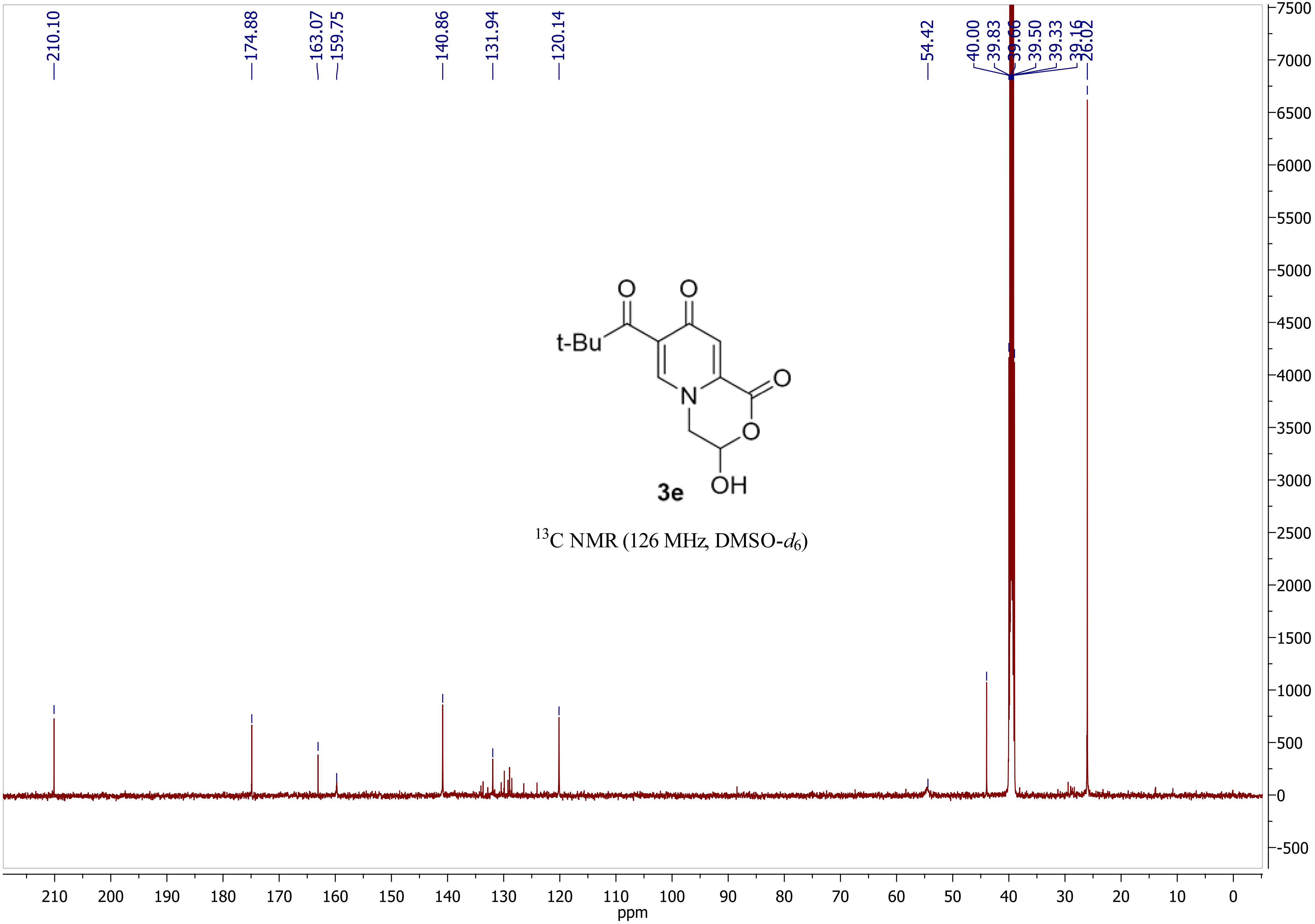


**3e**

$^{13}$C NMR (126 MHz, DMSO-$d_6$)

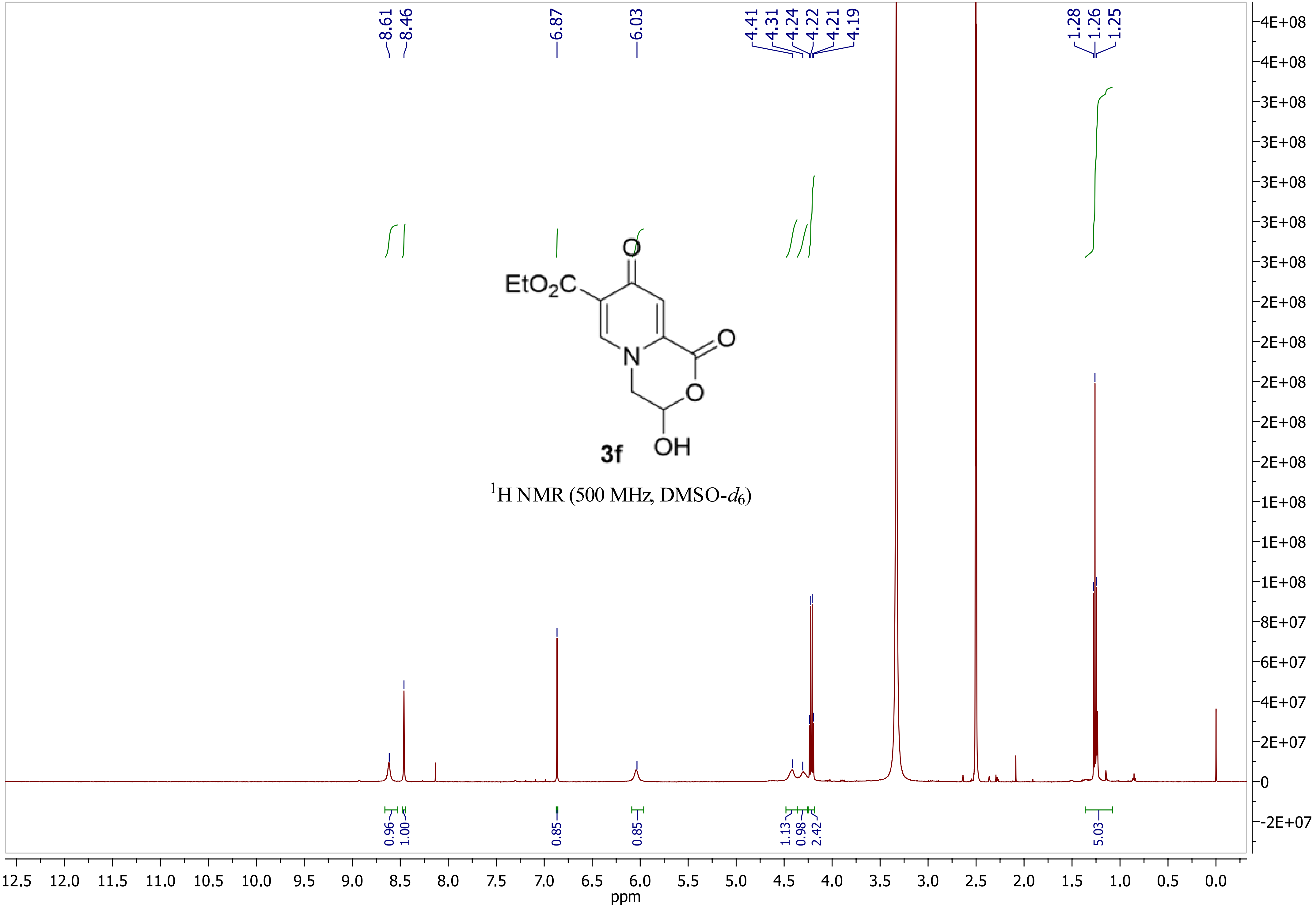
8.61
8.46
6.87
6.03
4.41
4.31
4.24
4.22
4.21
4.19
1.28
1.26
1.25
EtO2C
O
O
N
O
OH
3f
1H NMR (500 MHz, DMSO-d6)
0.96
1.00
0.85
0.85
1.13
0.98
2.42
5.03
ppm

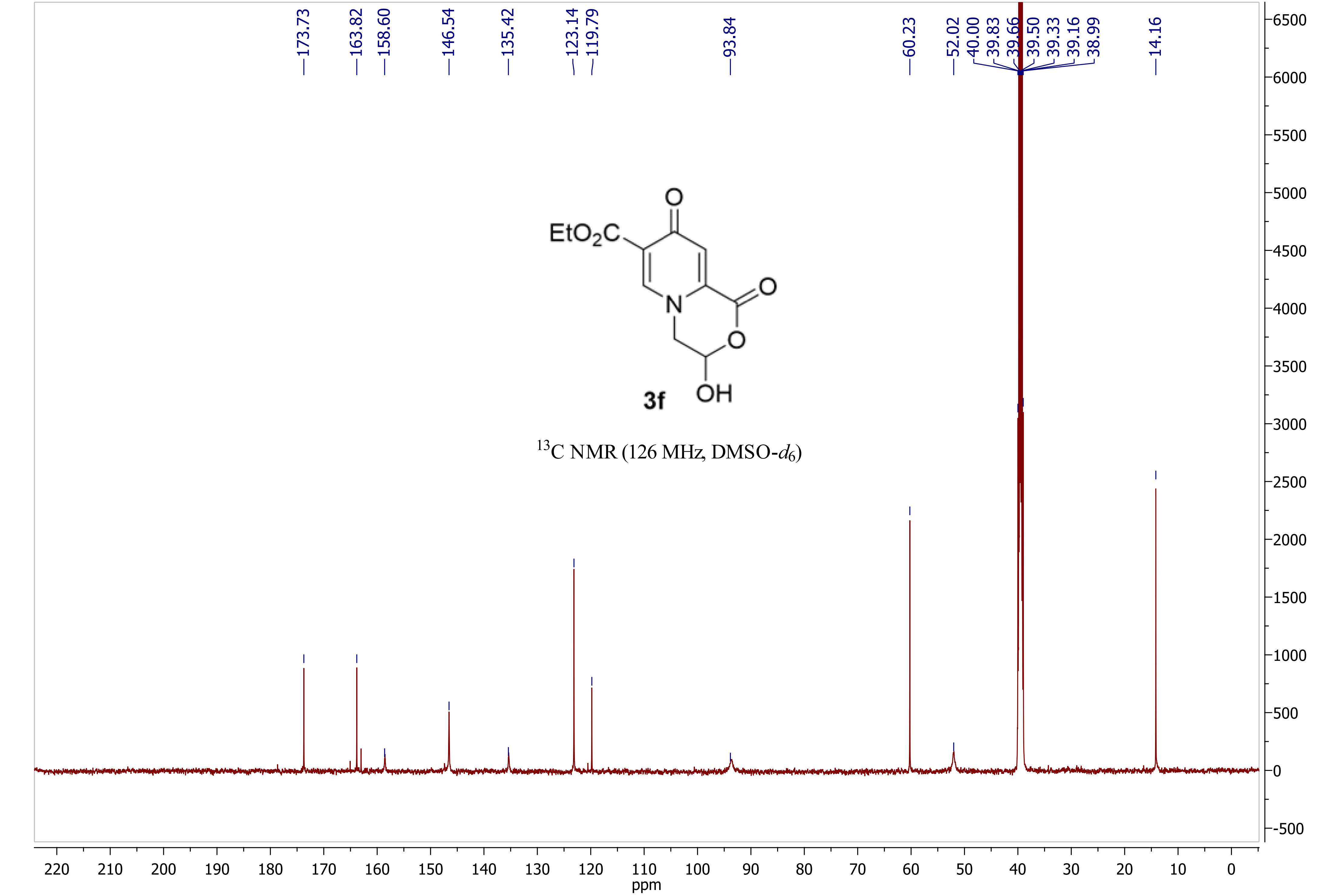
173.73
163.82
158.60
146.54
135.42
123.14
119.79
93.84
60.23
52.02
40.00
39.83
39.66
39.50
39.33
39.16
38.99
14.16
EtO2C
O
N
O
O
OH
3f
13C NMR (126 MHz, DMSO-d6)
ppm
220 210 200 190 180 170 160 150 140 130 120 110 100 90 80 70 60 50 40 30 20 10 0
6500 6000 5500 5000 4500 4000 3500 3000 2500 2000 1500 1000 500 0 -500

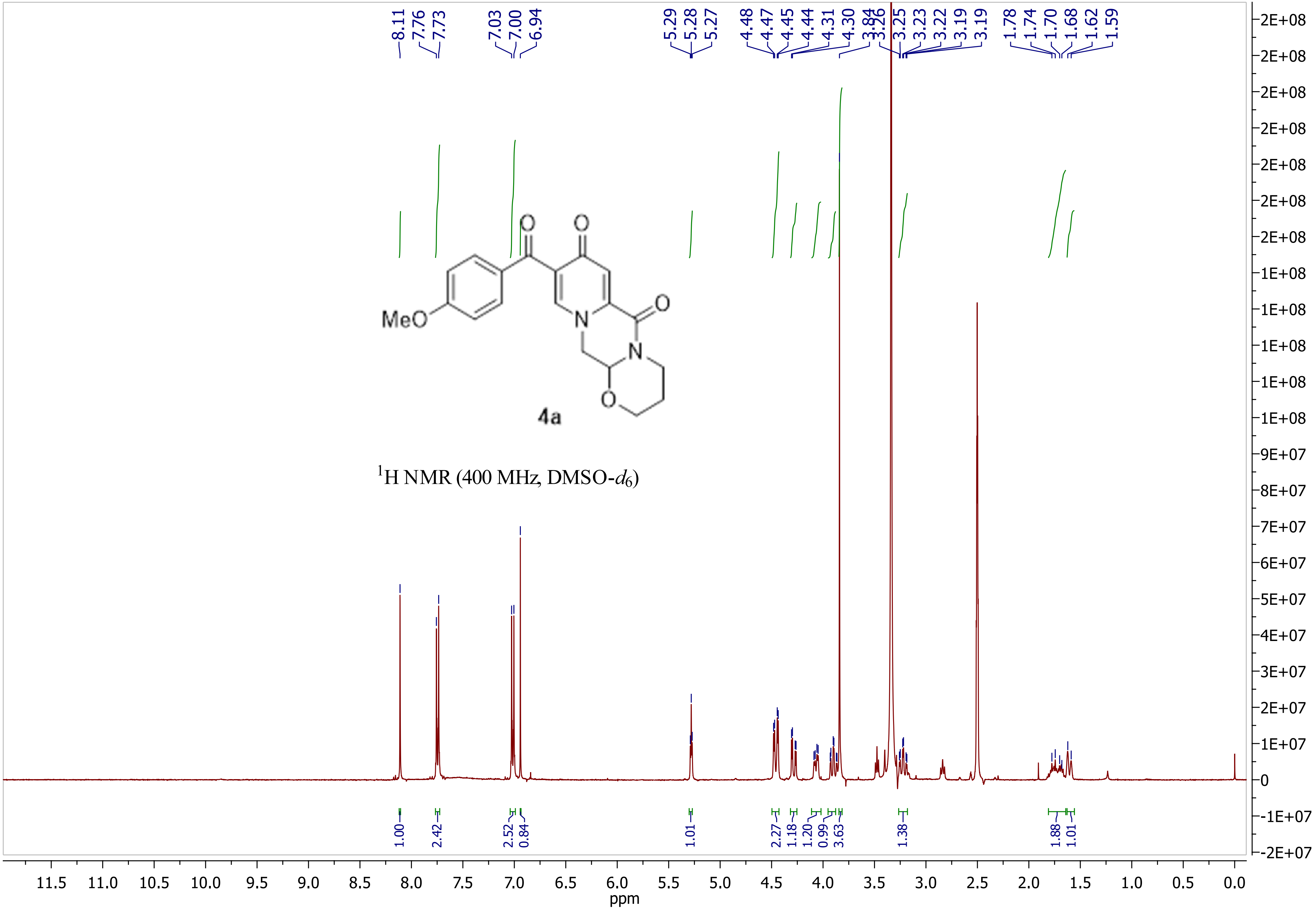
8.11
7.76
7.73
7.03
7.00
6.94
5.29
5.28
5.27
4.48
4.47
4.45
4.44
4.31
4.30
3.84
3.26
3.25
3.23
3.22
3.19
3.19
1.78
1.74
1.70
1.68
1.62
1.59
MeO
O
N
4a
$^1$H NMR (400 MHz, DMSO-$d_6$)
1.00
2.42
2.52
0.84
1.01
2.27
1.18
1.20
0.99
3.63
1.38
1.88
1.01
ppm

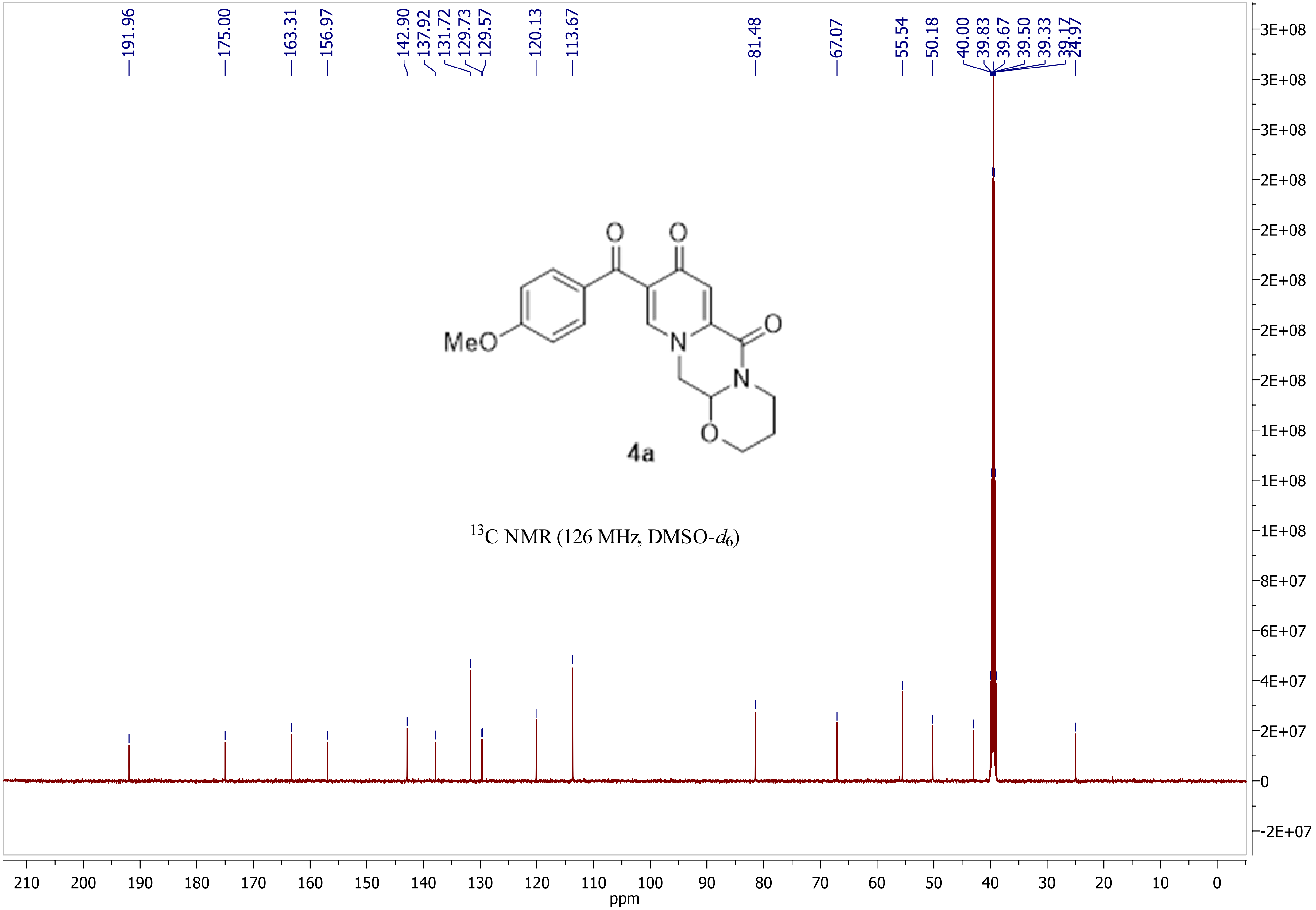


$^{13}C$ NMR (126 MHz, DMSO-$d_6$)

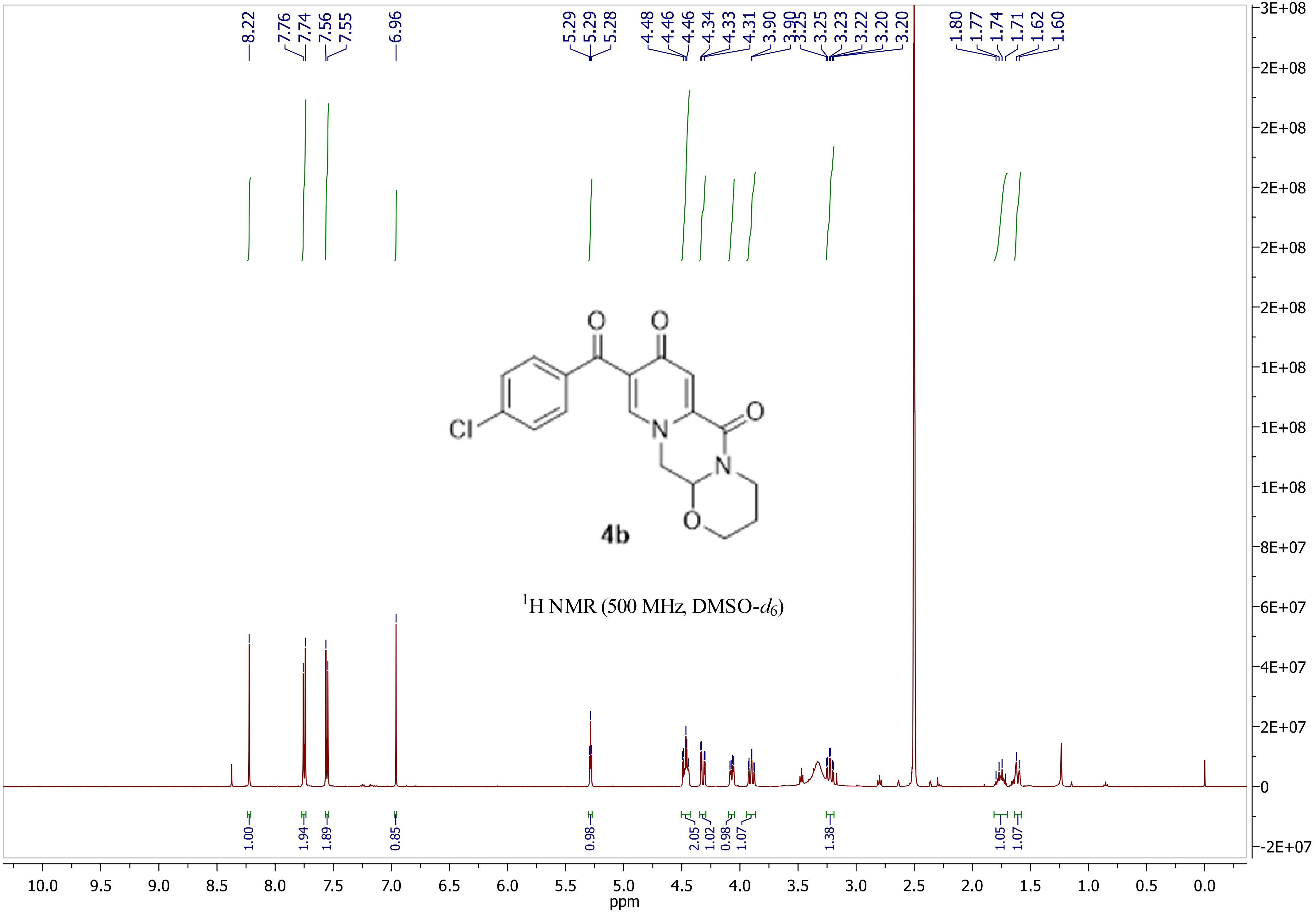
8.22
7.76
7.74
7.56
7.55
6.96
5.29
5.29
5.28
4.48
4.46
4.46
4.34
4.33
4.31
3.90
3.90
3.25
3.25
3.23
3.22
3.20
3.20
1.80
1.77
1.74
1.71
1.62
1.60
Cl
O
O
O
N
N
O
4b
1H NMR (500 MHz, DMSO-d6)
1.00
1.94
1.89
0.85
0.98
2.05
1.02
0.98
1.07
1.38
1.05
1.07
10.0
9.5
9.0
8.5
8.0
7.5
7.0
6.5
6.0
5.5
5.0
4.5
4.0
3.5
3.0
2.5
2.0
1.5
1.0
0.5
0.0
ppm
3E+08
2E+08
2E+08
2E+08
2E+08
2E+08
1E+08
1E+08
1E+08
8E+07
6E+07
4E+07
2E+07
0
-2E+07

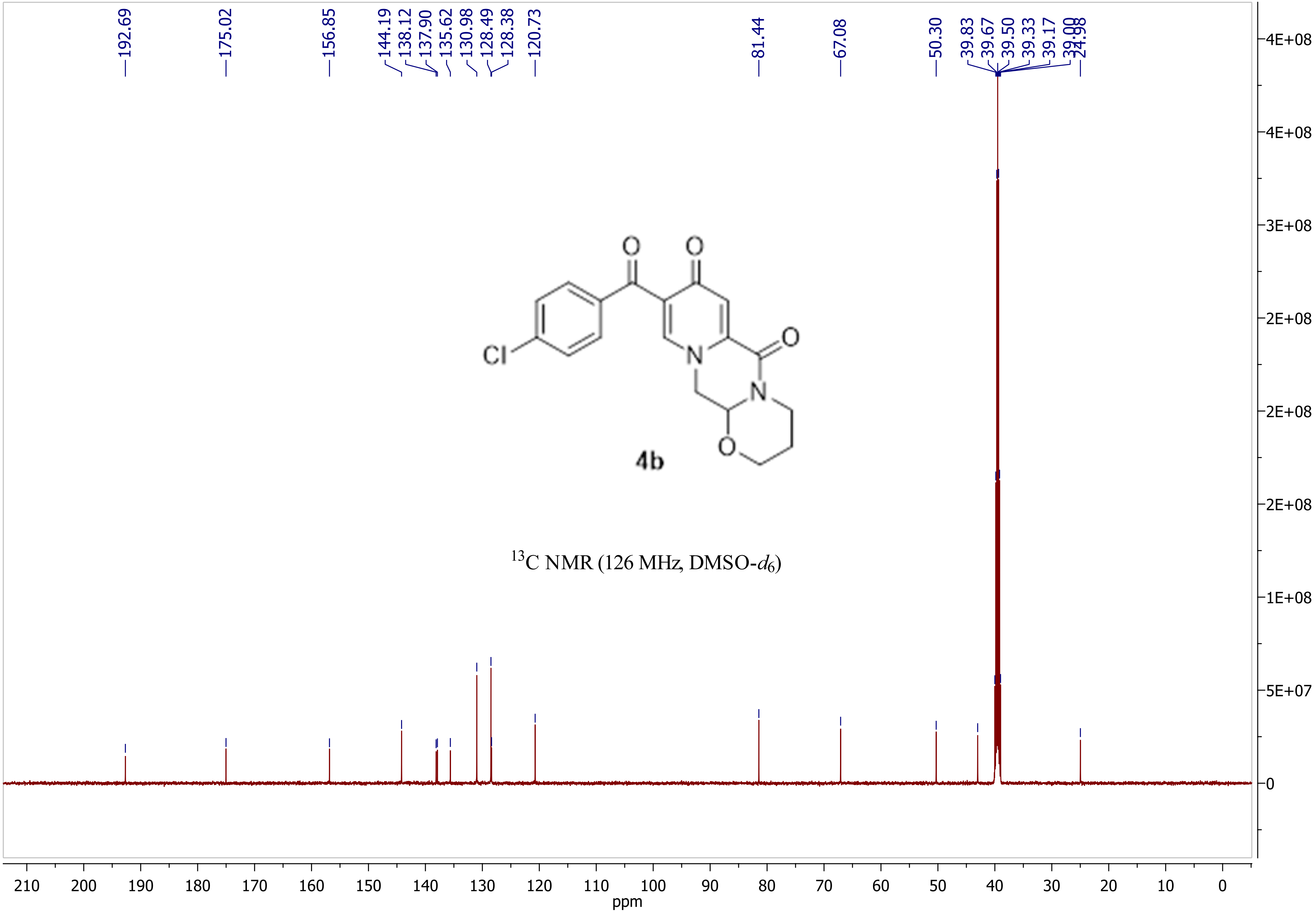
192.69
175.02
156.85
144.19
138.12
137.90
135.62
130.98
128.49
128.38
120.73
81.44
67.08
50.30
39.83
39.67
39.50
39.33
39.17
39.00
24.98
Cl
O
N
4b
$^{13}$C NMR (126 MHz, DMSO-$d_6$)
ppm

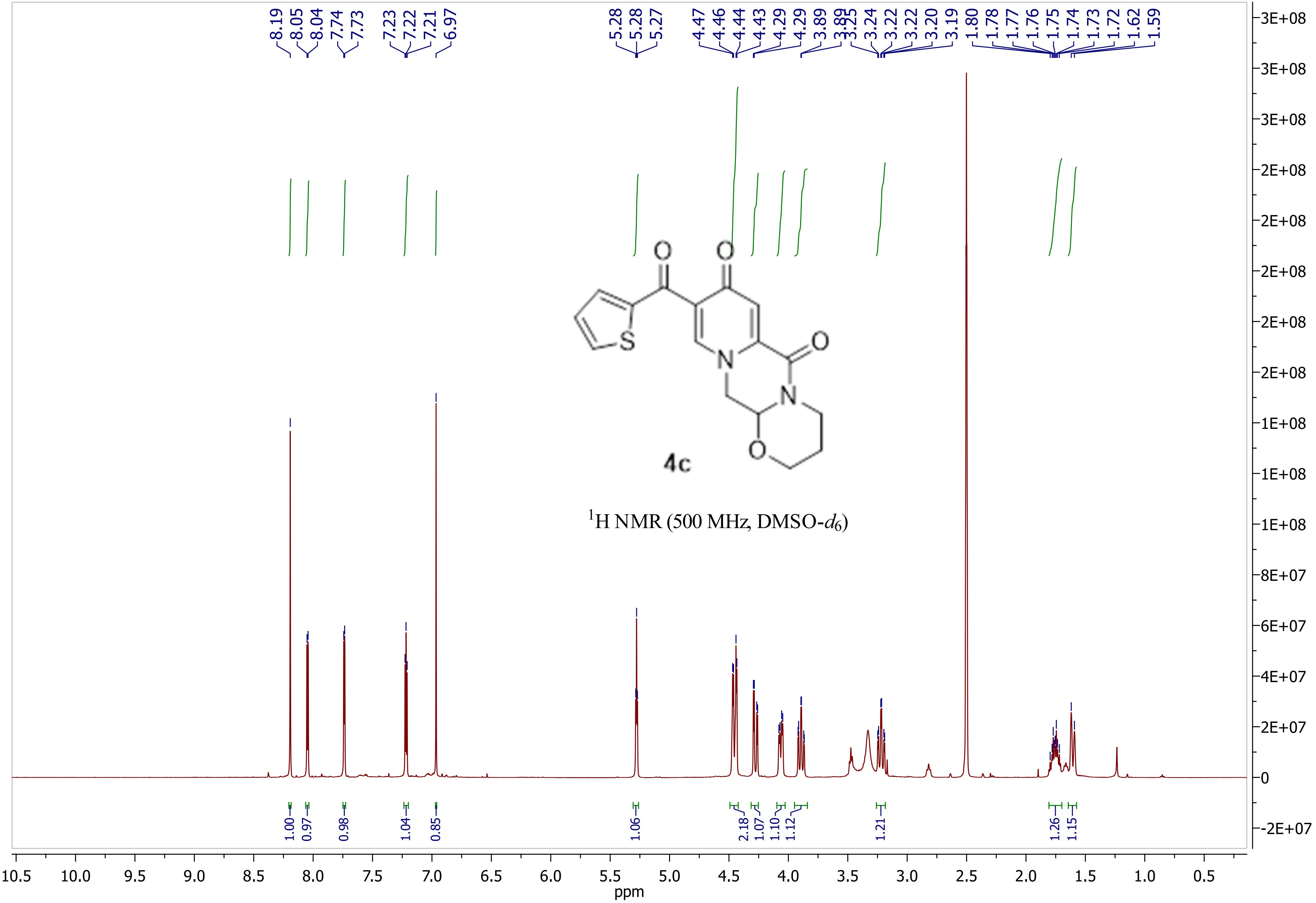
8.19
8.05
8.04
7.74
7.73
7.23
7.22
7.21
6.97
5.28
5.28
5.27
4.47
4.46
4.44
4.43
4.29
4.29
3.89
3.89
3.25
3.24
3.22
3.22
3.20
3.19
1.80
1.78
1.77
1.76
1.75
1.74
1.73
1.72
1.62
1.59
4c
$^1$H NMR (500 MHz, DMSO-$d_6$)
1.00
0.97
0.98
1.04
0.85
1.06
2.18
1.07
1.10
1.12
1.21
1.26
1.15
ppm

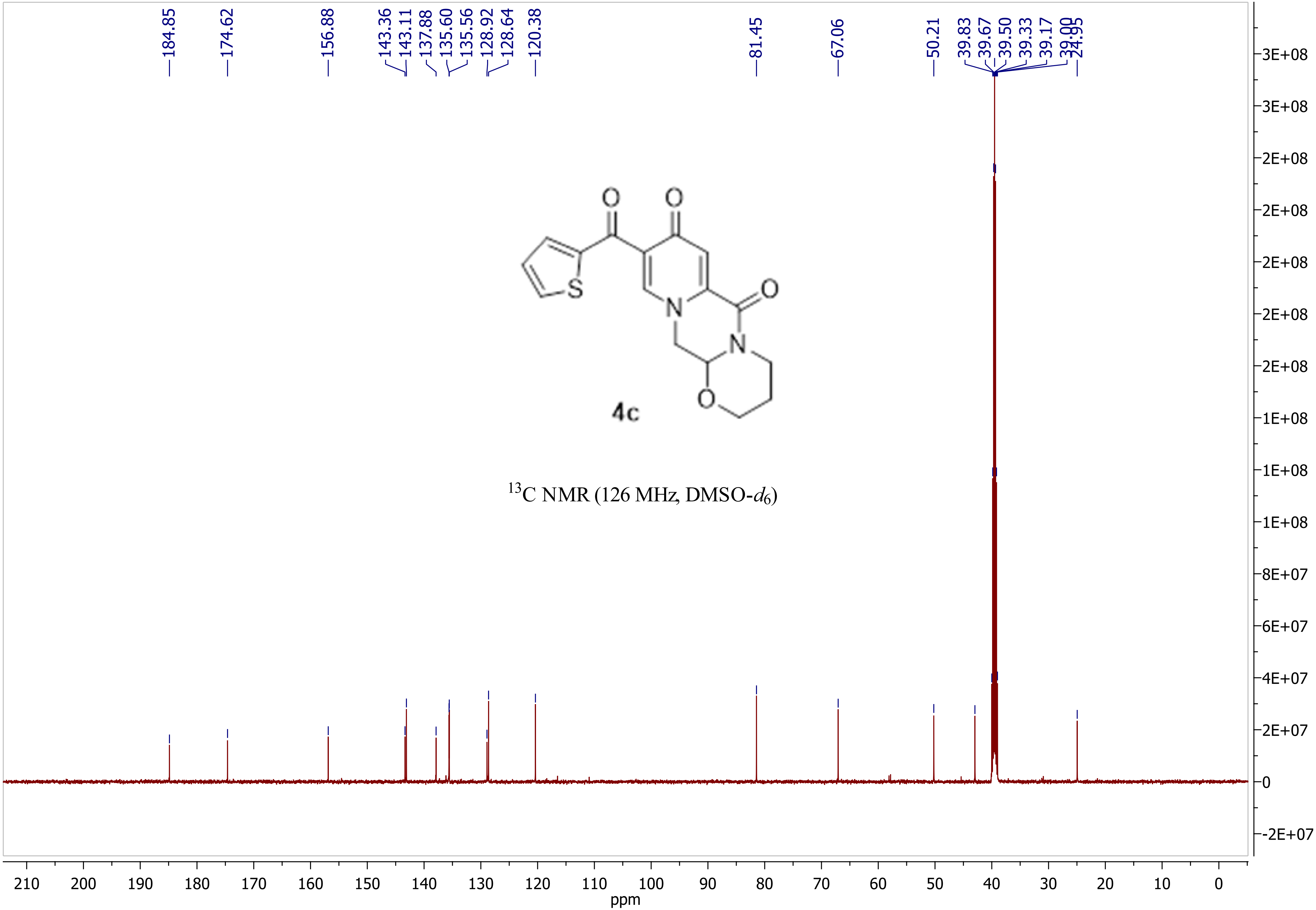

184.85
174.62
156.88
143.36
143.11
137.88
135.60
135.56
128.92
128.64
120.38
81.45
67.06
50.21
39.83
39.67
39.50
39.33
39.17
39.00
24.95
4c
13C NMR (126 MHz, DMSO-d6)
3E+08
3E+08
2E+08
2E+08
2E+08
2E+08
2E+08
1E+08
1E+08
1E+08
8E+07
6E+07
4E+07
2E+07
0
-2E+07
210
200
190
180
170
160
150
140
130
120
110
100
90
80
70
60
50
40
30
20
10
0
ppm

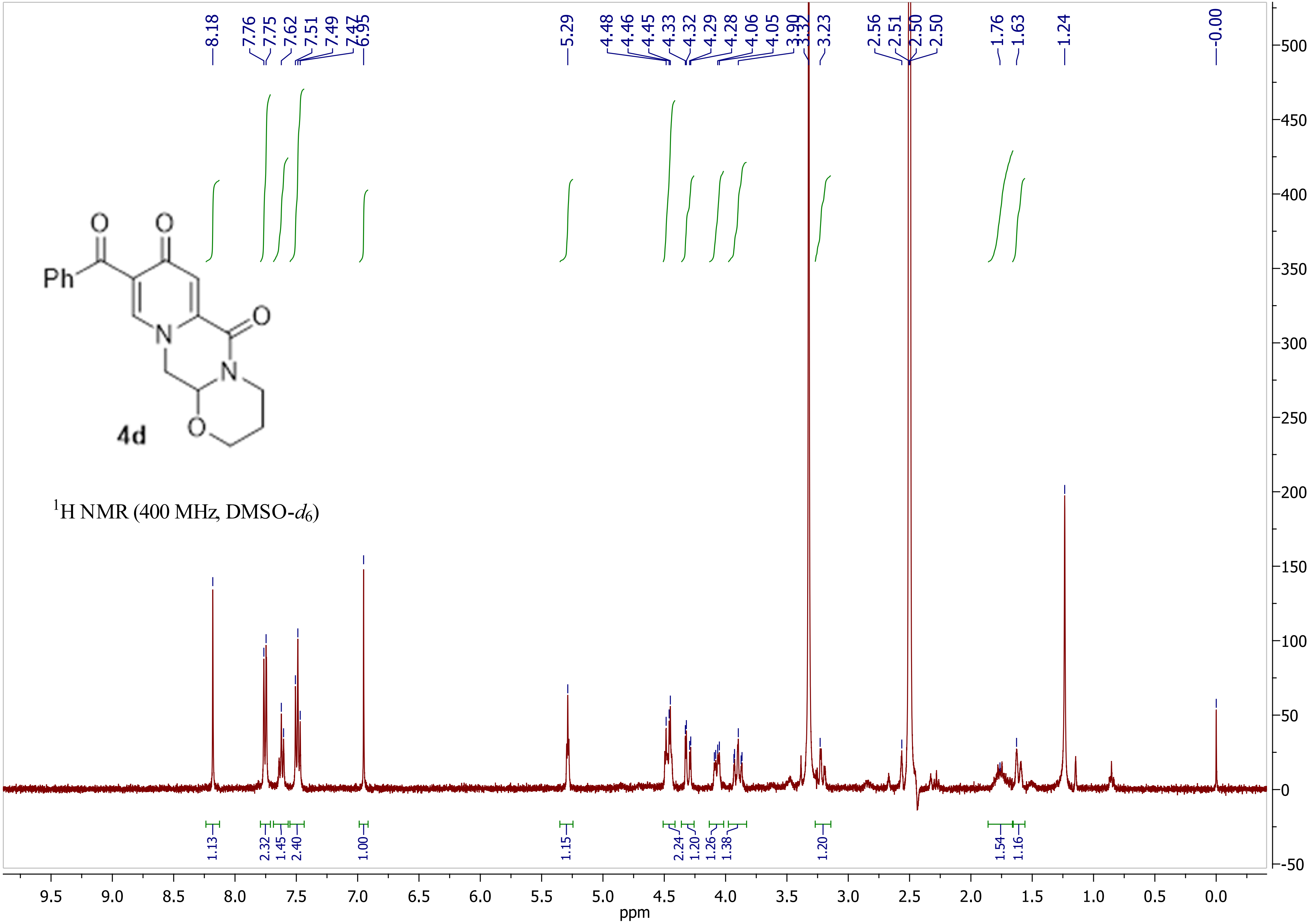


$^{1}$H NMR (400 MHz, DMSO-$d_6$)

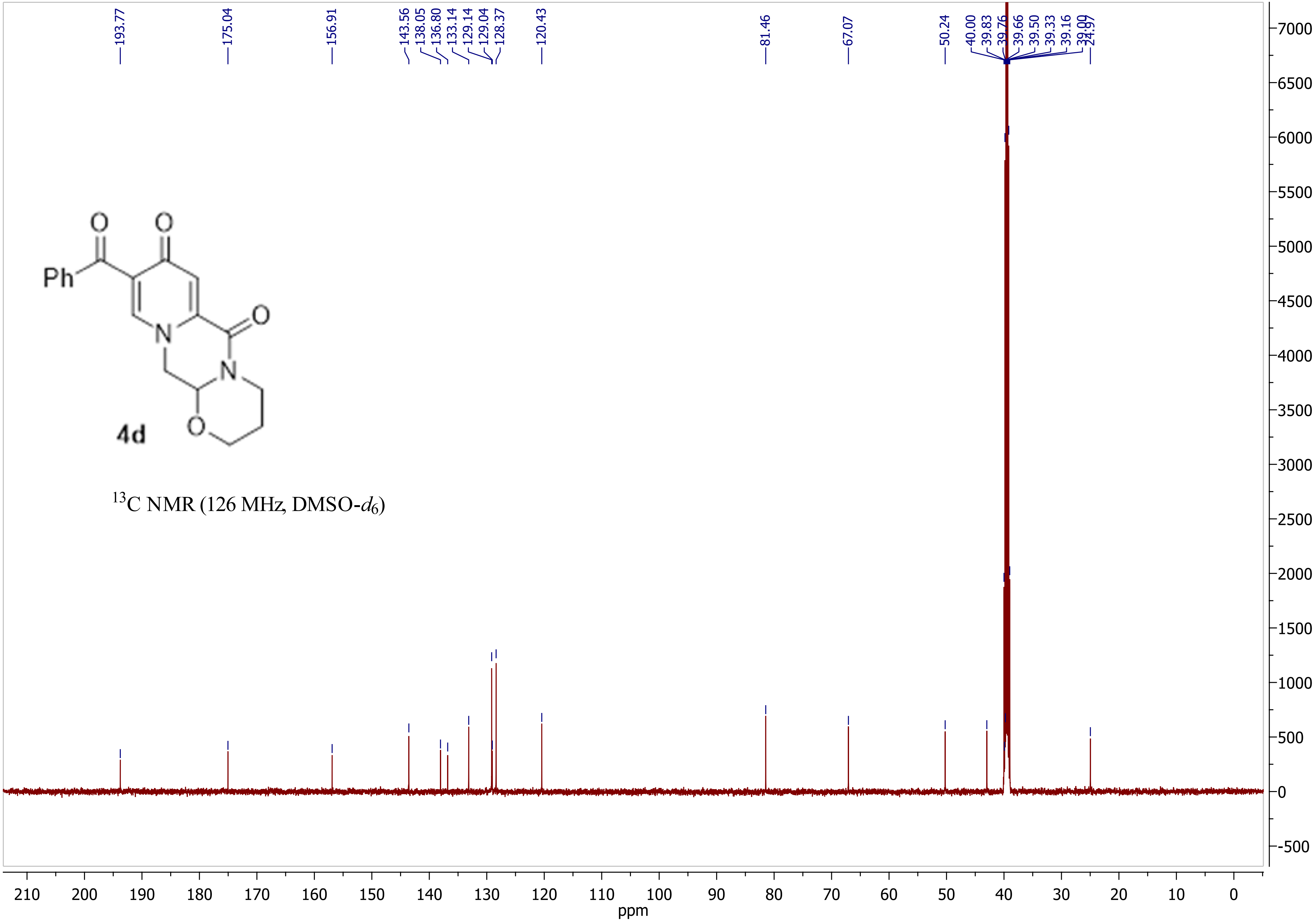
193.77
175.04
156.91
143.56
138.05
136.80
133.14
129.14
129.04
128.37
120.43
81.46
67.07
50.24
40.00
39.83
39.76
39.66
39.50
39.33
39.16
39.00
24.97
Ph
O
N
4d
$^{13}$C NMR (126 MHz, DMSO-$d_6$)
ppm

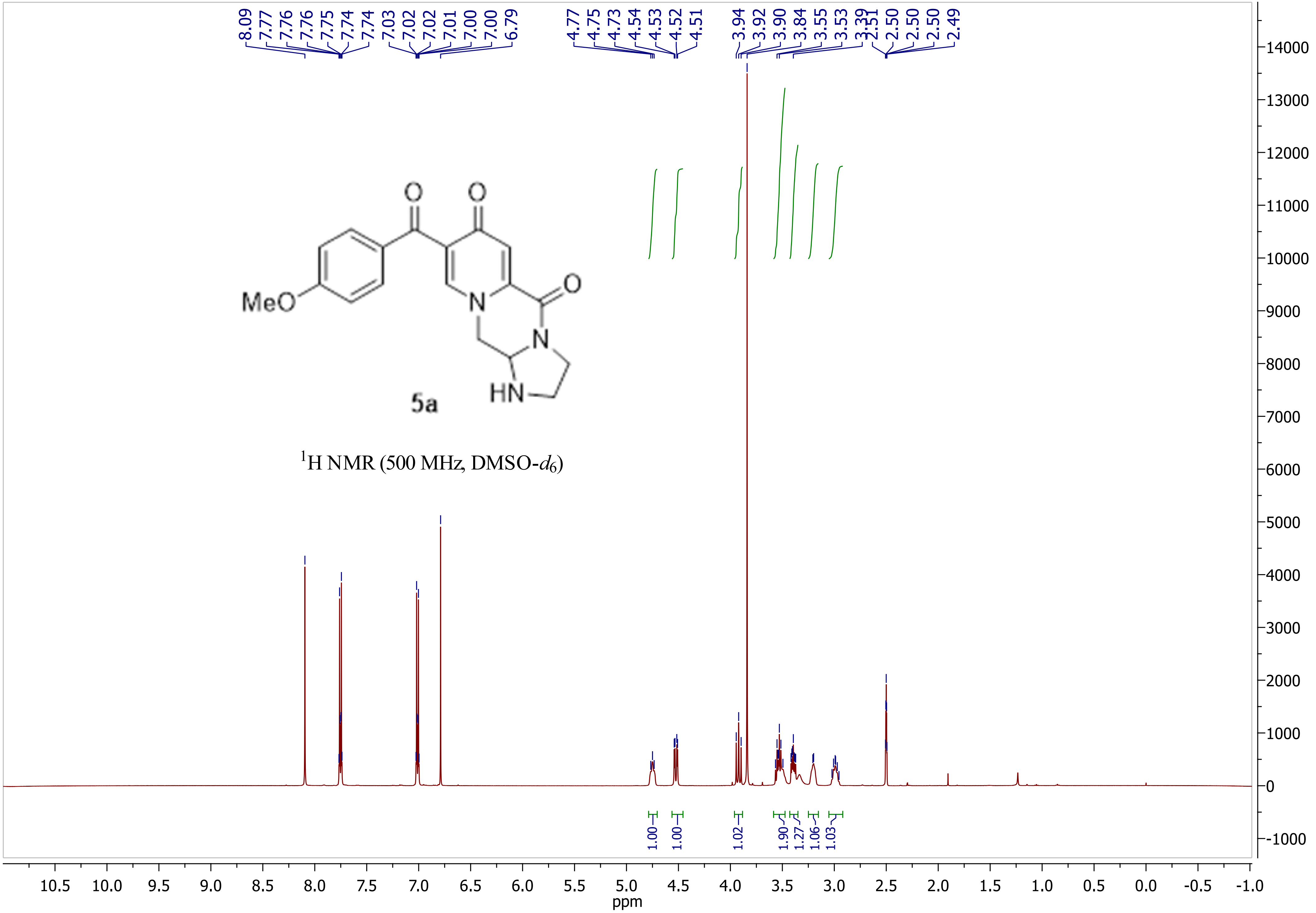
8.09
7.77
7.76
7.76
7.75
7.74
7.74
7.03
7.02
7.02
7.01
7.00
7.00
6.79
4.77
4.75
4.73
4.54
4.53
4.52
4.51
3.94
3.92
3.90
3.84
3.55
3.53
3.39
2.51
2.50
2.50
2.50
2.49
MeO
N
N
HN
5a
$^1$H NMR (500 MHz, DMSO-$d_6$)
1.00
1.00
1.02
1.90
1.27
1.06
1.03
ppm

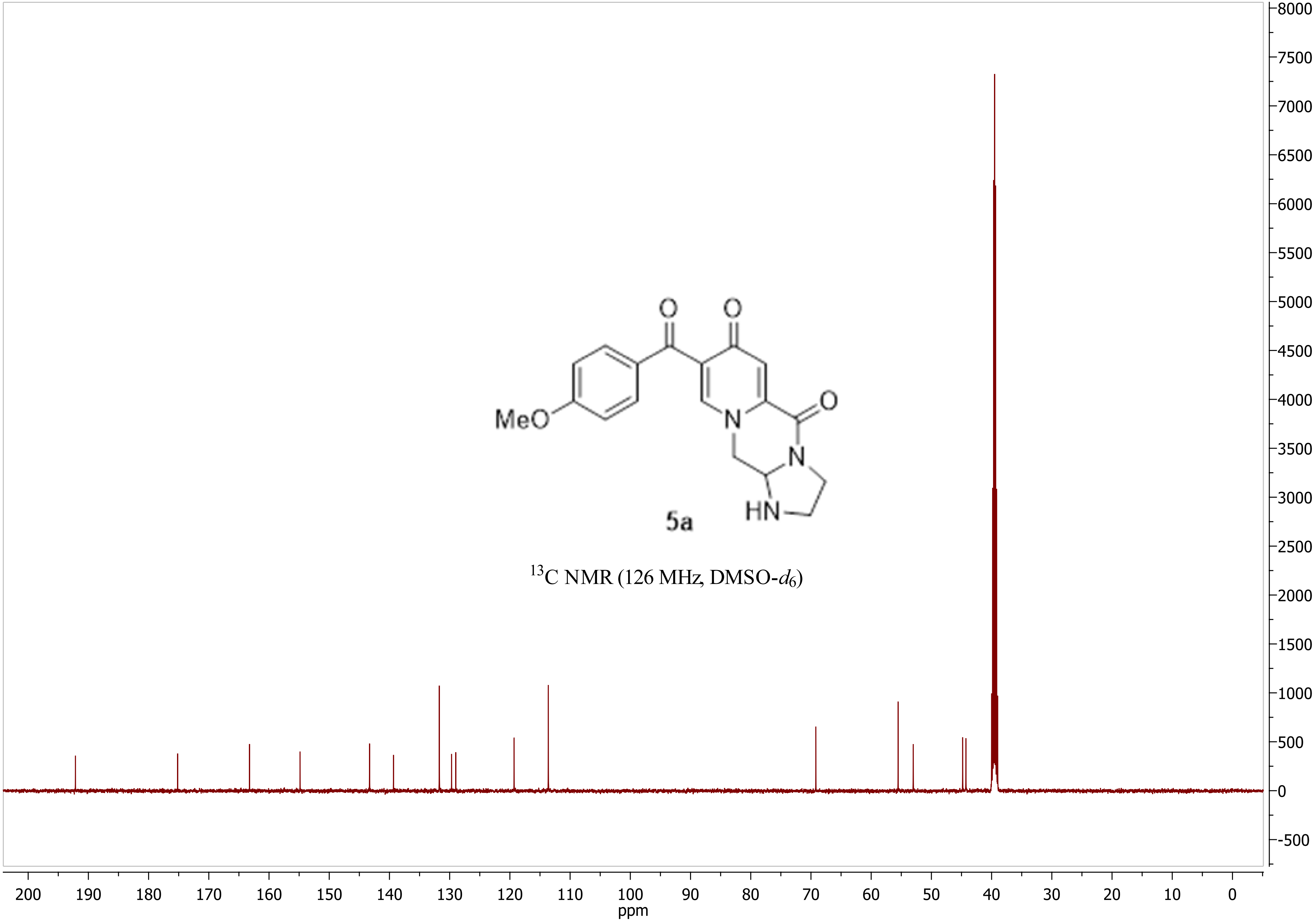
MeO
O
O
O
N
N
HN
5a
13C NMR (126 MHz, DMSO-d6)
8000
7500
7000
6500
6000
5500
5000
4500
4000
3500
3000
2500
2000
1500
1000
500
0
-500
200 190 180 170 160 150 140 130 120 110 100 90 80 70 60 50 40 30 20 10 0
ppm

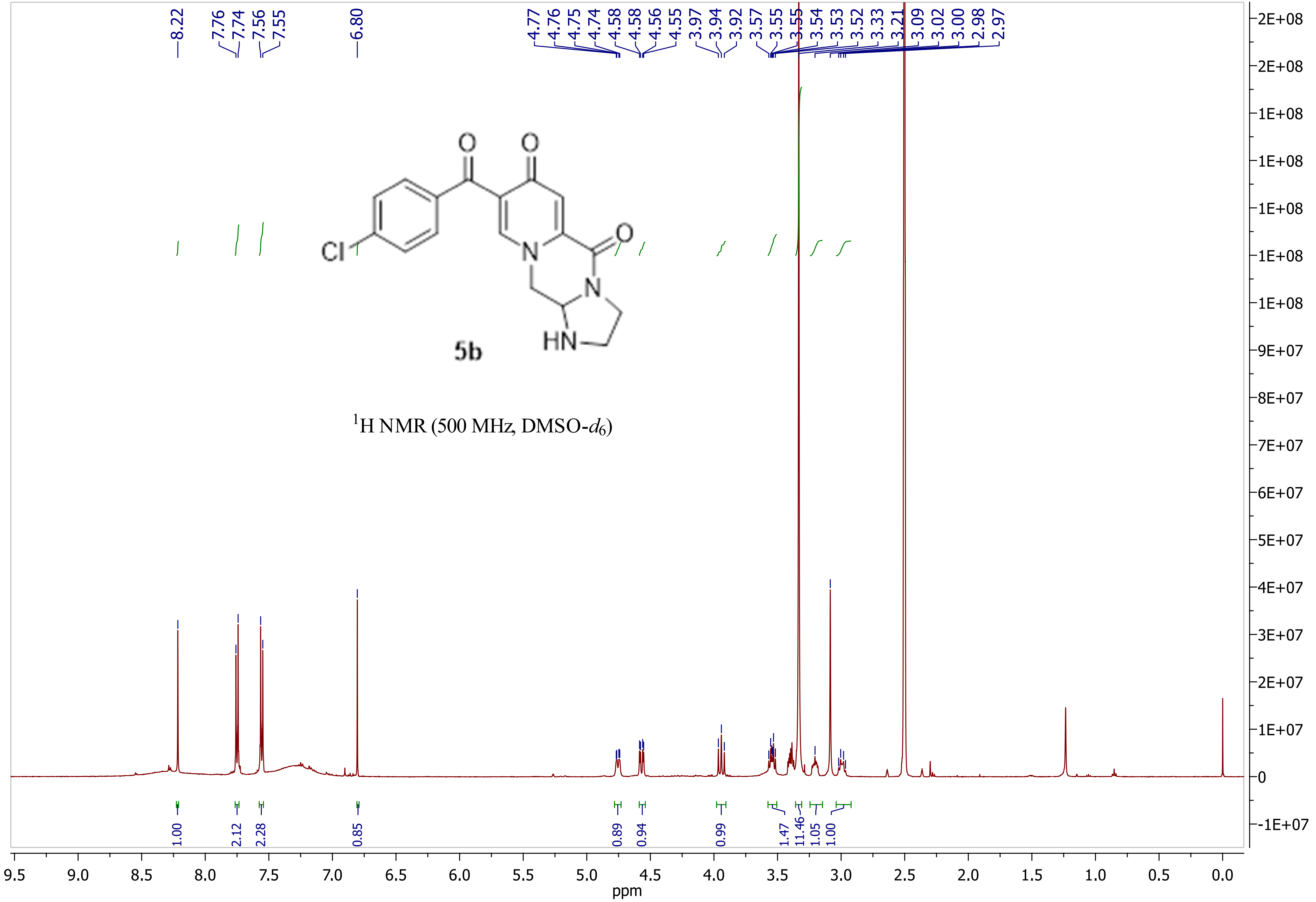
8.22
7.76
7.74
7.56
7.55
6.80
4.77
4.76
4.75
4.74
4.58
4.58
4.56
4.55
3.97
3.94
3.92
3.57
3.55
3.55
3.54
3.53
3.52
3.33
3.21
3.09
3.02
3.00
2.98
2.97
Cl
O
O
O
N
N
HN
5b
1H NMR (500 MHz, DMSO-d6)
1.00
2.12
2.28
0.85
0.89
0.94
0.99
1.47
11.46
1.05
1.00
ppm

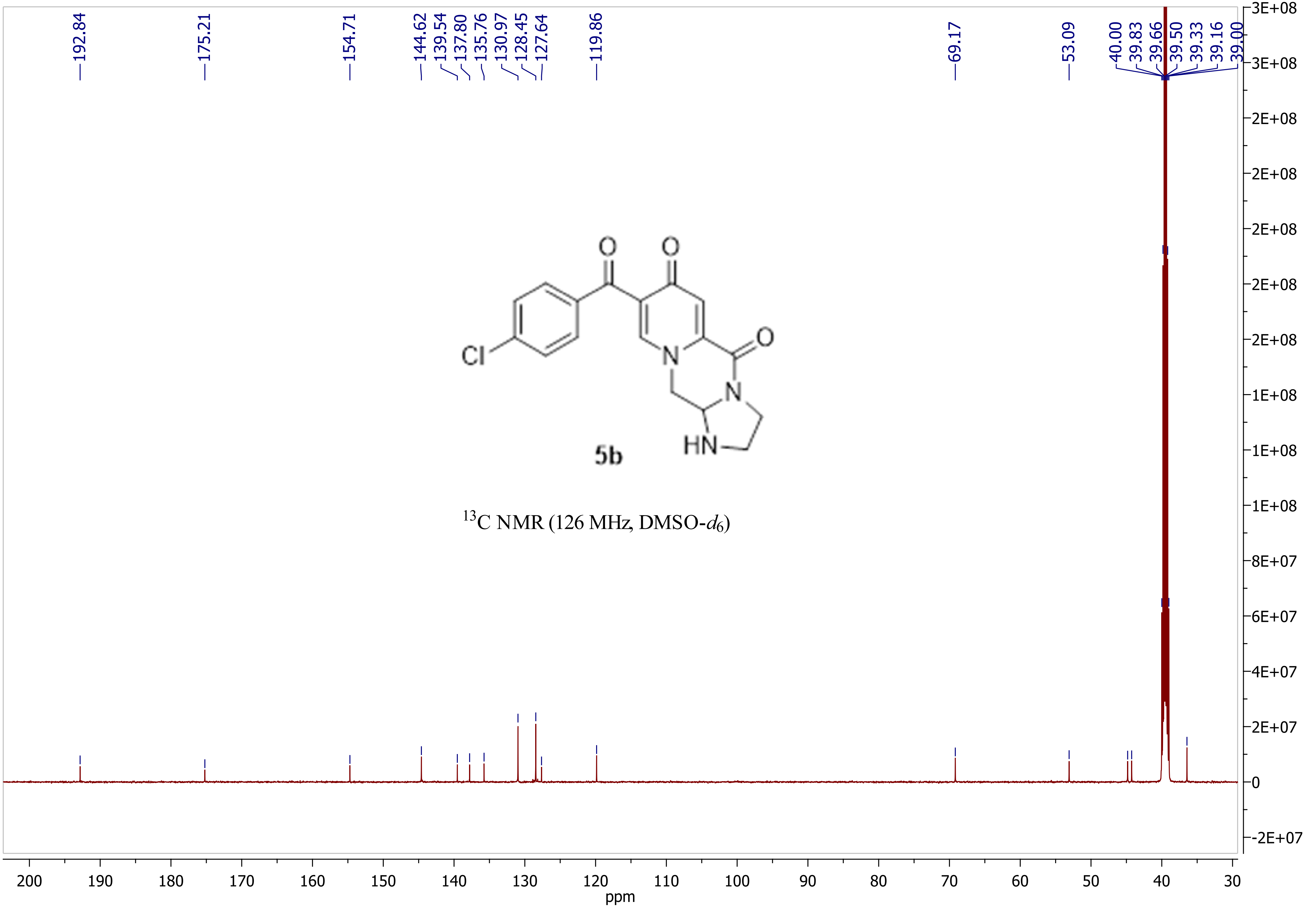


$^{13}C$ NMR (126 MHz, DMSO-$d_6$)

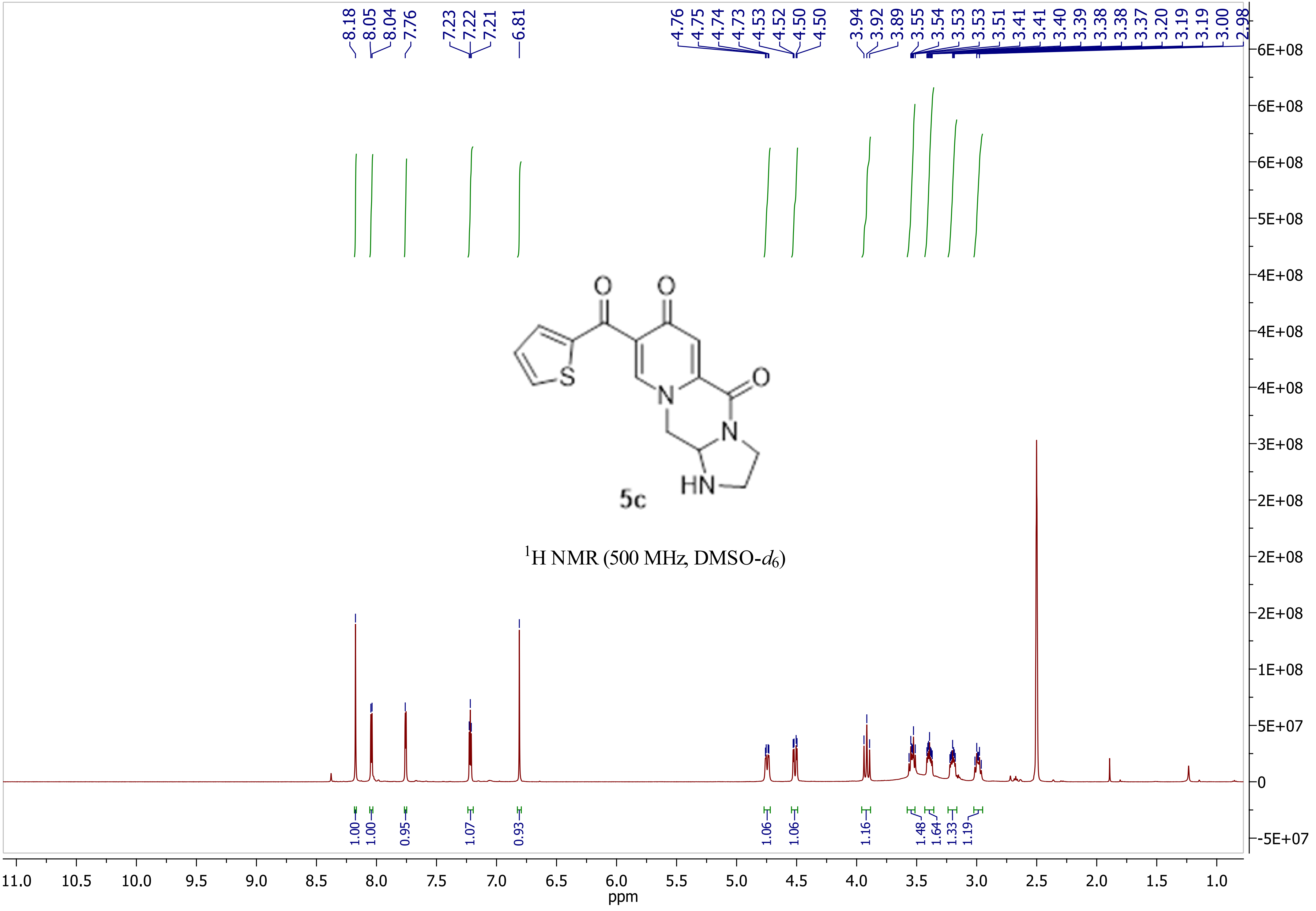
8.18
8.05
8.04
7.76
7.23
7.22
7.21
6.81
4.76
4.75
4.74
4.73
4.53
4.52
4.50
4.50
3.94
3.92
3.89
3.55
3.54
3.53
3.53
3.51
3.41
3.41
3.40
3.39
3.38
3.38
3.37
3.20
3.19
3.19
3.00
2.98
5c
HN
N
N
O
O
O
S
1H NMR (500 MHz, DMSO-d6)
1.00
1.00
0.95
1.07
0.93
1.06
1.06
1.16
1.48
1.64
1.33
1.19
ppm

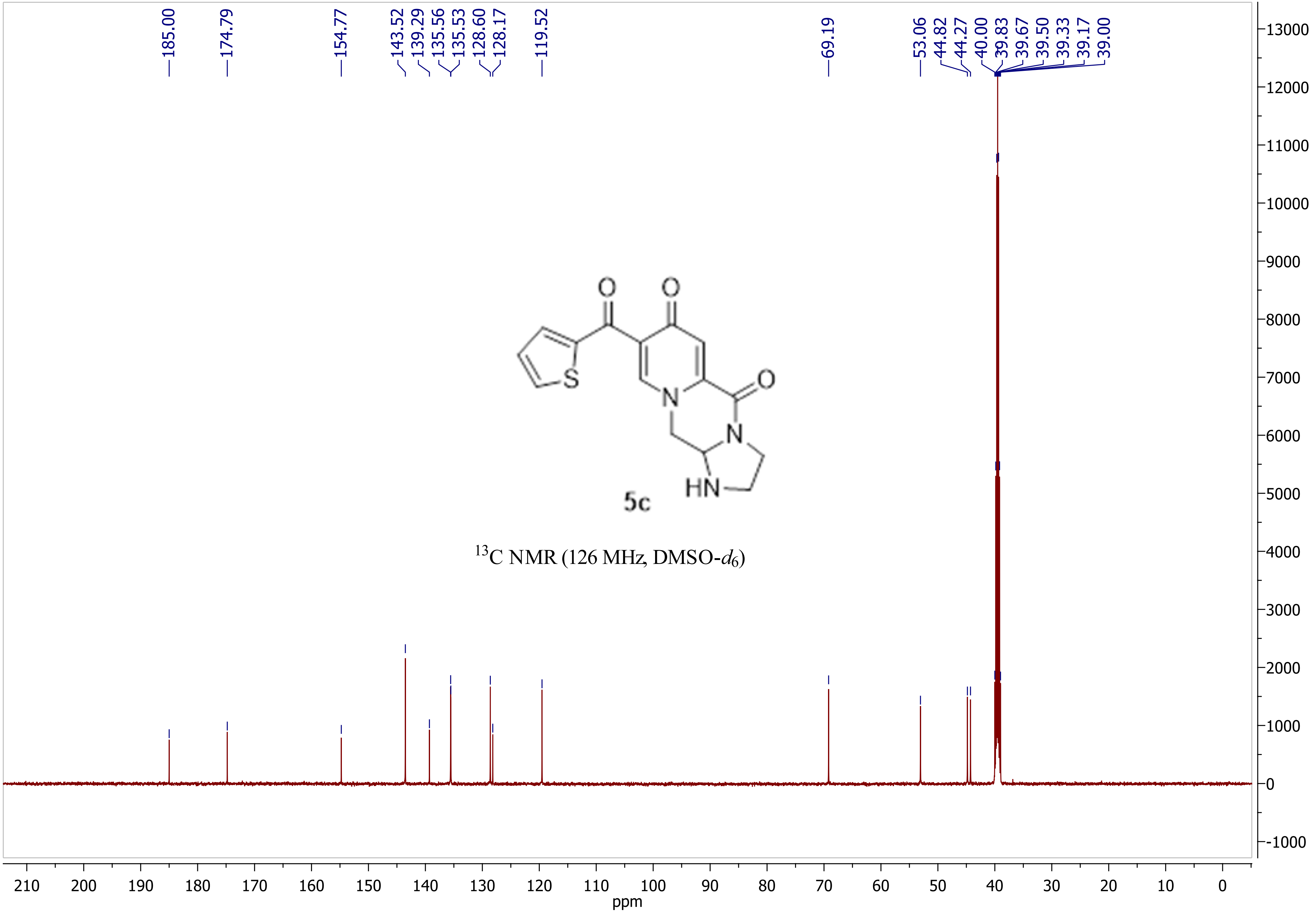
185.00
174.79
154.77
143.52
139.29
135.56
135.53
128.60
128.17
119.52
69.19
53.06
44.82
44.27
40.00
39.83
39.67
39.50
39.33
39.17
39.00
5c
O
S
N
HN
13C NMR (126 MHz, DMSO-d6)
ppm

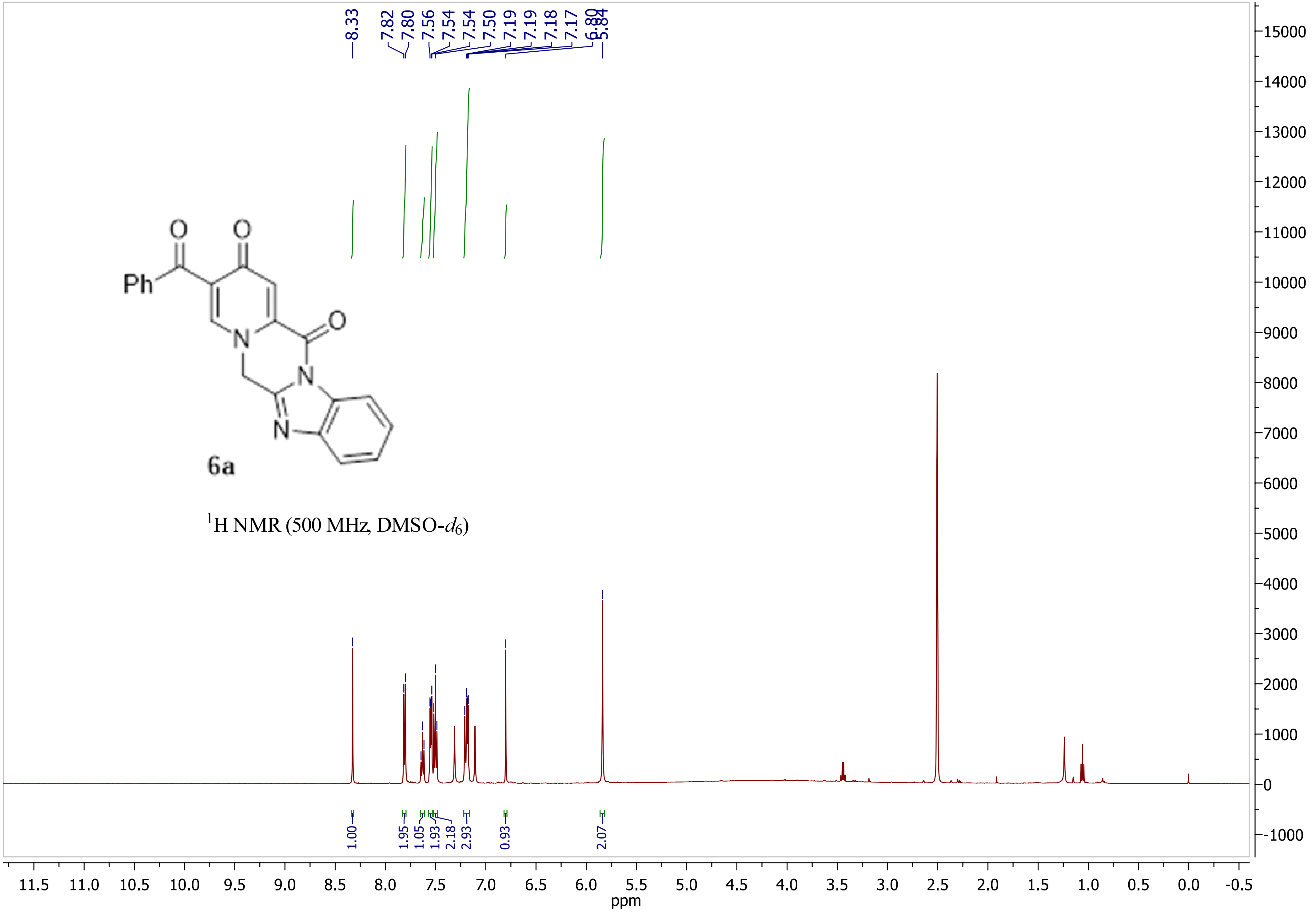
8.33
7.82
7.80
7.56
7.54
7.54
7.50
7.19
7.19
7.18
7.17
6.80
5.84
Ph
6a
1H NMR (500 MHz, DMSO-d6)
1.00
1.95
1.05
1.93
2.18
2.93
0.93
2.07
ppm

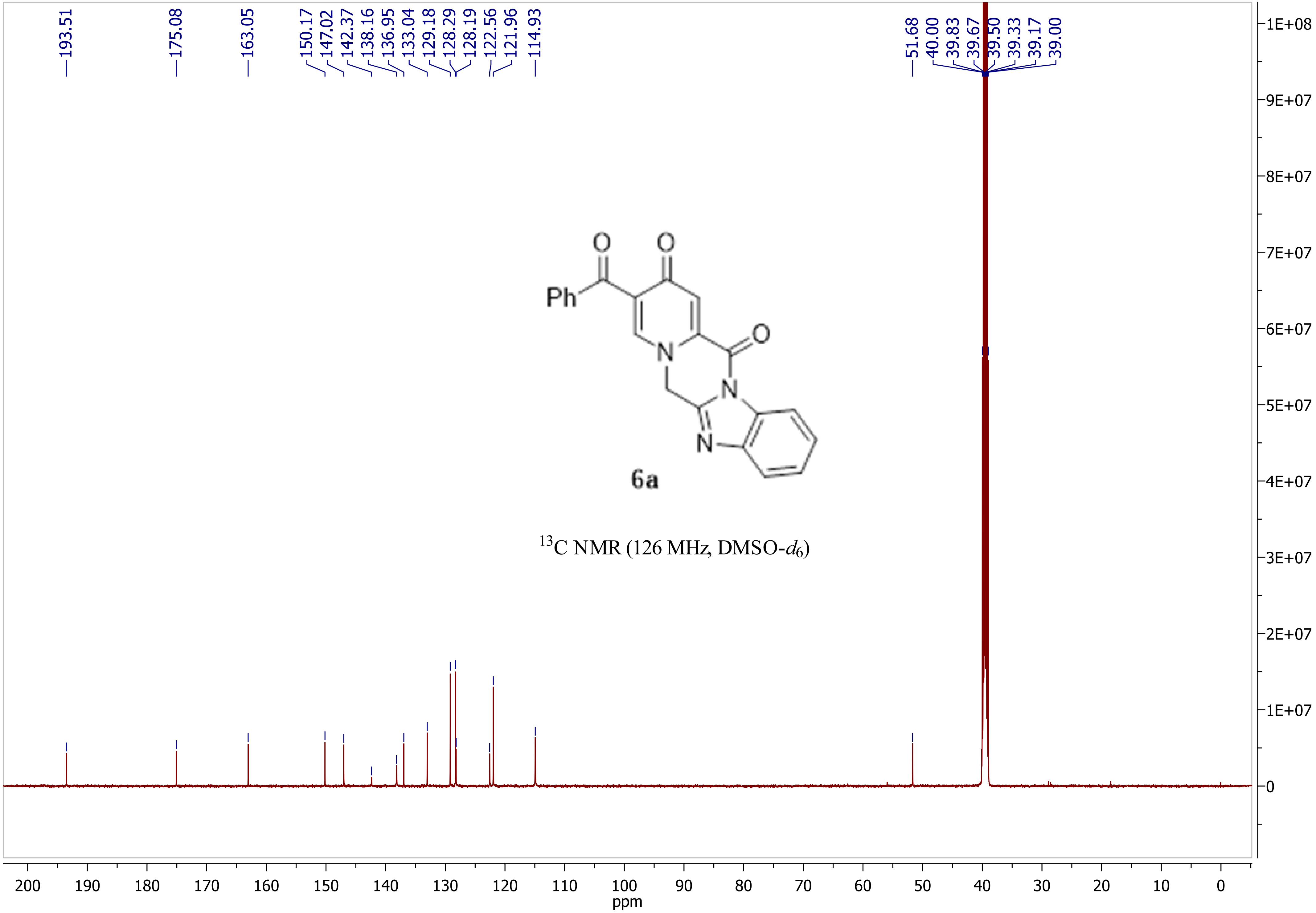
193.51
175.08
163.05
150.17
147.02
142.37
138.16
136.95
133.04
129.18
128.29
128.19
122.56
121.96
114.93
51.68
40.00
39.83
39.67
39.50
39.33
39.17
39.00
Ph
N
N
N
O
O
O
6a
$^{13}$C NMR (126 MHz, DMSO-$d_6$)
ppm

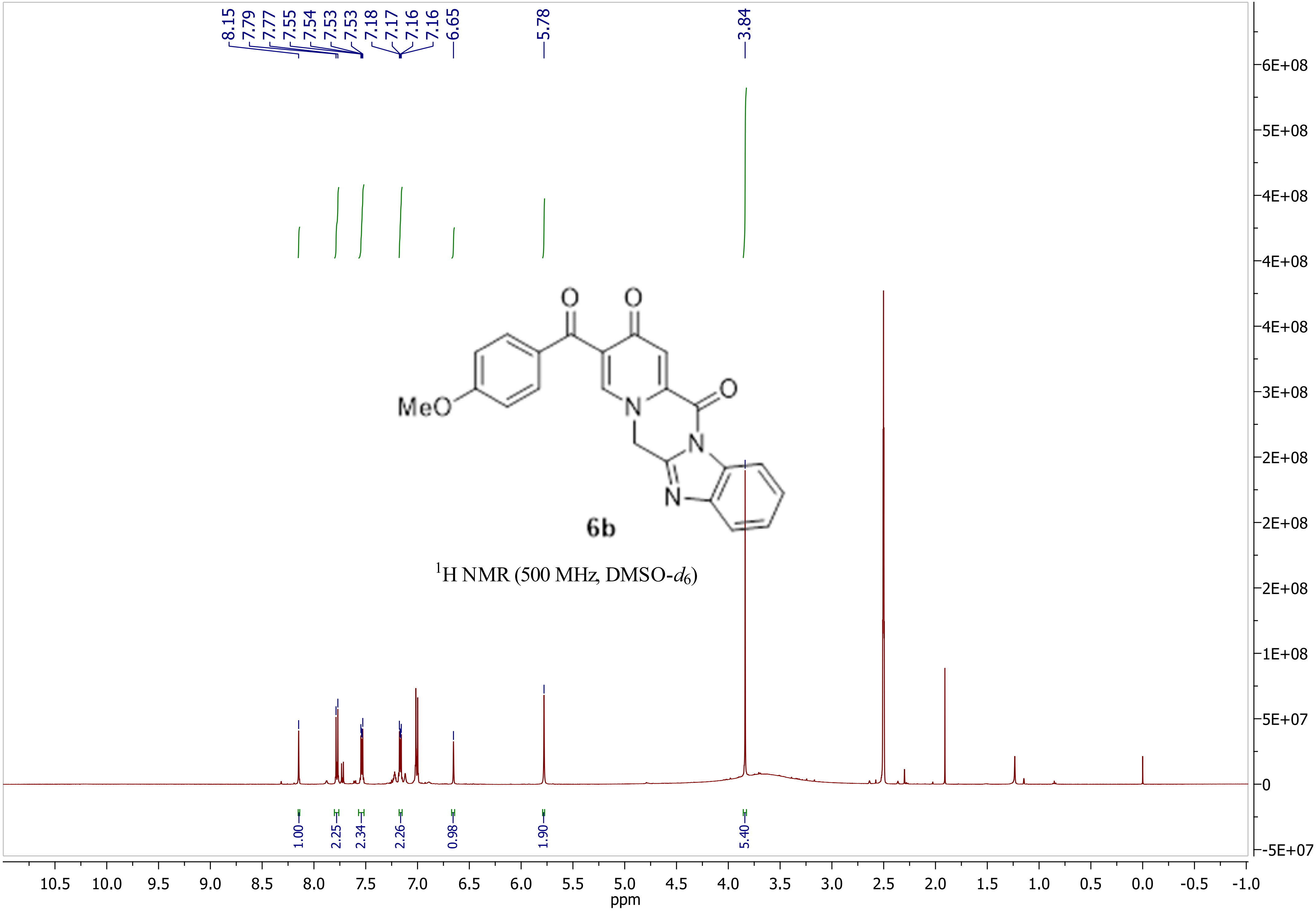

8.15
7.79
7.77
7.55
7.54
7.53
7.53
7.18
7.17
7.16
7.16
6.65
5.78
3.84
MeO
N
N
N
6b
1H NMR (500 MHz, DMSO-d6)
1.00
2.25
2.34
2.26
0.98
1.90
5.40
ppm

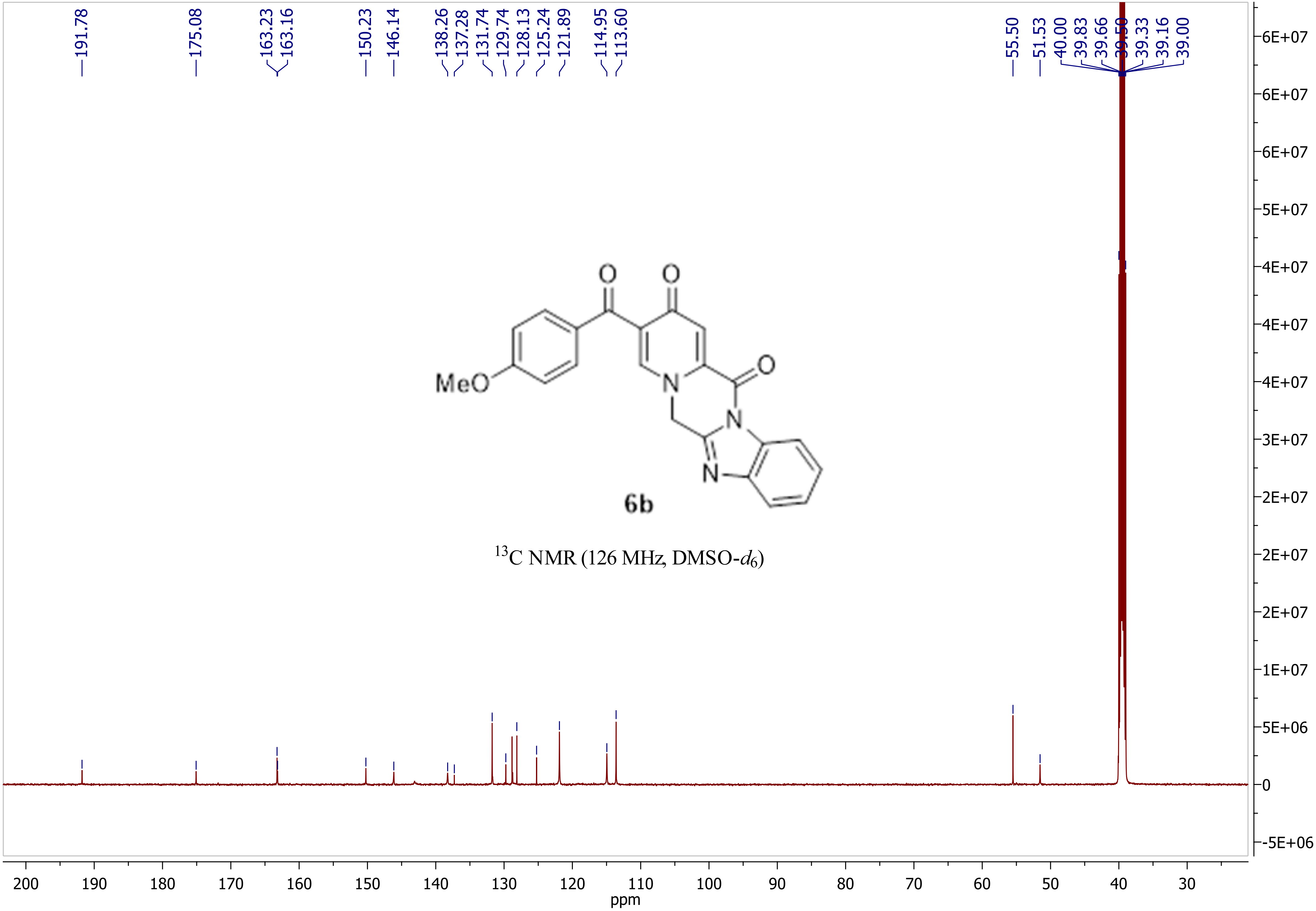


$^{13}C$ NMR (126 MHz, DMSO-$d_6$)

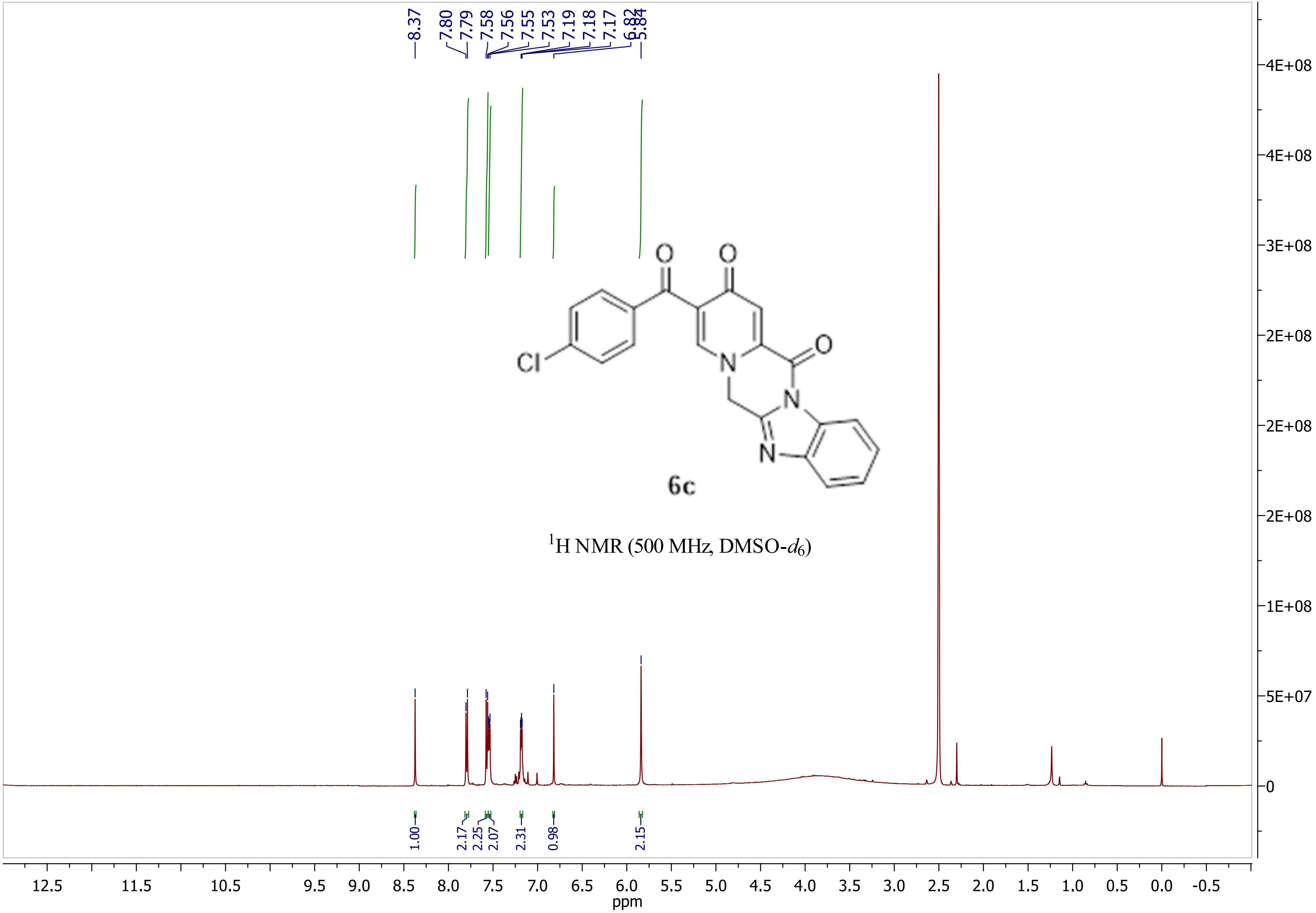
8.37
7.80
7.79
7.58
7.56
7.55
7.53
7.19
7.18
7.17
6.82
5.84
Cl
O
O
O
N
N
N
6c
1H NMR (500 MHz, DMSO-d6)
1.00
2.17
2.25
2.07
2.31
0.98
2.15
ppm

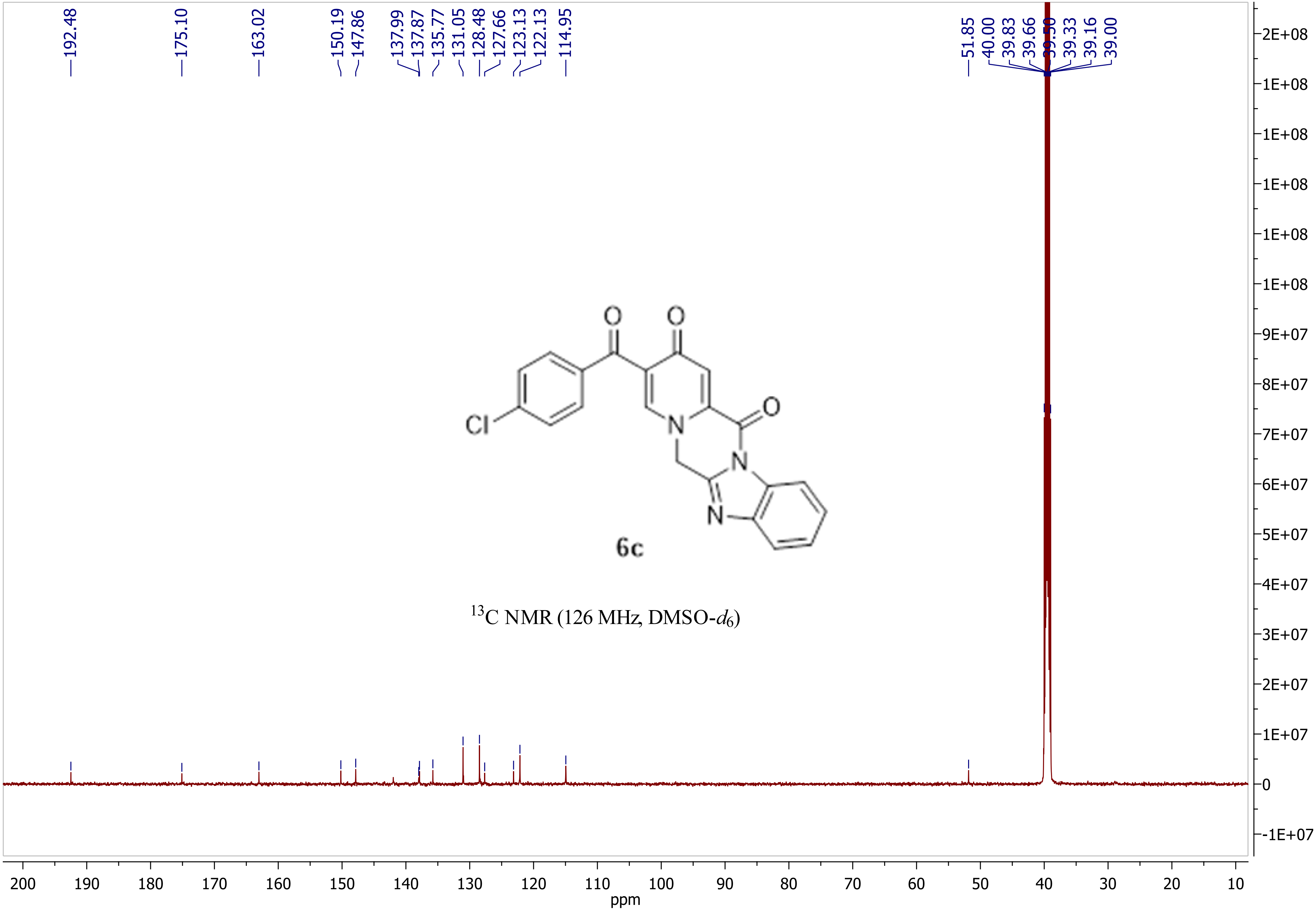
192.48
175.10
163.02
150.19
147.86
137.99
137.87
135.77
131.05
128.48
127.66
123.13
122.13
114.95
51.85
40.00
39.83
39.66
39.50
39.33
39.16
39.00
6c
$^{13}$C NMR (126 MHz, DMSO-$d_6$)
ppm

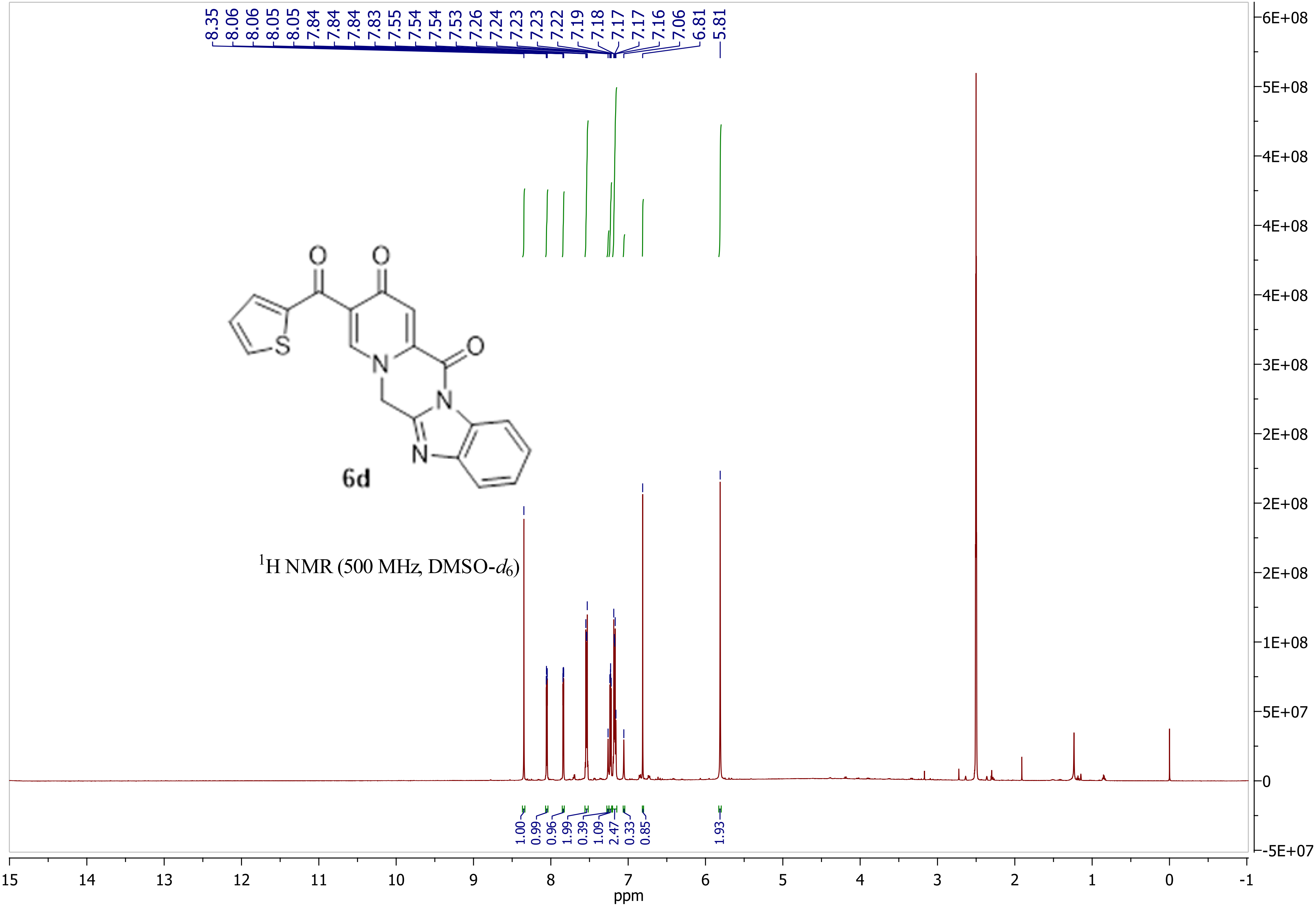
8.35
8.06
8.06
8.05
8.05
7.84
7.84
7.84
7.83
7.55
7.54
7.54
7.53
7.26
7.24
7.23
7.23
7.22
7.19
7.18
7.17
7.17
7.16
7.06
6.81
5.81
$^1$H NMR (500 MHz, DMSO-$d_6$)
1.00
0.99
0.96
1.99
0.39
1.09
2.47
0.33
0.85
1.93
ppm
15
14
13
12
11
10
9
8
7
6
5
4
3
2
1
0
-1
6E+08
5E+08
4E+08
4E+08
4E+08
3E+08
2E+08
2E+08
2E+08
1E+08
5E+07
0
-5E+07

6d

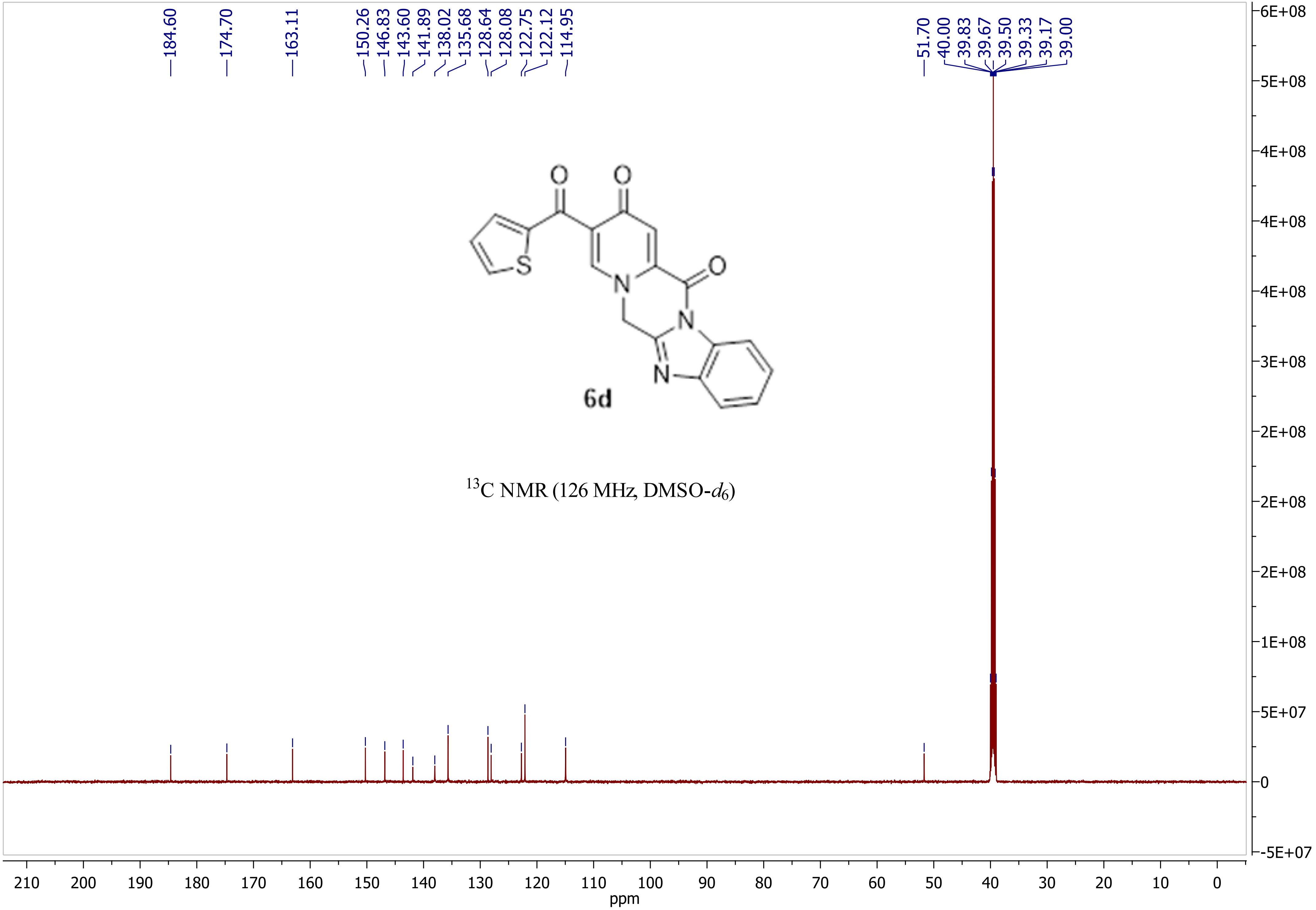
184.60
174.70
163.11
150.26
146.83
143.60
141.89
138.02
135.68
128.64
128.08
122.75
122.12
114.95
51.70
40.00
39.83
39.67
39.50
39.33
39.17
39.00
6d
13C NMR (126 MHz, DMSO-d6)
ppm

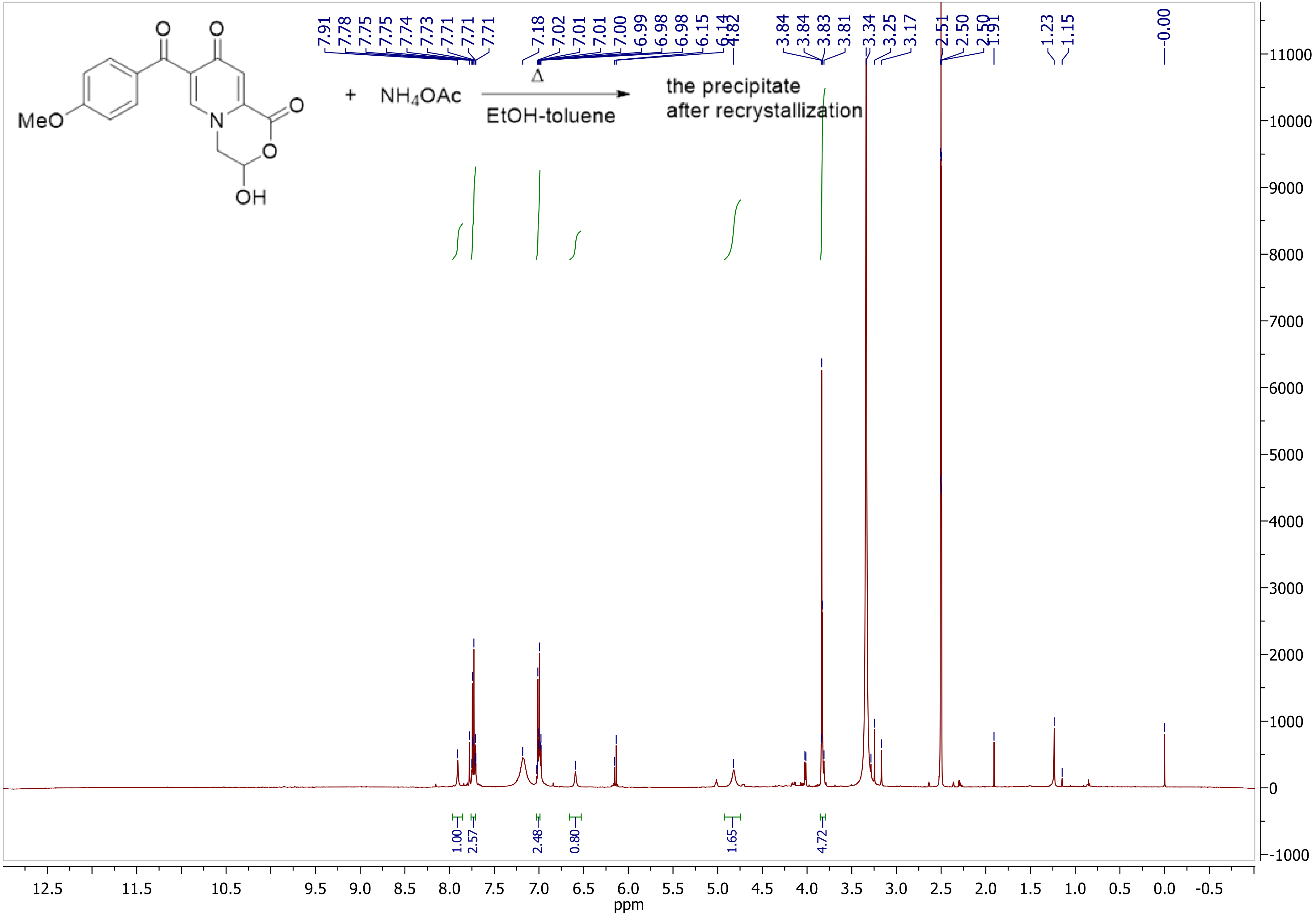
MeO
N
O
OH
+ NH4OAc
Δ
EtOH-toluene
the precipitate
after recrystallization
ppm

## Elemental Composition Report



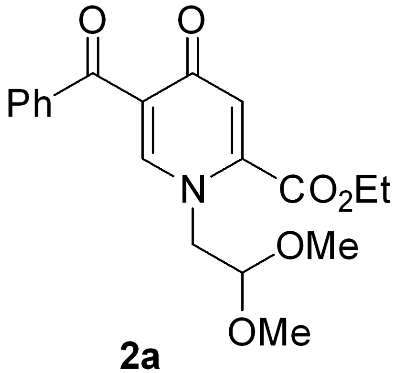


**2a**

### Single Mass Analysis
Tolerance = 5.0 PPM / DBE: min = -1.5, max = 50.0
Element prediction: Off
Number of isotope peaks used for i-FIT = 3

Monoisotopic Mass, Even Electron Ions
476 formula(e) evaluated with 2 results within limits (up to 50 closest results for each mass)
Elements Used:
C: 1-500 H: 1-1000 N: 1-10 O: 1-200
OD 25.10.2022
HRMS-CB-273 29 (0.136)

25-Oct-2022 12:42:53
TOF MS ES+
1.10e+004

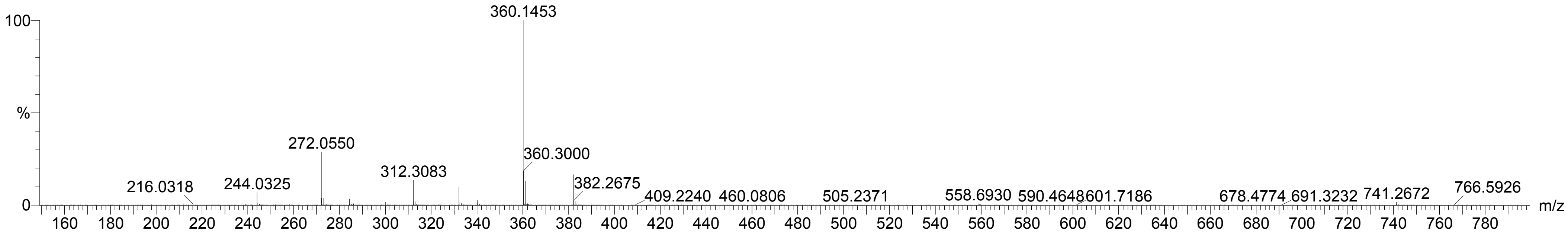


| | | | | | | | |
|---|---|---|---|---|---|---|---|
| Minimum: | | | | -1.5 | | | |
| Maximum: | | 5.0 | 5.0 | 50.0 | | | |
| Mass | Calc. Mass | mDa | PPM | DBE | i-FIT | i-FIT (Norm) | Formula |
| 360.1453 | 360.1447 | 0.6 | 1.7 | 9.5 | 94.1 | 0.6 | C19 H22 N O6 |
| | 360.1461 | -0.8 | -2.2 | 14.5 | 94.2 | 0.7 | C20 H18 N5 O2 |

# Elemental Composition Report

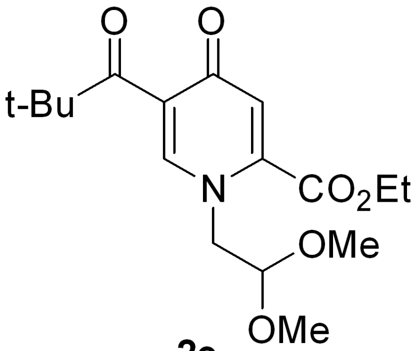


**2e**

## Single Mass Analysis

Tolerance = 5.0 PPM / DBE: min = -1.5, max = 50.0
Element prediction: Off
Number of isotope peaks used for i-FIT = 3

Monoisotopic Mass, Even Electron Ions
421 formula(e) evaluated with 2 results within limits (up to 50 closest results for each mass)
Elements Used:
C: 1-500 H: 1-1000 N: 1-10 O: 1-200
OD 25.10.2022
HRMS-CB-274 29 (0.136)

25-Oct-2022 12:47:40
TOF MS ES+
1.17e+004

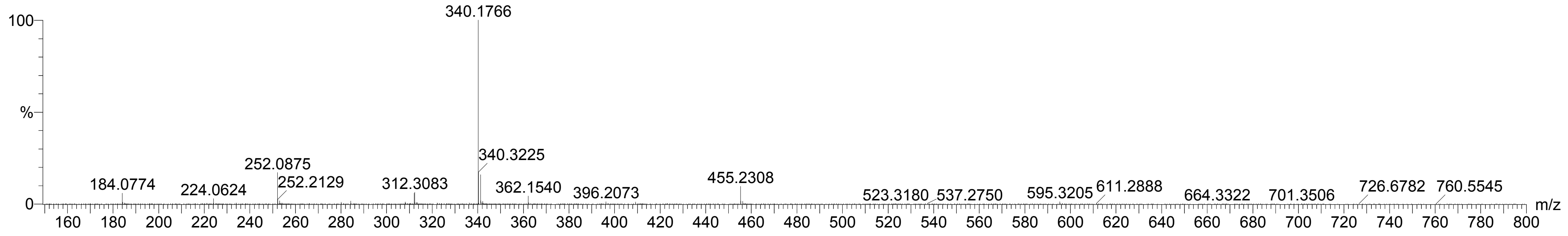


| | | | | | | | |
|---|---|---|---|---|---|---|---|
| Minimum: | | | | -1.5 | | | |
| Maximum: | | 5.0 | 5.0 | 50.0 | | | |
| Mass | Calc. Mass | mDa | PPM | DBE | i-FIT | i-FIT (Norm) | Formula |
| 340.1766 | 340.1760 | 0.6 | 1.8 | 5.5 | 92.5 | 0.5 | C17 H26 N O6 |
| | 340.1774 | -0.8 | -2.4 | 10.5 | 92.8 | 0.9 | C18 H22 N5 O2 |

## Elemental Composition Report



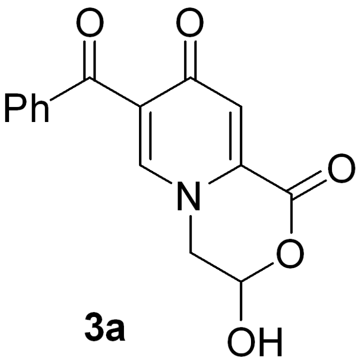


**3a**

### Single Mass Analysis

Tolerance = 5.0 PPM / DBE: min = -1.5, max = 50.0
Element prediction: Off
Number of isotope peaks used for i-FIT = 3

Monoisotopic Mass, Even Electron Ions
297 formula(e) evaluated with 1 results within limits (up to 50 closest results for each mass)
Elements Used:
C: 1-500 H: 1-1000 N: 1-10 O: 1-200
OD 28.07.2022
HRMS-CB-262 39 (0.177)

28-Jul-2022 11:55:27
TOF MS ES+
2.90e+002

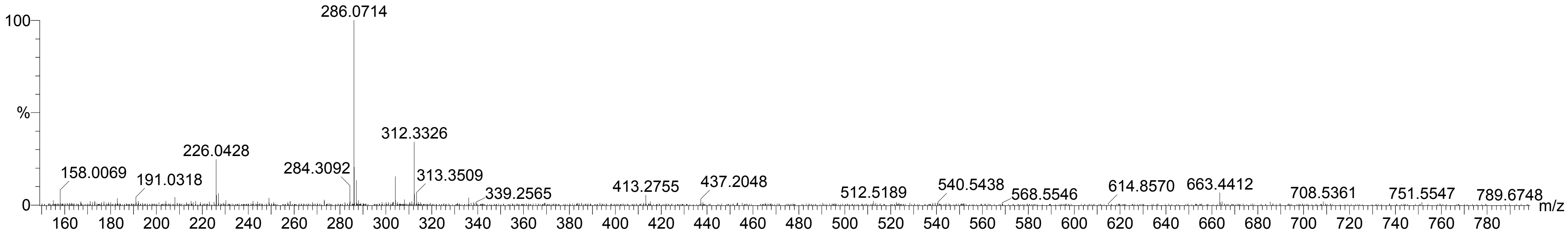


| | | | | | | | |
|---|---|---|---|---|---|---|---|
| Minimum: | | | | -1.5 | | | |
| Maximum: | | 5.0 | 5.0 | 50.0 | | | |

| Mass | Calc. Mass | mDa | PPM | DBE | i-FIT | i-FIT (Norm) | Formula |
|---|---|---|---|---|---|---|---|
| 286.0714 | 286.0715 | -0.1 | -0.3 | 10.5 | 32.4 | 0.0 | C15 H12 N O5 |

## Single Mass Analysis

Tolerance = 5.0 PPM / DBE: min = -1.5, max = 50.0
Element prediction: Off
Number of isotope peaks used for i-FIT = 3

Monoisotopic Mass, Even Electron Ions
296 formula(e) evaluated with 2 results within limits (up to 50 closest results for each mass)
Elements Used:
C: 1-500 H: 1-1000 N: 1-10 O: 1-200 Cl: 1-1
OD 25.10.2022
HRMS-CB-282 24 (0.115)

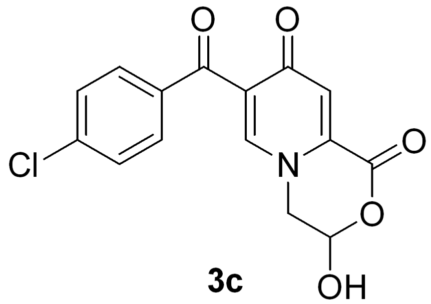


3c

25-Oct-2022 13:08:07
TOF MS ES+
4.59e+003

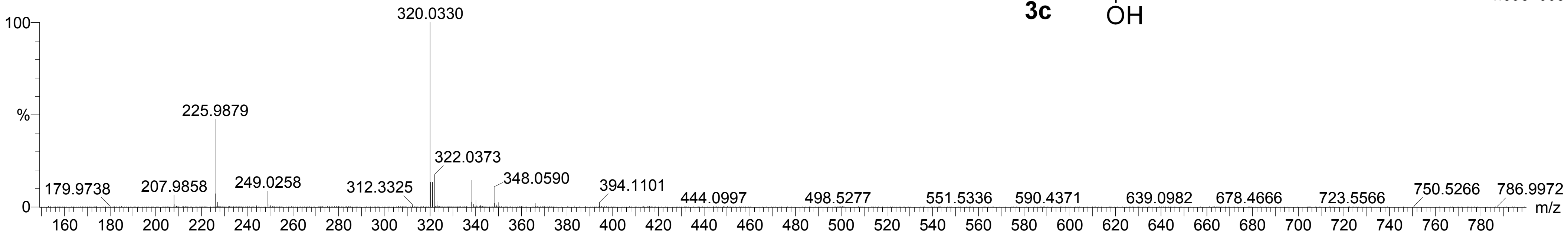


| | | | | | | | |
|---|---|---|---|---|---|---|---|
| Minimum: | | | | -1.5 | | | |
| Maximum: | | 5.0 | 5.0 | 50.0 | | | |
| Mass | Calc. Mass | mDa | PPM | DBE | i-FIT | i-FIT (Norm) | Formula |
| 320.0330 | 320.0326 | 0.4 | 1.2 | 10.5 | 67.1 | 0.7 | C15 H11 N O5 Cl |
| | 320.0339 | -0.9 | -2.8 | 15.5 | 67.0 | 0.7 | C16 H7 N5 O Cl |

## Single Mass Analysis

Tolerance = 5.0 PPM / DBE: min = -1.5, max = 50.0
Element prediction: Off
Number of isotope peaks used for i-FIT = 3

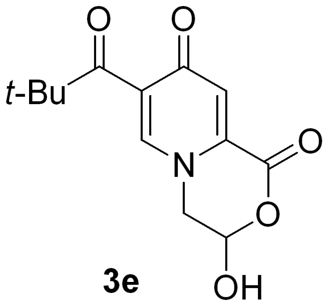

**3e**

Monoisotopic Mass, Even Electron Ions
248 formula(e) evaluated with 2 results within limits (up to 50 closest results for each mass)
Elements Used:
C: 1-500 H: 1-1000 N: 1-10 O: 1-200

OD 28.07.2022
HRMS-CB-261 26 (0.123)

28-Jul-2022 11:53:00
TOF MS ES+
1.08e+004

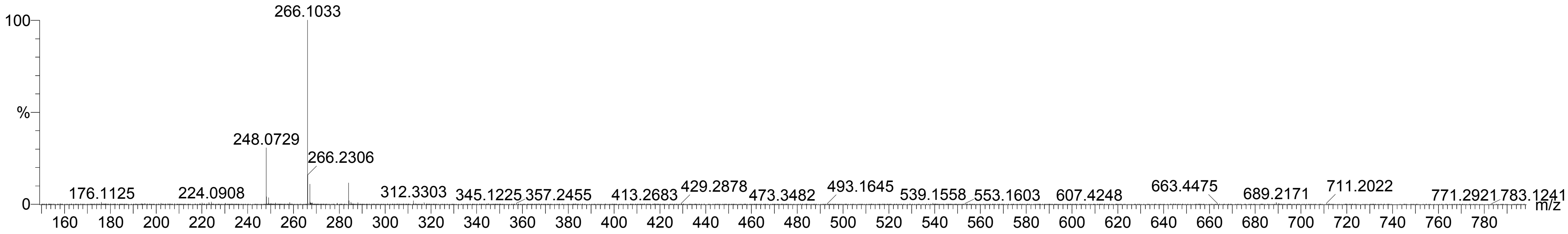


| | | | | | | | |
|---|---|---|---|---|---|---|---|
| Minimum: | | | | -1.5 | | | |
| Maximum: | | 5.0 | 5.0 | 50.0 | | | |
| Mass | Calc. Mass | mDa | PPM | DBE | i-FIT | i-FIT (Norm) | Formula |
| 266.1033 | 266.1028 | 0.5 | 1.9 | 6.5 | 101.3 | 0.5 | C13 H16 N O5 |
| | 266.1042 | -0.9 | -3.4 | 11.5 | 101.7 | 0.9 | C14 H12 N5 O |

## Single Mass Analysis

Tolerance = 5.0 PPM / DBE: min = -1.5, max = 50.0
Element prediction: Off
Number of isotope peaks used for i-FIT = 3

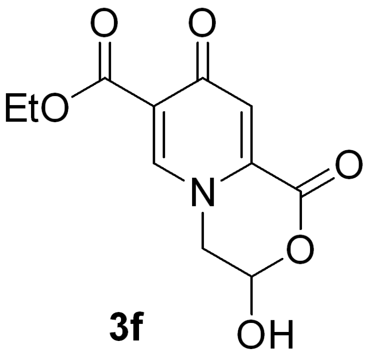


**3f**

Monoisotopic Mass, Even Electron Ions
229 formula(e) evaluated with 2 results within limits (up to 50 best isotopic matches for each mass)
Elements Used:
C: 1-500 H: 1-1000 N: 1-10 O: 1-200

OD 11.07.2022
HRMS-CB-242 49 (0.219)

11-Jul-2022 14:57:25
TOF MS ES+
1.13e+003

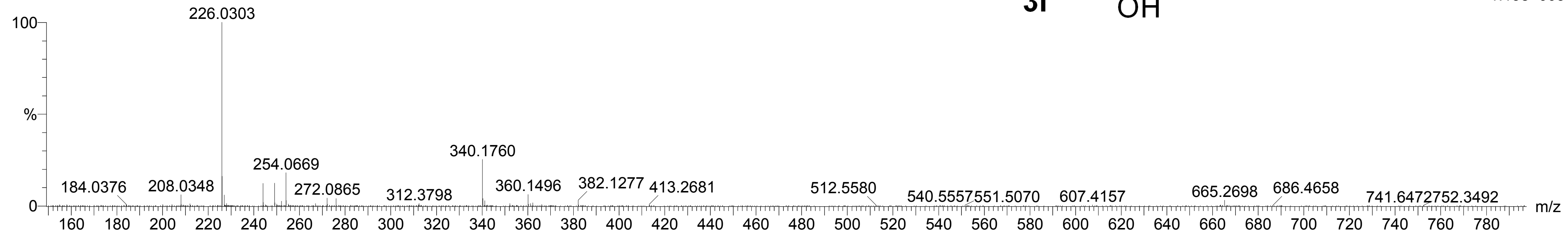


| Mass | Calc. Mass | mDa | PPM | DBE | i-FIT | i-FIT (Norm) | Formula |
|---|---|---|---|---|---|---|---|
| Minimum: | | | | -1.5 | | | |
| Maximum: | | 5.0 | 5.0 | 50.0 | | | |
| 254.0669 | 254.0665 | 0.4 | 1.6 | 6.5 | 31.7 | 0.3 | C11 H12 N O6 |
| | 254.0678 | -0.9 | -3.5 | 11.5 | 32.7 | 1.3 | C12 H8 N5 O2 |

# Elemental Composition Report



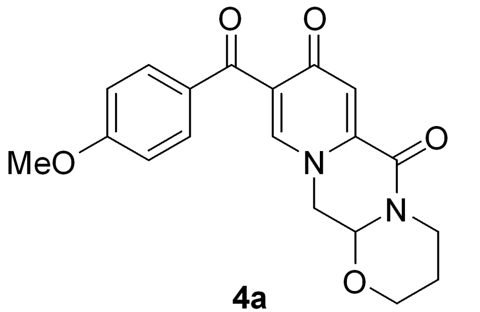


**4a**

## Single Mass Analysis

Tolerance = 5.0 PPM / DBE: min = -1.5, max = 50.0
Element prediction: Off
Number of isotope peaks used for i-FIT = 3

Monoisotopic Mass, Even Electron Ions
476 formula(e) evaluated with 2 results within limits (up to 50 closest results for each mass)
Elements Used:
C: 1-500 H: 1-1000 N: 1-10 O: 1-200

OD 25.10.2022
HRMS-CB-276 27 (0.128)

25-Oct-2022 12:52:17
TOF MS ES+
8.55e+003

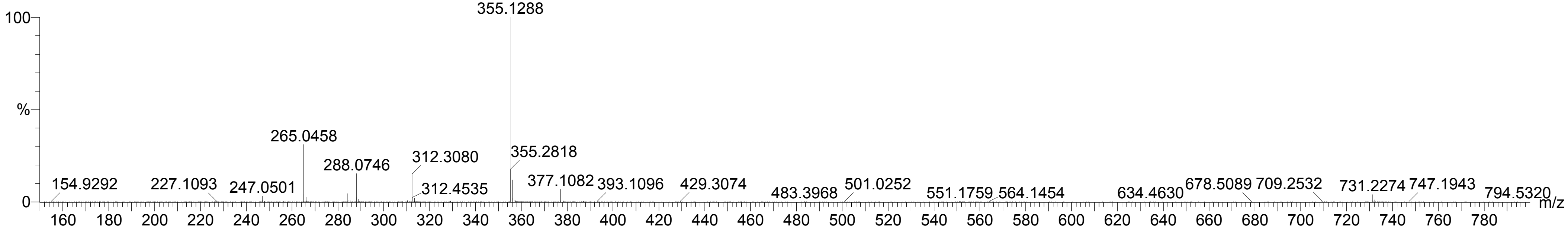


| | | | | | | | |
|---|---|---|---|---|---|---|---|
| Minimum: | | | | -1.5 | | | |
| Maximum: | | 5.0 | 5.0 | 50.0 | | | |
| Mass | Calc. Mass | mDa | PPM | DBE | i-FIT | i-FIT (Norm) | Formula |
| 355.1288 | 355.1286 | 0.2 | 0.6 | -0.5 | 62.5 | 0.2 | C3 H19 N10 O10 |
| | 355.1294 | -0.6 | -1.7 | 11.5 | 63.9 | 1.6 | C19 H19 N2 O5 |

# Elemental Composition Report

## Single Mass Analysis

Tolerance = 5.0 PPM / DBE: min = -1.5, max = 50.0
Element prediction: Off
Number of isotope peaks used for i-FIT = 3

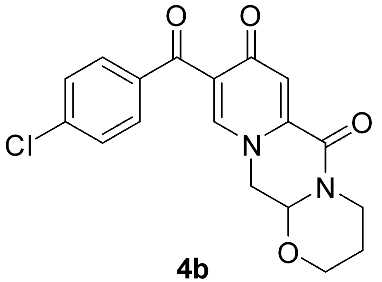

**4b**

Monoisotopic Mass, Even Electron Ions
370 formula(e) evaluated with 3 results within limits (up to 50 closest results for each mass)
Elements Used:
C: 1-500 H: 1-1000 N: 1-10 O: 1-200 Cl: 1-1

OD 25.10.2022
HRMS-CB-278 20 (0.098)

25-Oct-2022 12:56:53
TOF MS ES+
1.75e+004

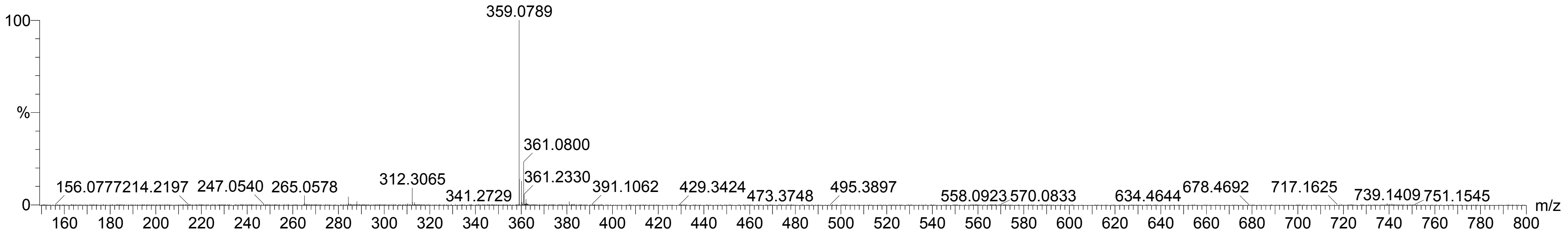


| | | | | | | | |
|---|---|---|---|---|---|---|---|
| Minimum: | | | | -1.5 | | | |
| Maximum: | | 5.0 | 5.0 | 50.0 | | | |
| Mass | Calc. Mass | mDa | PPM | DBE | i-FIT | i-FIT (Norm) | Formula |
| 359.0789 | 359.0790 | -0.1 | -0.3 | -0.5 | 106.4 | 2.3 | C2 H16 N10 O9 Cl |
| | 359.0799 | -1.0 | -2.8 | 11.5 | 104.9 | 0.8 | C18 H16 N2 O4 Cl |
| | 359.0772 | 1.7 | 4.7 | 12.5 | 104.9 | 0.8 | C14 H12 N8 O2 Cl |

# Elemental Composition Report

## Single Mass Analysis

Tolerance = 5.0 PPM / DBE: min = -1.5, max = 50.0
Element prediction: Off
Number of isotope peaks used for i-FIT = 3

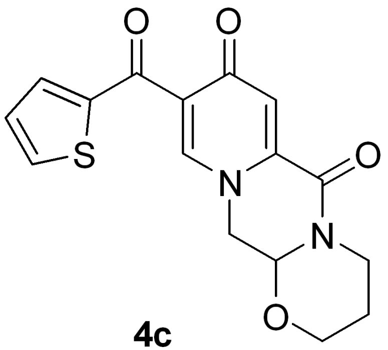


4c

Monoisotopic Mass, Even Electron Ions
336 formula(e) evaluated with 1 results within limits (up to 50 closest results for each mass)
Elements Used:
C: 1-500 H: 1-1000 N: 1-10 O: 1-200 S: 1-1

OD 25.10.2022
HRMS-CB-280 22 (0.107)

25-Oct-2022 13:03:30
TOF MS ES+
4.07e+003

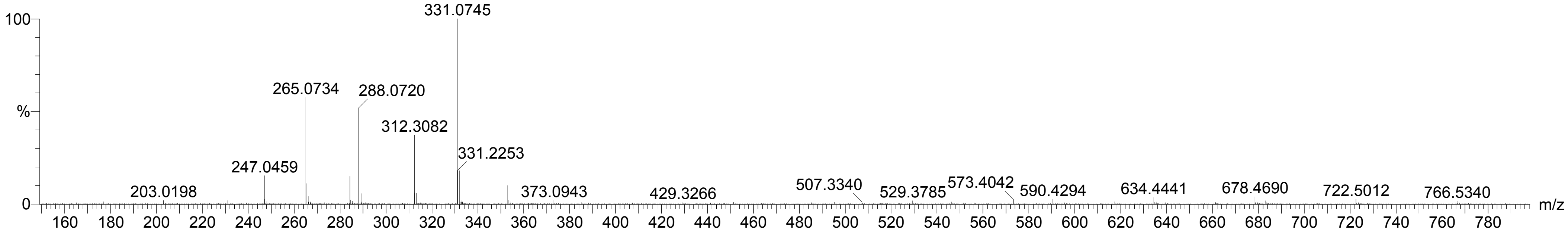


| | | | | | | | |
|---|---|---|---|---|---|---|---|
| Minimum: | | | | -1.5 | | | |
| Maximum: | | 5.0 | 5.0 | 50.0 | | | |
| Mass | Calc. Mass | mDa | PPM | DBE | i-FIT | i-FIT (Norm) | Formula |
| 331.0745 | 331.0753 | -0.8 | -2.4 | 10.5 | 69.0 | 0.0 | C16 H15 N2 O4 S |

**Elemental Composition Report**

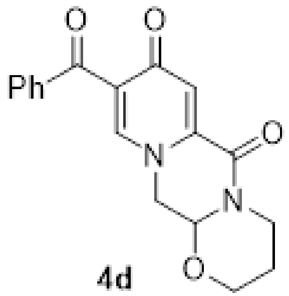

4d

**Single Mass Analysis**
Tolerance = 15.0 PPM / DBE: min = -1.5, max = 50.0
Element prediction: Off
Number of isotope peaks used for i-FIT = 3

Monoisotopic Mass, Even Electron Ions
145 formula(e) evaluated with 1 results within limits (up to 50 closest results for each mass)
Elements Used:
C: 1-500 H: 1-1000 N: 1-3 O: 1-200

OD 26.12.2022
CB-305 53 (0.236)

26-Dec-2022 17:15:36
TOF MS ES+
8.16e+002

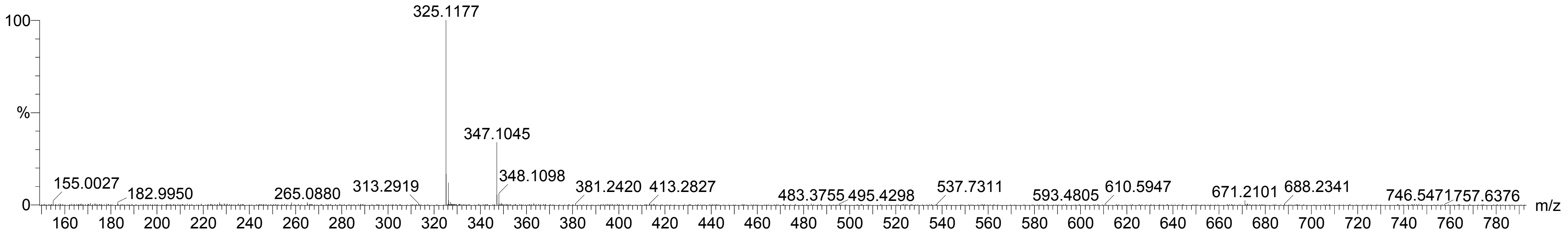


| | | | | | | | |
|---|---|---|---|---|---|---|---|
| Minimum: | | | | -1.5 | | | |
| Maximum: | | 5.0 | 15.0 | 50.0 | | | |
| Mass | Calc. Mass | mDa | PPM | DBE | i-FIT | i-FIT (Norm) | Formula |
| 325.1177 | 325.1188 | -1.1 | -3.4 | 11.5 | 50.6 | 0.0 | C18 H17 N2 O4 |

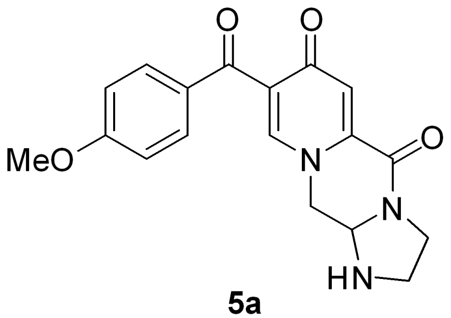

**5a**

## Single Mass Analysis

Tolerance = 5.0 PPM / DBE: min = -1.5, max = 50.0
Element prediction: Off
Number of isotope peaks used for i-FIT = 3

Monoisotopic Mass, Even Electron Ions
421 formula(e) evaluated with 2 results within limits (up to 50 closest results for each mass)
Elements Used:
C: 1-500 H: 1-1000 N: 1-10 O: 1-200

OD 25.10.2022
HRMS-CB-279 31 (0.144)

25-Oct-2022 13:01:11
TOF MS ES+
2.39e+002

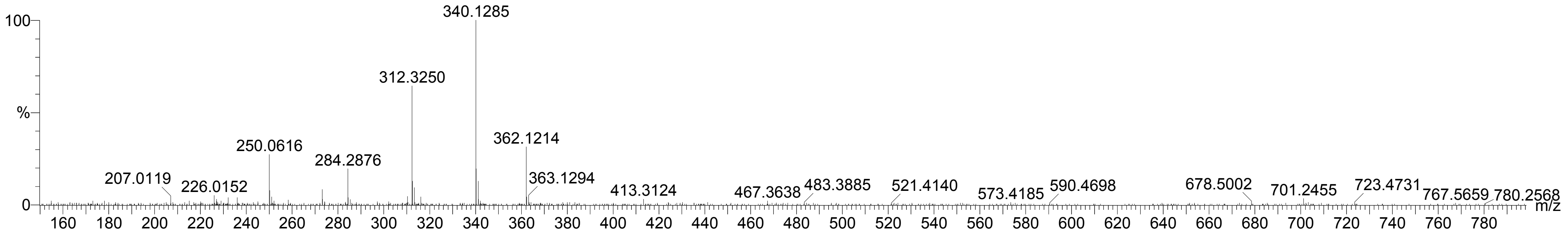


| Mass | Calc. Mass | mDa | PPM | DBE | i-FIT | i-FIT (Norm) | Formula |
|---|---|---|---|---|---|---|---|
| Minimum: | | | | -1.5 | | | |
| Maximum: | | 5.0 | 5.0 | 50.0 | | | |
| 340.1285 | 340.1297 | -1.2 | -3.5 | 11.5 | 31.4 | 0.6 | C18 H18 N3 O4 |
| | 340.1270 | 1.5 | 4.4 | 12.5 | 31.6 | 0.8 | C14 H14 N9 O2 |

## Single Mass Analysis

Tolerance = 5.0 PPM / DBE: min = -1.5, max = 50.0
Element prediction: Off
Number of isotope peaks used for i-FIT = 3

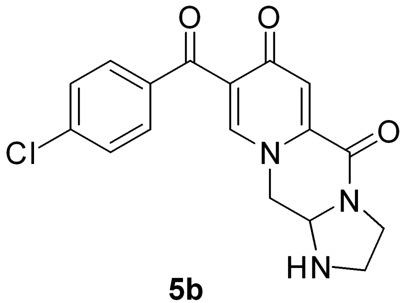


**5b**

Monoisotopic Mass, Even Electron Ions
344 formula(e) evaluated with 2 results within limits (up to 50 closest results for each mass)
Elements Used:
C: 1-500 H: 1-1000 N: 1-10 O: 1-200 Cl: 1-1

OD 25.10.2022
HRMS-CB-281 24 (0.115)

25-Oct-2022 13:05:50
TOF MS ES+
4.36e+003

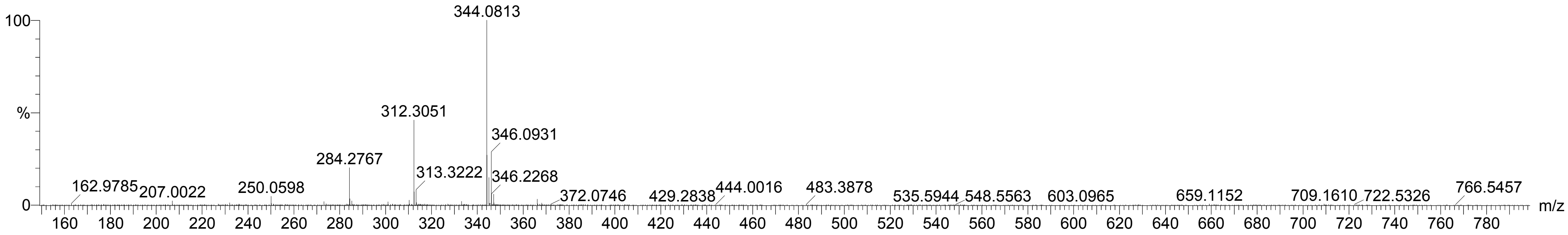


| | | | | | | | |
|---|---|---|---|---|---|---|---|
| Minimum: | | | | -1.5 | | | |
| Maximum: | | 5.0 | 5.0 | 50.0 | | | |
| Mass | Calc. Mass | mDa | PPM | DBE | i-FIT | i-FIT (Norm) | Formula |
| 344.0813 | 344.0820 | -0.7 | -2.0 | -1.5 | 72.5 | 1.6 | C5 H19 N5 O10 Cl |
| | 344.0802 | 1.1 | 3.2 | 11.5 | 71.2 | 0.2 | C17 H15 N3 O3 Cl |

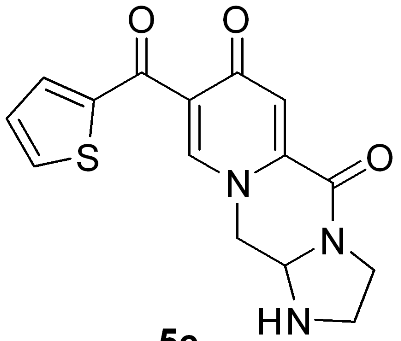


5c

## Single Mass Analysis

Tolerance = 15.0 PPM / DBE: min = -1.5, max = 50.0
Element prediction: Off
Number of isotope peaks used for i-FIT = 3

Monoisotopic Mass, Even Electron Ions
113 formula(e) evaluated with 2 results within limits (up to 50 closest results for each mass)
Elements Used:
C: 1-500 H: 1-1000 N: 1-3 O: 1-200 S: 1-1

OD 27.12.2022
CB-312 23 (0.111)

27-Dec-2022 13:32:08
TOF MS ES+
4.24e+003

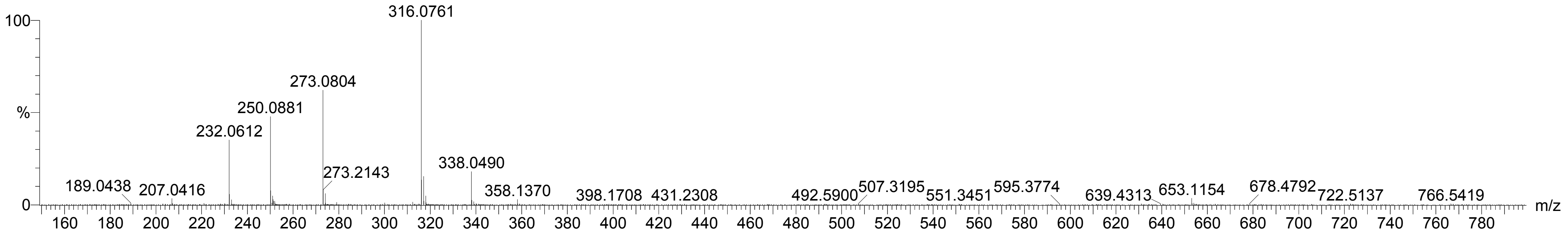


| | | | | | | | |
|---|---|---|---|---|---|---|---|
| Minimum: | | | | -1.5 | | | |
| Maximum: | | 5.0 | 15.0 | 50.0 | | | |

| Mass | Calc. Mass | mDa | PPM | DBE | i-FIT | i-FIT (Norm) | Formula |
|---|---|---|---|---|---|---|---|
| 316.0761 | 316.0756 | 0.5 | 1.6 | 10.5 | 61.5 | 0.1 | C15 H14 N3 O3 S |
| | 316.0796 | -3.5 | -11.1 | 14.5 | 64.1 | 2.6 | C20 H14 N O S |

# Elemental Composition Report

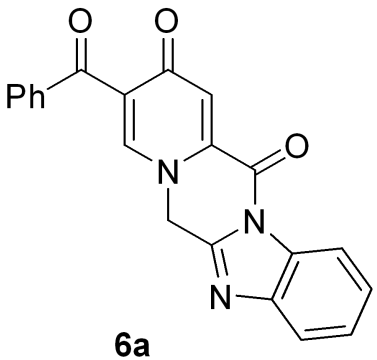


**6a**

## Single Mass Analysis

Tolerance = 15.0 PPM / DBE: min = -1.5, max = 50.0
Element prediction: Off
Number of isotope peaks used for i-FIT = 3

Monoisotopic Mass, Even Electron Ions
168 formula(e) evaluated with 4 results within limits (up to 50 closest results for each mass)
Elements Used:
C: 1-500 H: 1-1000 N: 1-3 O: 1-200

OD 27.12.2022
CB-306_2 22 (0.107)

27-Dec-2022 13:36:05
TOF MS ES+
2.80e+003

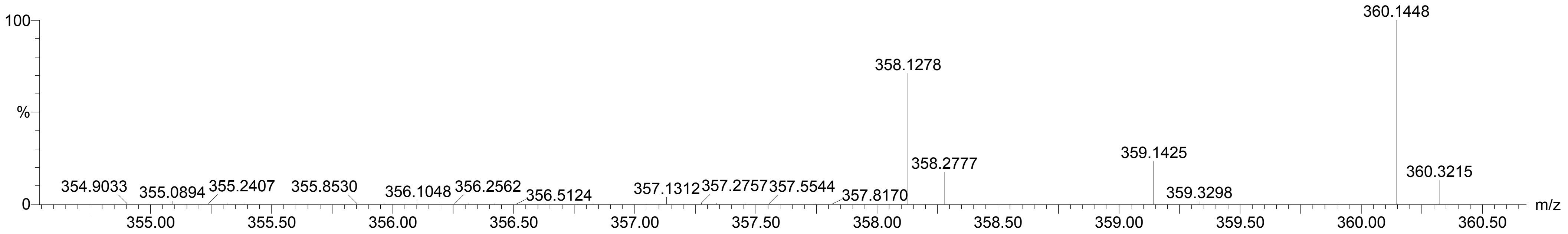


| | | | | | | | |
|---|---|---|---|---|---|---|---|
| Minimum: | | | | -1.5 | | | |
| Maximum: | | 5.0 | 15.0 | 50.0 | | | |

| Mass | Calc. Mass | mDa | PPM | DBE | i-FIT | i-FIT (Norm) | Formula |
|---|---|---|---|---|---|---|---|
| 356.1048 | 356.1040 | 0.8 | 2.2 | -1.5 | 73.4 | 2.0 | C8 H22 N O14 |
| | 356.1035 | 1.3 | 3.7 | 16.5 | 73.1 | 1.8 | C21 H14 N3 O3 |
| | 356.1075 | -2.7 | -7.6 | 20.5 | 72.4 | 1.0 | C26 H14 N O |
| | 356.1094 | -4.6 | -12.9 | 7.5 | 72.5 | 1.1 | C14 H18 N3 O8 |

# Elemental Composition Report

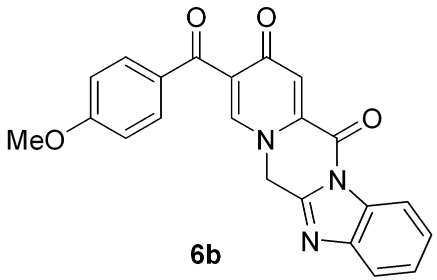


**6b**

## Single Mass Analysis

Tolerance = 15.0 PPM / DBE: min = -1.5, max = 50.0
Element prediction: Off
Number of isotope peaks used for i-FIT = 3

Monoisotopic Mass, Even Electron Ions
194 formula(e) evaluated with 4 results within limits (up to 50 closest results for each mass)
Elements Used:
C: 1-500 H: 1-1000 N: 1-3 O: 1-200

OD 27.12.2022
CB-309_2 30 (0.140)

27-Dec-2022 13:54:26
TOF MS ES+
3.48e+002

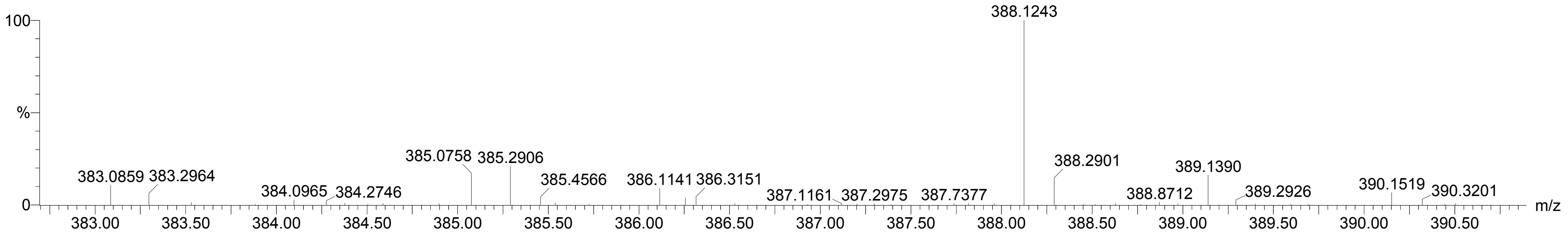


| | | | | | | | |
|---|---|---|---|---|---|---|---|
| Minimum: | | | | -1.5 | | | |
| Maximum: | | 5.0 | 15.0 | 50.0 | | | |
| Mass | Calc. Mass | mDa | PPM | DBE | i-FIT | i-FIT (Norm) | Formula |
| 386.1141 | 386.1141 | 0.0 | 0.0 | 16.5 | 54.8 | 1.8 | C22 H16 N3 O4 |
| | 386.1146 | -0.5 | -1.3 | -1.5 | 55.2 | 2.2 | C9 H24 N O15 |
| | 386.1181 | -4.0 | -10.4 | 20.5 | 53.3 | 0.3 | C27 H16 N O2 |
| | 386.1087 | 5.4 | 14.0 | 7.5 | 57.7 | 4.7 | C16 H20 N O10 |
